# Media Framing and Portrayals of Ransomware Impacts on Informatics, Employees, and Patients: Systematic Media Literature Review

**DOI:** 10.2196/59231

**Published:** 2025-04-08

**Authors:** Atiya Avery, Elizabeth White Baker, Brittany Wright, Ishmael Avery, Dream Gomez

**Affiliations:** 1 Harbert College of Business Auburn University Auburn, AL United States; 2 School of Business Virginia Commonwealth University Richmond, VA United States; 3 Russell Medical Center Laboratory Russell Medical Center Alexander City, AL United States; 4 Department of Electric Engineering and Computer Science Florida Atlantic University Boca Raton, FL United States

**Keywords:** cybersecurity, media frames, medical informatics, practitioners, health care provider, systematic review, employees, patient, mortality, morbidity, news media, ransomware, health information system, database, health care service

## Abstract

**Background:**

Ransomware attacks on health care provider information systems have the potential to impact patient mortality and morbidity, and event details are relayed publicly through news stories. Despite this, little research exists on how these events are depicted in the media and the subsequent impacts of these events.

**Objective:**

This study used collaborative qualitative analysis to understand how news media frames and portrays the impacts of ransomware attacks on health informatic systems, employees, and patients.

**Methods:**

We developed and implemented a systematic search protocol across academic news databases, which included (1) the Associated Press Newswires, (2) Newspaper Source, and (3) Access World News (Newsbank), using the search string “(hospital OR healthcare OR clinic OR medical) AND (ransomware OR denial of service OR cybersecurity).” In total, 4 inclusion and 4 exclusion criteria were applied as part of the search protocol. For articles included in the study, we performed an inductive and deductive analysis of the news articles, which included their article characteristics, impact portrayals, media framings, and discussions of the core functions outlined in the National Institute of Standards and Technologies (NIST) Cybersecurity Framework 2.0.

**Results:**

The search returned 2195 articles, among which 48 news articles published from 2009 to 2023 were included in the study. First, an analysis of the geographic prevalence showed that the United States (34/48, 71%), followed to a lesser extent by India (4/48, 8%) and Canada (3/48, 6%), featured more prominently in our sample. Second, there were no apparent year-to-year patterns in the occurrence of reported events of ransomware attacks on health care provider information systems. Third, ransomware attacks on health care provider information systems appeared to cascade from a single point of failure. Fourth, media frames regarding “human interest” and “responsibility” were equally representative in the sample. The “response” function of the NIST Cybersecurity Framework 2.0 was noted in 36 of the 48 (75%) articles. Finally, we noted that 17 (14%) of the articles assessed for eligibility were excluded from this study as they promoted a product or service or spoke hypothetically about ransomware events among health care providers.

**Conclusions:**

Organizational response represented a substantial aspect of the news articles in our corpus. To address the perception of health care providers’ management of ransomware attacks, they should take measures to influence perceptions of (1) health care service continuity, despite a lack of availability of health informatics; (2) responsibility for the patient experience; and (3) acknowledgment of the strain on health care practitioners and patients through a public declaration of support and gratitude. Furthermore, the media portrayals revealed a prevalence of single points of failure in the health informatics system, thus providing guidance for the implementation of safety protocols that could significantly reduce cascading impacts.

## Introduction

### Background

According to the United States Cybersecurity and Infrastructure Security Agency, ransomware is a form of malware designed to encrypt files on a device, rendering any files and the systems that rely on them unusable, after which malicious actors will demand ransom in exchange for decryption [1]. Compared with data breach events [2-4], ransomware events can be even more insidious [5-7]. In 2019, an Alabama hospital was locked out of its computers for 8 days, unable to access patient records, locate staff, or monitor fetal heartbeats, cutting doctors and nurses off from critical patient information, equipment, and resources [8]. At the time of this writing, there is an ongoing lawsuit alleging that the ransomware attack led to the death of an infant, as it obscured access to fetal heartbeat monitoring that would have alerted the attending physician that the umbilical cord had wrapped around the infant’s neck during delivery, resulting in severe brain damage and the death of the infant 9 months later—an outcome the lawsuit claims could have been prevented had the physician performed a lifesaving cesarean section [9,10]. Another example highlighted by the *Washington Post* is a ransomware attack in September 2020 that targeted Düsseldorf University in Germany, subsequently impacting a hospital that was connected to the university’s information system [11,12]. The hospital information system became inaccessible, which led to the death of a 78 year old female patient with aneurysm but had to be diverted to a hospital 1 hour away [13]. According to our data, these were the first documented deaths tied directly to cybersecurity events in hospital information systems, both of which were ransomware attacks [10,12]. Details about the unintentional and accidental deaths of hospital patients due to ransomware attacks were relayed to the public through news stories [8,13].

The media has a profound impact on the public’s understanding of issues, and extensive research in this area has revealed how media coverage influences people’s learning, knowledge, and perceptions about a particular topic [14]. Media coverage can raise awareness about specific issues, providing explanations and “framing” to influence audiences’ understanding of the issues [15]. Framing is a technique that involves the use of media frames to invite audiences to view issues, actions, and events in one particular way over another, thus helping audiences to create their sense of reality [16]. Media frames, particularly news media frames, can directly impact public policy and sway public opinion [17]. News media framing has been directed to the public regarding various issues and topics, such as autism, feminism, the COVID-19 pandemic, crises, and environmental issues, among others [18-22].

Ransomware attacks on health care providers have demonstrated that cybersecurity threats have the potential to be severe public health threats. These events have proven to be targeted operational disruptions compared to other types of cybersecurity events, such as data breaches [23,24]. Operational disruptions occur predominantly through the unavailability of health informatics systems, including electronic health records (EHRs), practice management software, patient portals, electronic billing and scheduling systems, and electronic communications between health care professionals and patients, which are essential to carrying out daily practice operations and meeting standards of care [25]. In the Western context, almost all health care workflows are digital and rely on robust digital infrastructures [26]. A ransomware attack can, therefore, have significantly negative consequences for patients, including disruption of patient care, which may consequently threaten patient outcomes [27]. Despite these serious risks, little research has been conducted on the use of media framing and portrayals of the impacts of ransomware events on health care providers. Although media framing has been explored in various other health care contexts [20,28-32] it is essential to understand the impacts of news media framing and portrayals of these events on health care practitioners, patients, and health informatics for 3 important reasons.

First, despite the plethora of industry association reports and white papers with sources of information on ransomware available to health care providers, information about the current news media landscape regarding this issue could provide health care providers with a better understanding of how to manage the publicity of these events. For instance, news stories report on how health care providers manage their health care informatics systems and how they handle cybersecurity attacks. In addition, news stories of ransomware attacks on health care providers can inform leadership on how to develop more robust operational continuity plans, as media framing used in crisis communication can provide managers with insight into how to establish appropriate crisis response strategies [33]. By understanding the role of news media on public perception through understanding media framing in the context of ransomware attacks on health care providers, crisis managers can strategize how to use publicity to minimize damage to the organization’s reputation following such an attack [34]. In the context of health care, news media can be used to provide reinforcements that help to maintain patient trust. In addition, news media reports describe events in laypeople’s terms, which can improve the public’s understanding of issues related to health informatics systems. In the 2019 Alabama ransomware case mentioned previously, the mother asserted in her lawsuit against the health care providers that had she been informed about the ransomware attack and the subsequent disruption of the health informatics systems, she would have opted for an alternative provider for her daughter’s birth [10], which ostensibly would have led to a different outcome.

Second, research on news media portrayal of ransomware attacks on health informatics systems is vital for frontline health care practitioners, as those practitioners who are not formal members of leadership might not be privy to information about the organization as it may be shared on a need-to-know basis, with these practitioners having to rely on news stories for information. In addition, health care providers located in close proximity to another health care provider impacted by a ransomware attack could experience impacts from that event. One study that reported increased patient traffic at a hospital due to diversions from a hospital that experienced a ransomware attack [27]. News stories provide health care providers with information about the extent of a nearby ransomware event, which can help them to prepare. Understanding how news stories of ransomware attacks on health care providers may be portrayed and framed by media organizations can provide practitioners within and outside the affected organization with additional insights into how to use this source of information.

Third, warfare has transitioned from the battleground into attacks on the information technology and systems of critical infrastructure organizations, such as health care providers. These types of organizations are vulnerable to nation-state and domestic attacks, with the intent being loss of life and personal harm rather than monetary outcomes. In addition to managing the direct impacts of the ransomware attack, organizations may need to manage coordinated and uncoordinated disinformation campaigns related to the event. Managing disinformation campaigns is critical, as social norms, values, organizational pressures, constraints, interest groups, stakeholders, journalist routines, ideological or political leanings, and media organizations could impact how media is framed [14]. Importantly, disseminating misinformation and disinformation presents a significant global public health challenge [35,36]. News media are typically viewed as a source of reliable information; however, when combined with social media, the information they disseminate may lead to unforeseen consequences on public health, patient care, and frontline practitioners. Hence, there is an urgent need to understand the use of news media framing in portrayals of ransomware attacks on health care providers during the social media era, which has compromised the public’s perceptions of the news media as an original, trustworthy source.

### Objectives

In this review, we explore these knowledge gaps by evaluating media frames and their portrayals of the impacts of ransomware and denial of service attacks on health care providers through a search of top news media sources from around the world. Our research goals were as follows:

- To reveal patterns in news media framing of ransomware attacks on health care information systems
- To understand how news media portrays the impacts of health care information system ransomware attacks on patient care and health care providers
- To gain insights on media portrayals of the cybersecurity management aspects of ransomware attacks on health informatics systems

The aim of this study was to understand how news media frames and portrays the impacts of ransomware attacks on health information systems, employees, and patients.

## Methods

### Search Strategy

Our search strategy was guided in part by the PRISMA (Preferred Reporting Items for Systematic Reviews and Meta-Analyses) 2020 [37] guidelines, which is predominately used for the systematic review of research studies and was adopted as a guide for our systematic search of news databases. It is important to clarify that our unit of analysis is news articles, not research studies, so some parts of the protocols may not be applicable. Multimedia Appendices 1 and 2 contain the research protocols for PRISMA-S: an extension to the PRISMA statement for reporting literature searches in systematic reviews and standards for reporting qualitative research.

On September 15, 2023 we conducted a comprehensive literature search of 3 news databases, from their inception until the date of the search: Access World News, also referred to as NewsBank; Newspaper Source; and the Associated Press Newswire. September 15, 2023 was used as the cut-off date for identified records. Access World News collates news articles from over 3500 sources worldwide, including transcripts from television news stories [38]. Newspaper Source provides cover-to-cover full text for hundreds of US, international, and regional newspapers, television, and radio news transcripts from major networks [39]. Some subscriptions of Newspaper Source may include the Associated Press Newswire database. The Associated Press Newswire is a full-text database that contains harvested news from the Associated Press, and when the user clicks a link to a news story, they are sent to the original article [40]. We used the following search string to ensure an exhaustive and broad but relevant return of records: “(hospital OR healthcare OR clinic OR medical) AND (ransomware OR denial of service OR cybersecurity).” To ensure that only the most relevant results were returned, we searched all the text within articles obtained from Newswire and Newspaper Source and searched only the lead or first paragraph of articles obtained from Access World News. An English language filter was applied to all database searches. The default settings were used for all other database settings, including the Year filter, and no additional search filters or constraints were applied. We used the term “news articles” to refer to all news media, including radio and television news transcripts, if they were stored in an article format and in the news databases. Duplicate records were removed manually and by Covidence (Veritas Health Innovation Ltd) systematic review software.

### Inclusion and Exclusion Criteria

Inclusion criteria were developed before our database search. Specific inclusion criteria included (1) articles in the English language; (2) articles with full text available; (3) articles containing a health care provider context, noted as articles related to a hospital, doctors, nursing staff, clinics, nursing homes, medical practitioners, nutritionists, and dieticians; and (4) ransomware or denial of service attack. To eliminate irrelevant articles, the specific exclusion criteria were (1) articles unrelated to a specific health care provider or unrelated to a ransomware or denial of service attack, (2) articles that primarily focused on the promotion or advertisement of a product or consulting service, (3) articles unrelated to a specific ransomware or denial of service attack, or (4) articles with only broad discussions of health care and ransomware or denial of service attacks. Examples of excluded articles are ones that included discussions on pure data breach events or medical device vulnerabilities. First, 2 of the authors, AA and BW, independently screened titles and abstracts of all news articles for eligibility in duplicate. Next, the full text of the remaining news articles was examined to determine if the articles met the inclusion criteria. Next, articles that were not relevant based on the exclusion criteria were excluded. Any disagreements between AA and BW were discussed until they were resolved.

### Data Extraction and Coding

Data extraction and coding of news articles was performed between September 16, 2023 and January 21, 2024. First, we extracted the news media characteristics from each article to include the (1) focal health care provider organization, (2) country, (3) year, (4) attack type, and (5) perspective. Detailed characteristics extracted from each news story are present in Multimedia Appendix 3. Next, we conducted our content analysis. Our research method incorporated an inductive and deductive approach for our content analysis of news articles, first by deductively analyzing the media frames related to ransomware attacks on health care providers to understand how these events were portrayed for the public and inductively analyzing the media portrayals of the impacts on the health informatics systems, patients, and employees to understand how the impacts of these events are portrayed to the public. Our inductive approach involved examining news articles with an open view to allow themes to emerge, whereas our deductive approach consisted of analyzing the articles for predefined themes, which in this study were media frames and cybersecurity core functions [41,42].

In addition, we used a collaborative qualitative analysis (CQA) method as outlined in the study by Richards and Hemphill [43], which was used for both inductive and deductive qualitative research. The benefits of CQA include the integration of perspectives to increase trustworthiness and the counteraction of individual bias by multiple researchers. In total, 4 authors participated in the CQA, including AA, BW, IA, and DG. EWB assisted with the interpretation of the results. The authors of this study are from diverse backgrounds, and each offer a unique perspective to the research study, which is why we applied CQA. The research team included a laboratory scientist (BW); an emergency room physician who is also a full-time computer science master’s student specializing in artificial intelligence and cybersecurity (IA); an information systems researcher holding CompTIA Security+ and Certified Information Systems Security Professional Certification (AA); a seasoned former systems engineer turned information systems doctoral student (DG); and an expert on the adoption, safety, effectiveness, and design of medical mobile app tools, such as clinical decision support systems and patient self-care systems, working at the intersection of health care and cybersecurity (EWB).

AA, BW, IA, and DG were involved in the CQA process and followed the recommendations to (1) perform a 6-phase qualitative analysis process, (2) develop rapport among researchers before the start of the analysis, (3) reinforce that all researchers made a valuable contribution and their perspectives are valued, and (4) provide a constant comparison of newly coded data with previously coded data to modify generative themes in light of challenging or contradictory data.

Specifically for our inductive approach, 2 authors, AA and BW, extracted the impacts from each of the 48 articles after extracting the news article characteristics, cross-checking their extractions for completeness, and frequently debriefing the information. The extraction focused on ensuring a complete description of the impacts. Next, a third author, IA, cross-checked the extraction of impacts against the articles to derive coherent notes. The initial 2 authors, AA and BW, then conducted a final review of the impact notes from IA. Notes related to the extraction of impacts are located in Multimedia Appendix 4. After extracting the impacts from each of the news articles, AA, BW, IA, and DG reviewed the impacts to determine if there were any patterns. All authors agreed that the media portrayals of impacts from ransomware attacks on health care providers showed a cascading pattern; in other words, the impacts did not occur independently, in that the unavailability of one or more components of a health care informatics system led to further impacts. After an examination and discussion of other potential organizing schemas and taxonomies, all researchers agreed that the cascading pattern most accurately depicted the relationship of impacts described in media portrayals of ransomware attacks on health care providers.

Next, AA, BW, IA, and DG used a deductive approach to code the news articles for media frames according to the media framing categories developed by Semetko and Valkenburg [44], which included “conflict,” “human interest,” “economic consequences,” “morality,” and “responsibility” frames. To begin the deductive coding process, AA, BW, IA, and DG selected a random sample of 5 articles that they independently coded. AA, BW, IA, and DG then debriefed and resolved any disagreement before each researcher independently coded the remaining articles in duplicate. AA, BW, IA, and DG used an iterative process of debriefing and resolving conflicting frames based on the coding dictionary depicted in Table 1, until a consensus was reached [43]. AA, BW, IA, and DG each documented their rationale for assigning one or more media frames to an article. AA, BW, IA, and DG combined and summarized notes (frame code summary notes) from the coding process and the subsequent discussions to facilitate this. Then, AA, BW, IA, and DG documented the initial frame code summary notes for each article. Next, AA, BW, IA, and DG ensured the completeness and accuracy of the frame code summaries, cross-checking with each other as necessary. The final media frames assigned to each article are presented in Multimedia Appendix 5, along with the finalized frame code summary notes.

**Table 1:** Media frames (adapted from the study by Semetko and Valkenburg [44]).

Frame code	Definition	How to tell?
Conflict frame=1	This frame emphasizes conflict between individuals, groups, or institutions as a means of capturing audience interest	Does the story reflect disagreement between parties, individuals, groups, or countries? Does one party-individual-group-country reproach another? Does the story refer to 2 sides or >2 sides of the problem or issue?
Human interest frame=2	This frame brings a human face or an emotional angle to the presentation of an event, issue, or problem	Does the story provide a human example or “human face” on the issue? Does the story use adjectives or personal vignettes that generate feelings of outrage, empathy, caring, sympathy, or compassion? Does the story emphasize how individuals and groups are affected by the issue or problem? Does the story go into the private or personal lives of the actors? Does the story contain visual information that might generate feelings of outrage, empathy, caring, sympathy, or compassion?
Economic consequences frame=3	This frame reports an event, problem, or issue regarding the consequences it will have economically on an individual, group, institution, region, or country	Is there a mention of financial losses or gains now or in the future? Is there a mention of the costs or degree of expense involved? Is there a reference to the economic consequences of pursuing or not pursuing a course of action?
Morality frame=4	This frame puts the event, problem, or issue in the context of religious tenets or moral prescriptions	Does the story contain any moral message? Does the story make reference to morality, God, or other religious tenets? Does the story offer specific social prescriptions about how to behave?
Responsibility frame=5	This frame presents an issue or problem in such a way as to attribute responsibility for its cause or solution to either the government or to an individual or group	Does the story suggest that some level of government can alleviate the problem? Does the story suggest that some level of the government is responsible for the issue or problem? Does the story suggest a solution to the problem or issue? Does the story suggest that an individual or group of people in society is responsible for the issue or problem?

The National Institute of Standards and Technologies (NIST) Cybersecurity Framework (CSF) 2.0 is intended for organizations of all sizes and industries, regardless of cybersecurity or technical maturity levels. It is noted that the NIST CSF 2.0 should be used alongside a broader enterprise or organizational risk management program. The NIST CSF 2.0 is intended to be widely understood by a wide range of stakeholders, regardless of their cybersecurity background. The NIST CSF 2.0 has 6 core functions: govern, identify, protect, detect, respond, and recover. Govern is at the center of the framework, as it details how the other core functions should be implemented. Referencing the NIST CSF 2.0 [45], the authors AA, BW, and DG extracted details about the core cybersecurity functions of governance, identify, protect, detect, respond, and recover from each of the 48 articles, cross-checking their extractions for completeness, and frequently debriefing.

### Ethical Considerations

No human participants were involved in this study. All extracted data are anonymous or deidentified. All the study data involved are publicly available and published news articles are present in accessible library databases.

## Results

### Extracted News Media Characteristics

**Figure 1:**
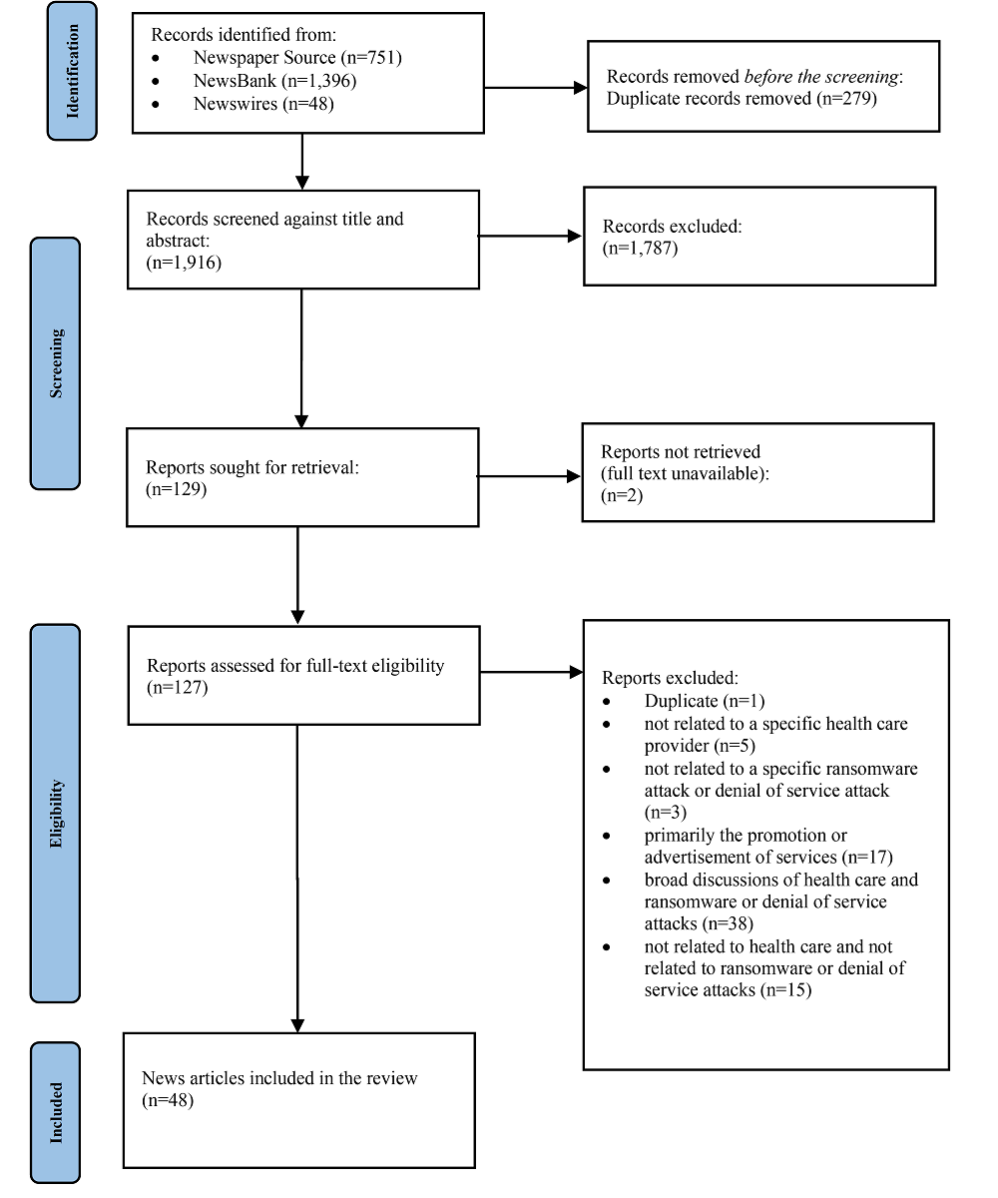
PRISMA (Preferred Reporting Items for Systematic Reviews and Meta-Analyses) diagram of news databases.

In total, 2195 news articles were returned from our keyword search. Of the 2195 articles returned, 48 news articles were included in our final analysis. The PRISMA diagram is provided in Figure 1. The primary news media characteristics are shown in Table 2; additional characteristics of interest are provided in Multimedia Appendix 3. The United States was the most frequently represented country. Approximately 36 out of 48 (75%) news articles noted an event as a ransomware attack and 12 out of 48 (25%) news articles instead opted to take on the organization’s self-description of the event. For example, one health care entity referred to its ransomware attack as a “cybersecurity incident,” and this is the term used in the article. Approximately 47 out of 48 (98%) of new articles focused on the perspective of either patients or employees or a combination of both. No articles meeting the inclusion or exclusion criteria were found before 2009, between 2010 and 2014, and during 2020. The year 2023 is partial until September 15.

**Table 2:** News media characteristics (N=48).

Characteristic				Articles, n (%)
**Health care entities represented**				
	Heritage Valley Health System			6 (13)
	All India Institute of Medical Sciences			4 (8)
	Hollywood Presbyterian			3 (6)
	Medstar			3 (6)
	National Health Service			3 (6)
	Cheyenne Regional Medical Center			2 (4)
	Hospital Sisters Health			2 (4)
	Humber River Hospitals			2 (4)
	CommonSpirit Health			2 (4)
	Not specified			2 (4)
	All other			19 (40)
**Countries represented**				
		United States	34 (71)	
		India	4 (8)	
		United Kingdom	4 (8)	
		Canada	3 (6)	
		Australia	1 (2)	
		Costa Rica	1 (2)	
		Israel	1 (2)	
**Year**				
		2009	1 (2)	
		2015	1 (2)	
		2016	9 (19)	
		2017	12 (25)	
		2018	2 (2)	
		2019	3 (6)	
		2021	3 (6)	
		2022	9 (19)	
		2023	8 (17)	
**Attack type**				
		Ransomware	36 (75)	
		Other	12 (25)	
**Perspective**				
		Employees only	6 (12)	
		Patients only	19 (40)	
		Patients and employees	22 (46)	
		Patients and researchers	1 (2)	

### Media Portrayals of Impacts

Full articles are located in Multimedia Appendix 6 by article ID number. Regarding access to electronic systems, EHR patient portals enable patients to view test results, communicate with their care team, schedule appointments, and participate in telehealth visits. Portal use can improve the satisfaction and engagement of both clinicians and patients [46,47]. When EHRs are unavailable, it can impose hardships, leading to cascading impacts from an initial ransomware attack. Although the initial impact of ransomware may be described generically as the unavailability of ≥1 components of the health informatics system, the description of actual initial impacts is quite nuanced and specific. For example, media reports may have described the ransomware attack as impacting communication systems, others as an IT outage, and others as an inability to access the software apps needed to do their work (see article ID #9, #269, #89 in Multimedia Appendix 6).

Secondary impacts stemming from the unavailability of one or more components of a health care provider’s information technology infrastructure include the inability to access patient records or send messages, the inability to accept patients, the need for patient diversion, and, in one case, the permanent shutdown of an entire medical practice (see article ID #16 in Multimedia Appendix 6).

**Figure 2:**
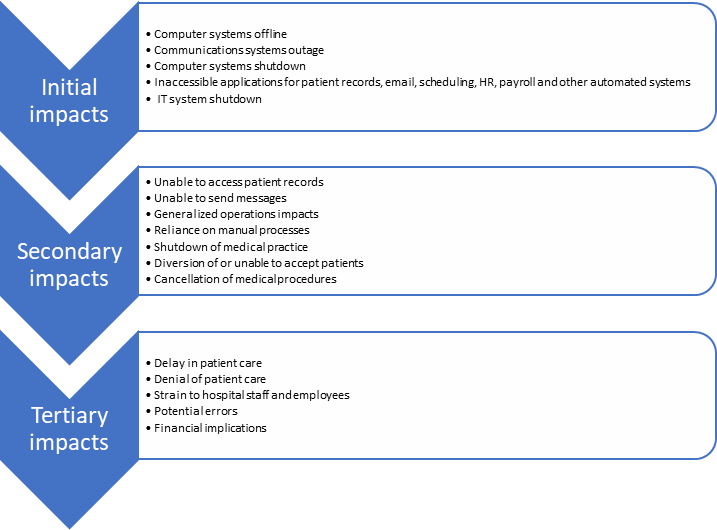
Cascading impacts. HR: human resources.

Tertiary impacts from ransomware attacks on health care entities include delays and denial of patient care; financial consequences for the organization, patients, and, in some cases, employees; and the potential for an increase in errors. Other more intangible impacts include strain on hospital staff and employees. Figure 2 depicts the cascading impacts from ransomware attacks on health care entities.

### Media Frames

Table 3 presents the results of our analysis of media frames. Discussions among the coders allowed us to address reliability and validity concerns. Although news articles could be tagged with >1 media frame code, only about 11 of 48 (23%) news articles were annotated with more than one media frame. The most representative media frames were the human interest and responsibility frames, which were equally represented in the corpus of news articles. News articles with the human interest media frame focused prominently on specific impacts on patient care, which included poignant descriptions. Most of the articles with the responsibility frame focused on swift actions that health care providers took immediately after an event. The remaining media framing around responsibility centered on government action or the attribution of the ransomware attack to a specific perpetrator.

**Table 3:** Media frame occurrence results (N=48 articles).

			Articles, n (%)
**Media frame**			
	Conflict frame	3 (6)	
	Human interest frame	15 (31)	
	Economic consequences frame	4 (8)	
	Morality frame	0 (0)	
	Responsibility frame	15 (31)	
**Co-occurring media frames**			
	Conflict and responsibility	2 (4)	
	Human interest and economic consequences	3 (6)	
	Human interest, economic consequences, and responsibility	1 (2)	
	Human interest and responsibility	4 (8)	
	Economic consequences and responsibility	1 (2)	

### Media Portrayals Based on NIST CSF 2.0

Table 4 depicts the media portrayals of ransomware management phases for health care informatic systems and the percentage of articles corresponding to each NIST CSF 2.0 core function.

**Table 4:** Media portrayals of ransomware management phases for health care informatic systems based on the National Institute of Standards and Technology CSF 2.0 (NIST CSF 2.0) (N=48).

NIST CSF 2.0 core function	NIST CSF 2.0 core function: brief description	Relationship description	News article example	Articles, n (%)
Govern	The organization’s cybersecurity risk management strategy, expectations, and policies are established, communicated, and monitored	The article discusses an existing cybersecurity management program, enterprise risk management program, cybersecurity strategy, roles, responsibilities, authorities, policies, or oversight.	A government run response service participates in the cyber risk management process (article ID #688 in Multimedia Appendix 6)	11 (23)
Identify	The organization’s current cybersecurity risks are understood	The article discusses or alludes to an understanding of organization assets or improvement opportunities for policies, plans, processes, and procedures.	Working internally and with external partners to understand the evolving cyber threat landscape (article ID #37 in Multimedia Appendix 6)	12 (25)
Protect	Safeguards are used to manage the organization’s cybersecurity risks	The article discusses the technical and nontechnical protections for the health informatic systems before, during, and after the ransomware attack, including any potential failures of protections already in place.	Rapidly shutting down systems to prevent further damage and slowly bringing them back up (article ID #150 in Multimedia Appendix 6)	14 (29%
Detecting	Possible cybersecurity attacks and compromises are found and analyzed	The article discusses how ransomware was detected in health informatics systems.	Internal and external security personnel detected a ransomware attack (article ID #206 in Multimedia Appendix 6)	16 (33)
Respond	Actions regarding a detected cybersecurity incident are taken	The article discusses how the organization responded or did not respond to the failure of the informatics.	Releasing an official or unofficial statement regarding the ransomware event. Employees or patients detailing the organization’s response or lack thereof (article ID #346 in Multimedia Appendix 6)	36 (75)
Recover	Assets and operations affected by a cybersecurity incident are restored	The article discusses how the organization maintained continuity when the health informatics failed.	Health care providers resorted to pen and paper and manual processes (article ID #534 in Multimedia Appendix 6)	19 (40)

We found that approximately 36 out of 48 (75%) news articles discussed the organization’s response or lack thereof to the unavailability of health informatics systems during a ransomware attack. In addition to official statements regarding the ransomware events, details on organizational response were also provided directly from employees and patients who had direct contact with news media organizations. In addition, we found that almost 19 out of 48 (40%) news articles discussed health informatics continuity with descriptions of resorting to pen and paper, manual processes, and diversion to other health care providers when the health informatics system was unavailable. Multimedia Appendix 7 presents a detailed article ID coding crosswalk for each NIST CSF 2.0 core function.

## Discussion

### Principal Findings

This study involved a systematic review of news articles to examine media frames and portrayals of the impacts of ransomware events on health care providers. Our findings provide insights into how to manage the health care informatics artifact, employees, and patients during a ransomware event. The 5 principal findings from this study were centered on news media characteristics, year-to-year patterns, points of failure, cascading impacts, and media frame predominance, findings that could shed light on important implications for the management of health services, including improving the quality, guiding governance, or providing insight into cybersecurity policymaking in health information technology.

First, our analysis of the geographic prevalence showed that 34 of 48 (71%) news articles from the United States, followed to a lesser extent by India with 4 (8%) news articles, and 3 (6%) news articles from Canada, featured prominently in our sample. While data on geographic significance are important, our finding was likely the result of constraining the search to English language articles; however, it is possible for the same article to be written in another language. It is also important to note that the size and economic impact of the US health care sector are also potential factors that contributed to this finding. Further research is needed to determine if this pattern holds true in a search that expands beyond English-language articles, which could lead to insights into the global significance of ransomware attacks on health care information systems. As emerging economies digitize their health care systems, it will be important to understand the role of news media if and when adverse events occur and how the use of media frames impacts local perceptions of patient care in different locations around the world.

Second, there are no apparent year-to-year patterns regarding news articles about ransomware events on health care providers. In other words, our sample showed no upward or downward pattern in media coverage of ransomware attacks on health care providers. However, there were 2 spikes in the year-to-year patterns. The first spike occurred between 2016 and 2017, which could be explained by the Hollywood Presbyterian and the Heritage Valley Health system attacks (see Multimedia Appendix 6 article IDs #168, #692, #15, #384, #543). A unique aspect of the Hollywood Presbyterian ransomware attack was that it involved public disclosure that a payment had been made from the hospital to the perpetrator. In addition, in the case of the Heritage Valley Health System ransomware attack, the organization appeared to use the media to provide updates on service availability. The second spike marked a rise in the total number of news articles on ransomware attacks on health care information systems, for which no single predominant event was responsible for the surge in media coverage that occurred between 2022 and 2023. This finding aligned with a report that the 2023 calendar year showed a 74% increase in the number of ransomware attack claims across all US industries as compared to 2022 [48]. Factors underlying this increase include a higher frequency of ransomware attacks and well-publicized ransomware campaigns exploiting 0-day vulnerabilities, an especially dangerous type of attack with few countermeasures. Ransomware attacks that specifically targeted the United States health care sector rose 128% in 2023 over 2022 [48]. It will be important in future research to empirically untangle the relationships between ransomware attack frequency and news media coverage.

**Figure 3:**
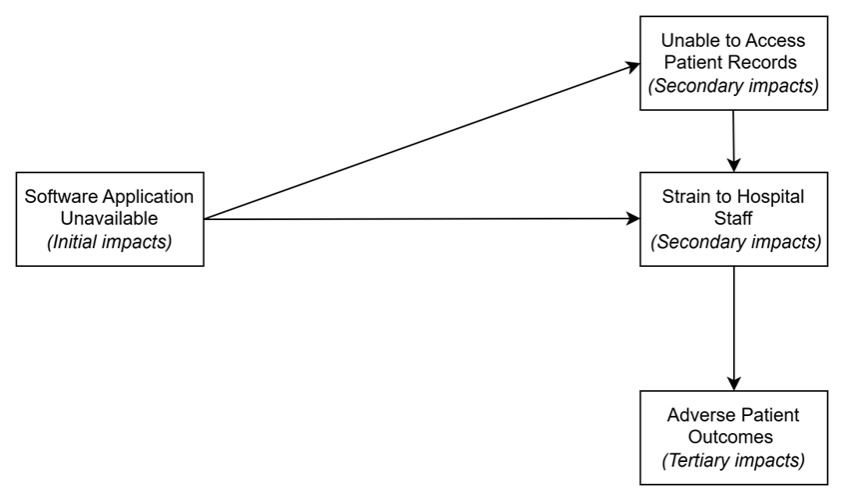
An example of the impact pathway.

Third, during our analysis, we found that health informatics system failures were caused by a single point of failure, which led to other cascading failures that were linked to adverse patient outcomes. Initially, the secondary and tertiary impacts stemming from a single ransomware event included impacts on patient care, errors, and strain on employees, among others. Figure 3 presents an exemplary depiction of a ransomware attack’s pathway of impact on a health care provider. It is important to note that strain on staff does not always lead to adverse patient outcomes. It is possible for the staff to absorb any potential shocks to patients.

Legally, health care organizations are to take immediate action and provide transparent communication on ransomware attacks in their aftermath [49]. However, there is some variance in the mandatory reporting of ransomware attacks as required by the Office of Civil Rights under the Health Information Technology for Economic and Clinical Health Act, for which designated privacy officers must disclose these types of attacks. Walden et al [50] provide an in-depth discussion of this phenomenon. Organizations are not required to report attacks classified under the Health Information Technology for Economic and Clinical Health guidelines at low exposure of personal health information (PHI). In addition, there may be uncertainty about the reporting duty when ransomware encrypts PHI data rather than removing it during the attack. Furthermore, current reporting requirements lack any enforcement mechanism or penalty for noncompliance, which has led to estimates that up to 20% of ransomware attacks go underreported [28]. When these attacks are reported, the current reporting requirements specify only a very limited amount of information, which ultimately affects the objective data that media organizations can use to report ransomware attacks [28]. This potential lack of transparency on the part of the health care service providers becomes a liability when affected individuals are looking for information on how an attack that has exposed their PHI impacts them, often turning to the media for more guidance and information.

As mentioned previously, the type of initial point of failure in a health informatics system significantly varied among the news articles. However, this may have been caused by different terms used to describe the same type of event, which raises the question of whether standardization in vocabulary is needed for media reporting of these kinds of events. With a standardized vocabulary, organizations can accurately describe the ransomware or denial of service attack. In one study, researchers noted that hospitals had significantly lower cybersecurity ratings than Fortune 500 companies and were statistically vulnerable to botnets, spam, and malware, highlighting the need for further research on the types of attacks health care systems experience and whether those events have been accurately described using the correct terminology [51]. Future research is needed to elucidate the potential benefits of using a standardized vocabulary.

Fourth, we found the human interest and responsibility media frames were equally predominant at each representing approximately 15 of 48 (31%) news articles. In addition, almost 11 of the 48 (23%) news articles had co-occurring media frames, with human interest and responsibility commonly occurring together, followed by the co-occurrence of human interest and economic consequences. When considering the challenges of developing adequate cyber defense strategies against ransomware attacks to be a sociotechnical problem, one that involves people, organizations, and technology, the social component of cyber defense will incorporate aspects of human interest and responsibility rather than just addressing the technical sophistication of security when communicating about the impacts of ransomware attacks. This aligns with the fact that 36 out of 48 (75%) news articles focused on the organization’s response to the ransomware event, and almost 19 out of 48 (40%) news articles focused on recovery in the context of the unavailability of the health care informatics artifact. By knowing the media frames that are commonly used in media coverage of ransomware attacks, health care providers can strategically address these areas of common interest and influence media publicity to their advantage.

The prevalence of responsibility media frames indicates that perceptions of continuity of service during a ransomware attack are important to manage, so further research is needed to understand the measures health care providers can take to address concerns about responsibility. For example, health care providers can demonstrate their organizational cyber resilience—the ability to return to full operational capacity after an unplanned outage—by communicating that the organization is taking responsibility for the patient experience and offer reassurance to patients and health care practitioners that their experiences have been heard and acknowledged. Organizational cyber resilience (business continuity or disaster recovery) is achieved through strong cybersecurity attack prevention, detection, and recovery measures [52]. As part of an organization’s disaster recovery or business continuity planning, health care organizations with strong, effective governance structures in place developed an incident response plan, which typically will include a communications or media strategy for cybersecurity attacks. For a health care provider, this will also include specific response strategies for managing impacts of a cybersecurity attack on their health care informatics systems technology. Knowing the prevalence of media frames in news publicity after a cybersecurity attack and the importance of clear and accessible communication could facilitate effective crisis management [53,54]; therefore, incorporating strategies for harnessing news media as a communications tool for crisis management could become an integral part of a health care provider’s incident response planning process. These contingency plans for responding to a cyberattack of a health care system should include communications that proactively address issues that are of priority concern for most people, which our findings indicate are the impacts to human components—the patients, the providers, staff, and the organization itself. Further studies are needed to validate these findings and measure the efficacy of influencing the use of media frames to reassure the public about the organization’s response to the human interest impacts of ransomware attacks on health care information systems.

Fifth, we found during our data screening process that approximately 14% (17/48) of full-length articles assessed for eligibility and excluded from this study appeared to promote a product or consulting service or spoke hypothetically about ransomware events among health care providers, specifically to events that had not yet occurred, in a phenomenon known as cyber doom [55]. This pattern is concerning and warrants further investigation to understand the implications of irresponsible news media coverage and its implications on public health and health care provider perceptions. Health care organizations may need to implement a concerted effort to reduce cyberdoom news articles that could potentially lead to misinformation regarding health care service providers.

### Limitations

This paper has several limitations pertaining to our interpretation of the results that can guide future research. First, the article selection process may have been influenced by selection bias due to multiple news articles referring to the same incident, as well as the inability to include any relevant news articles that were not stored in the academic news databases or were not returned using the systematic article search and filtering process. Only news articles that strictly followed the PRISMA processes were included in the analysis. Another latent source of potential bias is that many ransomware attacks may have gone unreported, such as in cases where the health care provider discreetly pays the ransom rather than risk adverse publicity or lawsuits surrounding adverse patient outcomes. In other words, the smaller sample we evaluated may represent only the tip of the iceberg. Therefore, further studies are needed to address these potential biases, such as conducting anonymous surveys to elucidate information gaps related to unreported events of ransomware attacks.

Second, our research design depended on the number of news article stories rather than the number of incidences of ransomware attacks. Although there may have been multiple articles reporting on the same incident, different journalists may have focused on different media framings and NIST security functions. Our framework accounted for the fact that the public may not see every article published on a specific story; on the other hand, the public may search for additional information from varied sources. In both scenarios, how an incident is reported may reflect different media framing and portrayals used for the same incident, which is the broader intent of our research study.

Third, insights into ransomware attacks should be interpreted with caution. Journalists and news outlets may not correctly use the correct or standardized vocabulary terms; for example, they may confuse a ransomware attack mechanism (eg, Trojan) with an intention (eg, ransom). Some of our characterizations that is, impacts and portrayals, are limited by the information available in each article, which may have obscured the accuracy of the terms used. We did not search for specific details, such as attack mechanisms, as general terms used for news reporting are usually vague and inconclusive. We attempted to be as consistent as possible given the source material but also careful not to mischaracterize what was reported based on our subjective understanding to reduce the risk of inferring information that was not reported. More studies are needed because of the subjective element in interpreting news media reports, especially as more articles on ransomware attacks become available.

### Future Research

Health care providers should consider novel strategies to reduce connections in the impacts pathway. Many health care providers continue to be caught off guard by ransomware attacks on their health care informatics systems. In developing contingency plans, considerations should be made for perception management of the impacts of these events on patient care and employee and staff well-being. This is particularly important for health care organizations that are already experiencing other problems such as burnout or understaffing [56], where other IT stressors [57,58] in addition to ransomware and denial of service attacks could be the tipping point in work satisfaction and retention measures. In one study, Zhao et al [59] conducted a survey of staff impacts from ransomware attacks on a trauma center by focusing on surgical residents whose adverse outcomes lasted for 2 months. The study found that resident physicians providing trauma patient care experienced significant stress, and resident physicians who were competent in digital processes had a difficult time adapting to manual processes, which caused attending surgeons to put forth additional effort to become effective teachers under those unusual circumstances. Such risk factors impact the adoption of new IT systems, as one study found that ransomware was a key security factor deterring IT adoption among doctors and nurses, because clinicians have experienced the interference of ransomware attacks in their daily work processes and their ability to provide quality patient care [60].

In addition, there is growing evidence from clinical practice and research that ransomware attacks impact patient morbidity and mortality. Ghafur et al [61] analyzed the negative outcomes of the National Health Service in Great Britain after the 2017 WannaCry ransomware attack. They found that although there were no statistically significant differences in mortality rates compared to the baseline, there was an economic impact of £5.9 million (US $7.7 million). They noted that more research is needed to analyze the adverse outcomes of ransomware attacks on patient care delivery and safety. However, Choi and Johnson [62], who conducted a statistical analysis of hospital data breaches (not including the impacts of denial of service or ransomware attacks), found that even data breaches with seemingly no connection to patient care were associated with a statistically significant difference in mortality when using a 30-day mortality rate measure. Furthermore, the authors found that the financial costs of mitigating a breach event may negatively impact patient care due to the diversion of resources. Dameff et al [63] studied 2 urban emergency departments and showed that the impact of significant operational disruption in the wake of a ransomware attack at one hospital resulted in increases in the patient census, number of patients who left without being seen, and the total length of stay, among other metrics at nontargeted hospitals regionally. The existing reports of serious impacts to patient quality of care and overall survival indicate there is an urgent need to minimize the negative impacts of ransomware attacks on patient outcomes. Further studies are needed to uncover innovative strategies for responding more effectively to ransomware attacks, such as the possibility of considering an isolated ransomware attack of one health care facility’s information system to be a regional disaster, which could trigger a coordinated response among neighboring facilities. Understanding how news media can be used to support crisis management during ransomware attacks on health care information systems could lead to coordinated efforts and community support rather than lawsuits and backlash, which in turn could lead to better outcomes for patients who are impacted by these events.

### Conclusions

Using a CQA, the aim of this study was to elucidate the function of news media frames and portrayals of the impacts of ransomware attacks on health informatic systems, employees, and patients in the context of the NIST CST 2.0 framework. We found that the core function of organizational response was significantly represented in news media portrayals of ransomware attacks. We identified indicators suggesting that health care providers should manage perceptions of ransomware attacks to ensure (1) a perception of continuity of health care service despite the unavailability of health informatics, (2) a perception of taking responsibility for the patient experience, and (3) acknowledgment of strain on health care practitioners and patients through a public declaration of support and gratitude. However, future studies are needed to guide the implementation and validate the efficacy of these potential strategies. We discovered from the media portrayals that there was a pattern in cascading impacts from a single point of failure, which indicates that cybersecurity management should address single points of failure to reduce cascading impacts, although future studies are needed to verify this finding.

## Appendix

Multimedia Appendix 1 PRISMA (Preferred Reporting Items for Systematic Reviews and Meta-Analyses) statement for reporting literature searches in systematic reviews.

Multimedia Appendix 2 Standards for reporting qualitative research.

Multimedia Appendix 3 Detailed extracted characteristics for each news story.

Multimedia Appendix 4 Notes related to the extraction of impacts.

Multimedia Appendix 5 The final media frames assigned to each article.

Multimedia Appendix 6 News articles with article ID merged in one file.

Multimedia Appendix 7 Media portrayals of health care informatics systems based on National Institute of Standards and Technologies (NIST) Cybersecurity Framework (CSF) 2.0. Core functions contain a detailed article ID coding crosswalk for each NIST CSF 2.0 core function.

## Abbreviations

CQA: collaborative qualitative analysis
CSF: Cybersecurity Framework
EHR: electronic health record
NIST: National Institute of Standards and Technology
PHI: personal health information
PRISMA: Preferred Reporting Items for Systematic Reviews and Meta-Analyses

## Data Availability

All data generated or analyzed during this study are included in this published article and its supplementary information files.
