# Supplementary material for: Media Framing and Portrayals of Ransomware Impacts on Informatics, Employees, and Patients: Systematic Media Literature Review
Source: J Med Internet Res. 2025 Apr 8;27:e59231. doi: 10.2196/59231 (PMC12015346; doi:10.2196/59231)
Supplement: Multimedia Appendix 3 [file jmir_v27i1e59231_app3.docx]

**Multimedia** **Appendix 3: News Story Characteristics**

| **Article ID** | **Article Title** | **Authors** | **Year** | **Healthcare Provider** | **Country** | **Attack Type** | **Impact Perspective** | **Outlet** |
| --- | --- | --- | --- | --- | --- | --- | --- | --- |
| #690 | AIIMS attack led to new SOP for breaches: Outgoing cyber chief | No Byline | 2023 | All India Institute of Medical Sciences | India | Ransomware | Patients & Researchers | Hindustan Times [New Delhi, India] |
| #57 | AIIMS Delhi server restored from 'cyberattack', all services continue manually | Dhar, Aniruddha | 2022 | All India Institute of Medical Sciences | India | Ransomware | Patients | Hindustan Times |
| #76 | AIIMS server outage being probed as 'cyber terrorism': Delhi Police | No Byline | 2022 | All India Institute of Medical Sciences | India | Ransomware | Patients & Employees | Hindustan Times |
| #16 | All of records erased, doctor's office closes after ransomware attack | Carlson, Joe | 2019 | Brookside ENT & Hearing Services | United States | Ransomware | Patients | Star Tribune [Minneapolis, MN] |
| #108 | Alvarado hospital fighting cyber attack | Sisson, Paul | 2016 | Alvarado Hospital Medical Center | United States | Ransomware | Patients | San Diego Union-Tribune, The [CA.] |
| #358 | BRIEF: Grand jury indicts guard accused of hacking computer system at Dallas clinic | Selk, Avi | 2009 | Carrell Clinic | United States | Ransomware | Patients & Employees | Dallas Morning News, The [TX] |
| #384 | BRIEF: Heritage Valley Health System dealing with 'cybersecurity incident' | Schmitt, Ben | 2017 | Heritage Valley Health System | United States | Other[cybersecurity incident] | Patients | Pittsburgh Tribune Review [PA.] |
| #543 | BRIEF: Heritage Valley lab draw services available as of Saturday morning | Sheleheda, Christina | 2017 | Heritage Valley Health System | United States | Ransomware | Patients | Beaver County Times, The [PA.] |
| #544 | BRIEF: Lab, diagnostic services still unavailable at Heritage Valley satellite locations | Stonesifer, Jared | 2017 | Heritage Valley Health System | United States | Ransomware | Patients | Beaver County Times, The [PA.] |
| #21 | British Hospitals Among Targets Of Global Ransomware Attack. | Langfitt, Frank | 2017 | National Health Service | United Kingdom | Ransomware | Patients & Employees | All Things Considered [NPR] |
| #456 | Chemotherapy patients sent home | Myers, Rebecca | 2017 | St. Bartholomew’s Hospital | United Kingdom | Ransomware | Patients | The Sunday Times |
| #91 | Cheyenne Regional payroll impacted by ransomware attack | Black, Hannah | 2022 | Cheyenne Regional Medical Center | United States | Ransomware | Employees | Wyoming Tribune-Eagle [Cheyenne, WY] |
| #14 | Crozer Health's computer system were offline Thursday morning | Brubaker, Harold | 2023 | Crozer Health | United States | Ransomware | Patients | Philadelphia Inquirer, The [PA] |
| #206 | Cyber attack hits Israeli hospitals | No Byline | 2017 | Israel Health System | Israel | Ransomware | Patients & Employees | Globes [Israel] |
| #150 | Cyberattack brings down Humber River Hospital computers. | Sarrouh, Maria; Ogilvie, Megan | 2021 | Humber River Hospital | Canada | Ransomware | Patients & Employees | Toronto Star [Canada] |
| #215 | Cyberattack concerns Humber MDs. | Ogilvie, Megan | 2021 | Humber River Hospital | Canada | Ransomware | Patients & Employees | Toronto Star [Canada] |
| #664 | Cyberattack hits major hospital in Spanish city of Barcelona | The Associated Press | 2023 | Hospital Clinic de Barcelona | Spain | Ransomware | Patients & Employees | AP Financial News |
| #661 | Cyberattack on top Indian hospital highlights security risk | Ghosal, Aniruddha | 2022 | All India Institute of Medical Sciences | India | Ransomware | Patients & Employees | AP Top News Package |
| #207 | England, Spain, Russia, other countries reeling from ransomware attack | Robbins, Gary | 2017 | National Health Service | United Kingdom | Ransomware | Patients & Employees | The San Diego Union-Tribune [CA.] |
| #269 | Hackers disrupt email, scheduling systems at Temple University Health System | Brubaker, Harold | 2019 | Temple University | United States | Other [no comment] | Patients & Employees | The Philadelphia Inquirer, [PA] |
| #37 | Hackers Strike Another Hospital System | Bergal, Jenni | 2021 | Memorial Health System | United States | Ransomware | Patients & Employees | Stateline.org [Washington, DC] |
| #179 | Heritage Valley continues to recover from cyberattack | Schmitt, Ben | 2017 | Heritage Valley Health System | United States | Ransomware | Patients | Pittsburgh Tribune Review [PA.] |
| #545 | Heritage Valley Health System's community offices closed after cyberattack | Schmitt, Ben | 2017 | Heritage Valley Health System | United States | Ransomware | Patients | Pittsburgh Tribune Review [PA.] |
| #493 | Heritage Valley still dealing with effects of Tuesday's cyber attack | Stonesifer, Jared | 2017 | Heritage Valley Health System | United States | Ransomware | Patients & Employees | The Beaver County Times, [PA.] |
| #649 | Hospital chain attack part of ongoing cybersecurity concerns | Foody, Kathleen; Kruesi, Kimberlee | 2022 | Common Spirit Health | United States | Ransomware | Patients & Employees | AP Top News Package |
| #659 | Hospital chain says 'IT security issue' disrupts operations | Associated Press | 2022 | Common Spirit Health | United States | Other [no description] | Patients | AP Regional State Report [Rhode Island] |
| #89 | Hospital computer hacks, like at ARH, becoming more common | Nuzum, Lydia | 2016 | Appalachian Regional Healthcare | United States | Other [not at liberty to provide] | Employees | The Charleston Gazette, [WV.] |
| #4 | Hospital Sisters Health System restores health record access | Wells, Valerie | 2023 | Hospital Sisters Health Systems | United States | Other [cybersecurity incident] | Patients | Herald & Review [Decatur, IL] |
| #9 | Hospital Sisters Health System still battling 'cybersecurity incident' | Petty, Allison | 2023 | Hospital Sisters Health Systems | United States | Other [cybersecurity incident] | Patients & Employees | Herald & Review [Decatur, IL] |
| #168 | In another case tonight, Comey`s FBI is leading the investigation of a hostage situation at a California hospital. | Pelley, Scott; Evans, Carter | 2016 | Hollywood Presbyterian Medical Center | United States | Ransomware | Patients | CBS Evening News with Katie Couric |
| #534 | IT shutdown at MedStar bogs down operations | Cox, John Woodrow | 2016 | Medstar | United States | Other [refused to characterize as ransomware] | Patients & Employees | Washington Post, The |
| #691 | Jury Selection Begins in Controversial Hospital Hacking Case | Dorstewitz, Michael | 2018 | Boston Childrens Hospital | United States | Other[hacking] | Patients & Employees | Newsmax.com |
| #692 | Los Angeles hospital attack concerns cybersecurity experts | Pritchard, Justin | 2016 | Hollywood Presbyterian Medical Center | United States | Ransomware | Employees | Associated Press State Wire: [CA.] |
| #15 | Los Angeles Hospital Pays Hackers To Regain Control Of Medical Records. | Siegler, Kirk | 2016 | Hollywood Presbyterian Medical Center | United States | Ransomware | Patients & Employees | All Things Considered [NPR] |
| #334 | 'Major disruption' as UK hospitals hit by cyber attack | No Byline | 2017 | National Health Service | United Kingdom | Ransomware | Patients | Al Jazeera [Qatar] |
| #652 | Major Florida hospital hit by possible ransomware attack | The Associated Press | 2023 | Tallahassee Memorial Healthcare | United States | Other [IT security issue] | Patients | AP Regional State Report [GA.] |
| #671 | New Ponemon Report Shows Ransomware Continues to Impact Patient Safety, According to Survey of Hospital IT/Security Leaders | Thompson, Justyne; Schneck, Joshua^[[1]](#footnote-1)^ | 2023 | Unidentified Hospitals | United States | Ransomware | Patients | Business Wire [English] |
| #686 | North Korean hackers now targeting hospitals and healthcare providers, US agencies warn | No Byline | 2022 | Unidentified Hospitals | United States | Ransomware | Patients | UPI Top World News |
| #61 | Ransomware attack delays lab results. | Omstead, Jordan | 2022 | Hospital for Sick Children | Canada | Ransomware | Patients & Employees | Toronto Star [Canada] |
| #129 | Ransomware attack hits Yuba City clinic | Vaughan, Monica | 2016 | Yuba-Center Medical Clinic | United States | Ransomware | Patients & Employees | Appeal-Democrat [Marysville, CA] |
| #38 | Ransomware attack launched on Salina Family Healthcare Center | Kozubowski, Jean | 2017 | Salina Family Healthcare Center | United States | Ransomware | Employees | The Salina Journal, [K.S.] |
| #346 | Ransomware attacks such as at South Bend's Allied Physicians are becoming common | Semmler, Ed | 2018 | Allied Physicians | United States | Ransomware | Patients | South Bend Tribune [IN] |
| #263 | Some Cheyenne Regional employees upset by handling of Kronos hack | Black, Hannah | 2022 | Cheyenne Regional Medical Center | United States | Ransomware | Employees | Wyoming Tribune-Eagle [Cheyenne, WY] |
| #688 | Surgeries delayed as hospital network hacked | Baxendale, Rachel | 2019 | Gippsland Health Alliance & South West Alliance | Australia | Ransomware | Patients & Employees | The Australian National, [Australia] |
| #203 | Today some patients in the Washington area were turned away after an attack on a hospital chain`s computer system. | Pelley, Scott; Van Cleave, Kris | 2016 | Medstar | United States | Other [malware] | Patients & Employees | CBS Evening News with Katie Couric |
| #331 | Virus prompts MedStar computer shutdown | Cox, John Woodrow; Turner, Karen; Zapotosky, Matt | 2016 | Medstar | United States | Other [virus] | Patients & Employees | The Washington Post |
| #447 | Virus shut down computer drive at Haley vets' hospital | Altman, Howard | 2015 | James A. Haley Veteran's Hospital | United States | Other [virus or ransomware trojan] | Employees | Tampa Tribune [FL.] |
| #55 | "WE HACKED THE HACKERS". | Strahan, Michael; Katersky, Aaron | 2023 | Unidentified hospital in Ohio | United States | Ransomware | Patients | Good Morning America [ABC] |

1. Authors not attributed in news database article but are attributed in publicly available online article [↑](#footnote-ref-1)
