# Supplementary material for: Media Framing and Portrayals of Ransomware Impacts on Informatics, Employees, and Patients: Systematic Media Literature Review
Source: J Med Internet Res. 2025 Apr 8;27:e59231. doi: 10.2196/59231 (PMC12015346; doi:10.2196/59231)
Supplement: Multimedia Appendix 4 [file jmir_v27i1e59231_app4.docx]

**Multimedia Appendix 4: News Story Impact Extraction Notes**

| **Article ID** | **Article Title** | **Impact Notes** |
| --- | --- | --- |
| #690 | AIIMS attack led to new SOP for breaches: Outgoing cyber chief | Wiped outpatient and research data from primary and backup servers. Significant implications for patient care and any ongoing medical research. |
| #57 | AIIMS Delhi server restored from 'cyberattack', all services continue manually | Even after the server was restored following the cyberattack, all hospitals and lab services were still running in manual mode. Manual mode is run when computers are down. This still allows health care providers to get results by paper and fax, but it is much slower than using a computer system. It also increases the chances of the workers making errors manually putting in results. The slightest error in results can change a patient's diagnosis and the way they are treated. Working in manual mode also puts more work and more strain on the healthcare workers. It is very time-consuming and can delay patient care. |
| #76 | AIIMS server outage being probed as 'cyber terrorism': Delhi Police | Registrations and appointments were halted, causing delayed results and patient care. Delayed patient care could have extreme consequences on patients in life/death situations. For example, a physician having the results of a patient’s troponin, a biomarker indicative of a heart attack within minutes vs. a few hours, could save the person's life. It was also stated that registration needed to be done manually, which leaves room for human error. This could result in inaccurate lab results and misdiagnoses. |
| #16 | All of records erased, doctor's office closes after ransomware attack | The first healthcare provider in the nation to shut down because of a ransomware attack. The attack deleted patient records, bills, and appointments. The office closed instead of paying the attackers because they weren't sure if paying them would fix the problem. All patient records and lab results were lost. A patient would have to get retested at another facility if they needed these results. This costs the patients more time and money to get retested. |
| #108 | Alvarado hospital fighting cyber attack | Impacts include two facilities, although HCA formally declined to specify the systems involved. Other sources indicate disruptions. The article mentions other HCAs experienced down email and impact on patient record databases. |
| #358 | BRIEF: Grand jury indicts guard accused of hacking computer system at Dallas clinic | Virus uploaded to patient records and air condition control systems could have significant implications for patient care and may even put hospital workers at physical risk. |
| #384 | BRIEF: Heritage Valley Health System dealing with 'cybersecurity incident' | No specifics were indicated; however, it was reported that the incident was widespread and affected the entire health system, including satellite and community locations. Operations were modified to ensure patient safety. |
| #543 | BRIEF: Heritage Valley lab draw services available as of Saturday morning | Lab services are down for a few days at multiple facilities. |
| #544 | BRIEF: Lab, diagnostic services still unavailable at Heritage Valley satellite locations | Lab and diagnostic services are down at multiple locations. Unable to communicate when service would be back up. |
| #21 | British Hospitals Among Targets Of Global Ransomware Attack. | A ransomware attack struck over 30 facilities, causing physicians and hospital staff to return to working with pen and paper. This slowed the processes of day-to-day hospital activities, causing a delay in patient care and potential errors from manually recording data. |
| #456 | Chemotherapy patients sent home | Patient stress and trauma due to the ordeal of chemotherapy but also chemotherapy being canceled or delayed, which is a direct impact on patient care and well-being. |
| #91 | Cheyenne Regional payroll impacted by ransomware attack | 2000 employees were impacted when timekeeping and payroll processing systems caused employees to be overpaid or underpaid. Employees overpaid and had to pay money back. The impacted HR system was down for several months; other details indicate it was 5 billing cycles. The HCSA thanked employees for their patience and acknowledged it was a difficult situation. Cybersecurity insurance did not cover this, and ultimately, an expert noted it was the HCSP’s job to ensure payments were made correctly. |
| #14 | Crozer Health's computer systems were offline Thursday morning | Employees reported to the media that for the second time in some time they were the victims of a ransomware attack. This is evidence that staff were likely under distress. No other details or information provided including details on patient care. Noted that spokesperson did not immediately respond to messages. |
| #206 | Cyberattack hits Israeli hospitals | Claims there were no interruptions or disruptions to work at the hospital. |
| #150 | Cyberattack brings down Humber River Hospital computers. | A cyberattack shut down a hospital’s IT system, causing the staff to be unable to access electronic patient records, including diagnostic test results. This caused a delay in patient care and added stress on the hospital staff. |
| #215 | Cyberattack concerns Humber MDs. | A cyberattack at Humber Hospital concerned doctors about patient safety. Concerned for patient safety, the physicians asked the administration to temporarily close the emergency department until the IT systems were restored. Patients cannot be treated properly without medical and diagnostic test results, putting the patients in compromising and dangerous conditions, especially in an emergent situation. |
| #664 | Cyberattack hits major hospital in Spanish city of Barcelona | Canceled 150 non-urgent operations and 3,000 patient checkups, shut down computers at the Lab, ER, and Pharmacy and several affiliated healthcare service organizations. Work moved to paper. New urgent cases were diverted to other hospitals in the city. Access to patient records and communication between units was cut off. |
| #661 | Cyberattack on top Indian hospital highlights security risk | Leading hospital in India crippled by cyberattack for almost 2 weeks. Although the hospital was in the process of digitizing records, records were inaccessible because of the cyberattack. This caused delays in patient care and more stress added to the hospital staff. |
| #207 | England, Spain, Russia, other countries reeling from ransomware attack | NHS patients moved, and some were delayed healthcare services. This may have direct harm to the patients. |
| #269 | Hackers disrupt email, scheduling systems at Temple University Health System | Email and scheduling systems were down at a Philadelphia hospital because of the computer system being hacked. Having the scheduling system down caused a delay in patient care because of the inability to make appointments. |
| #37 | Hackers Strike Another Hospital System | A cyberattack on this hospital caused the cancellation of urgent surgeries and the diversion of ambulances to other emergency departments. The diversion to other emergency departments causes overworked hospital staff and a delay in patient care. Overworked employees are also susceptible to making mistakes in charting, which could cause errors in patient care. |
| #179 | Heritage Valley continues to recover from cyberattack | Notice that almost all facilities were up and running, which gives the impression that the incident was bigger than originally reported. Lab and diagnostic imaging services are still unavailable at some locations at the time of the article. |
| #545 | Heritage Valley Health System's community offices closed after cyberattack | The organization announced the attack with an emphasis that no information had been accessed, however investigation was ongoing. Furthermore, did not specifically address the question on whether or not patient care was impacted indicating some concern as the answer was not emphatically no. |
| #493 | Heritage Valley still dealing with effects of Tuesday's cyber attack | Patients are left with anxiety, shock, and discomfort as a result of cyberattacks. Paper charting generated additional labor in the form of an assistant. No access to medical records meant no access to patients’ medication lists. Lab & diagnostics were unavailable. The healthcare service provider wouldn't say whether appointments and procedures were canceled. Patients canceled but did not reschedule as there was no access to computer systems. |
| #649 | Hospital chain attack part of ongoing cybersecurity concerns | A cyberattack on a hospital computer system caused delayed patient treatment and diverted ambulances. Diverted ambulances resulted in more work on hospital staff at the other facility and also delayed patient treatment. Overworked staff increases the likelihood of errors/misdiagnoses. While the computer system was down, the healthcare workers had to work from paper charts. Manually entering data from paper charts once computer systems are recovered could introduce data entry errors. Having incorrect data entered for test results could make a patient's diagnosis go from normal to critical and vice versa. |
| #659 | Hospital chain says 'IT security issue' disrupts operations | The article states IT security issues disrupted operations in several states. Because of this issue, ambulances were diverted from one emergency department to another. Diverted patients cause overcrowding at the facility the patients were diverted to, causing overworked employees, delayed patient care, and possibly errors/mistakes in patient care because of the overworked employees. |
| #89 | Hospital computer hacks, like at ARH, becoming more common | Unable to access patient records, email, or other automated systems. |
| #4 | Hospital Sisters Health System restores health record access | Massive outage affecting 8 hospitals and multiple community clinic partners. The impact is on the communications systems. The outage appears to be with messages being sent. |
| #9 | Hospital Sisters Health System still battling 'cybersecurity incident' | Communication systems hospital and clinic operations are affected. The hospital has a plan in place that it executed. The disruption required the postponement of elective surgeries, outpatient surgeries, and blood drives. |
| #168 | In another case tonight, Comey`s FBI is leading the investigation of a hostage situation at a California hospital. | The hospital has had to resort to pen and paper and fax machines for communications. Disruption had been ongoing for two weeks at the time of the article. |
| #534 | IT shutdown at MedStar bogs down operations | IT shut down at a MedStar hospital facility, causing the staff to have to turn patients away or to treat them without important computer records. The hospital staff had to use paper charts, which are less comprehensive than digital charts. This causes decreased care for the patient. |
| #691 | Jury Selection Begins in Controversial Hospital Hacking Case | Hacktivist attacks caused interference with online service portals for patients, providers, and physicians. |
| #692 | Los Angeles hospital attack concerns cybersecurity experts | Publicly disclosed they paid a ransom but indicated patient care was not compromised. |
| #15 | Los Angeles Hospital Pays Hackers To Regain Control Of Medical Records. | LA hospital paid 40 bitcoins (approx. $17,000.00) to hackers who shut down its computer system for days. The hospital had to shut down all computers and revert to writing medical records by hand. This causes room for error, delays in patient care, and added stress to hospital staff. |
| #334 | 'Major disruption' as UK hospitals hit by cyber attack | It was stated in the article that appointments were canceled, and patients were diverted to other hospitals, causing a delay in patient care. This could be devastating in a life-or-death situation for a patient. Surgeries were canceled, causing not only a delay in patient care but patients might also have had employment affected because of rescheduling. When the ambulances were diverted to a different hospital, this caused more work for the other hospital staff. Overworked staff could introduce mistakes in documentation, treatment, etc., which all affect the patient. |
| #652 | Major Florida hospital hit by possible ransomware attack | The organization diverted emergency room patients, canceled surgeries, and rescheduled non-emergency patient appointments. It was unclear when systems would be back online. |
| #671 | New Ponemon Report Shows Ransomware Continues to Impact Patient Safety, According to Survey of Hospital IT/Security Leaders | All hospitals rely heavily on technology. This article stated ransomware attacks have an adverse effect on patient mortality rates and patient care. According to the CDC, 70% of all patient diagnoses are based on lab results. Not being able to view lab results prevents physicians from being able to do their job and causes a delay in patient care. |
| #686 | North Korean hackers now targeting hospitals and healthcare providers, US agencies warn | No specific healthcare service provider was mentioned, but a warning that servers responsible for health records, diagnostic, and imaging services could be compromised. |
| #61 | Ransomware attack delays lab results. | Delays in lab results had an impact on patient care. |
| #129 | Ransomware attack hits Yuba City clinic | Ransomware limited staff access to data and had an impact on operations. The impact on operations was posited as a potentially better outcome than disclosure or breach, which is interesting to note. |
| #38 | Ransomware attack launched on Salina Family Healthcare Center | A cyberattack on a hospital computer system shut down the computer system briefly, but patient care was continued and not affected. The health center's clients were notified about the attack and were offered a free year of credit report protection and monitoring in case their personal information was compromised. |
| #346 | Ransomware attacks such as at South Bend's Allied Physicians are becoming common | 40 doctor practice indicated that its network was shut down but that there was no impact on patient care. |
| #263 | Some Cheyenne Regional employees upset by handling of Kronos hack | Employees impacted by ransomware attack of 3rd party HR management and payroll system were so upset they contacted the media outlining actions the company took internally that exacerbated the impacts and delays. The attack impacted payroll such that employees were overpaid or underpaid. There was frustration in getting to a resolution, most notably due to short staffing and the organization not using additional labor to help speed things up. |
| #688 | Surgeries delayed as hospital network hacked | The hospital had to revert to manual contact and booking systems. Financial management systems, patient records, booking, and management systems were shut down and not available. |
| #203 | Today some patients in the Washington area were turned away after an attack on a hospital chain`s computer system. | Cyberattack at 10 facilities. Health providers had to shut down computer systems. The hospital has been slow, and appointments have been canceled or pushed back. Doctors were asked to use paper and pen to record notes. |
| #331 | Virus prompts MedStar computer shutdown | Ransomware attacks caused hospital staff to use seldom-used paper charts and records. Paper charts and records are not as extensive as digital charts and records, so the patients weren't receiving complete health care during this time. 70% of patient diagnosis is based on data from lab results. Not having all of these results in a timely manner creates a delay in patient care. |
| #447 | Virus shut down computer drive at Haley vets' hospital | A shared computer drive was shut down for 5 days because of a virus. This did not allow hospital employees to effectively do their jobs, causing a delay in patient care. No access to results causes a delay in patient diagnosis and treatment. |
| #55 | We Hacked The Hackers | A hospital in Ohio was unable to accept patients due to an attack. No other details were provided regarding the attack. |
