# Supplementary material for: Media Framing and Portrayals of Ransomware Impacts on Informatics, Employees, and Patients: Systematic Media Literature Review
Source: J Med Internet Res. 2025 Apr 8;27:e59231. doi: 10.2196/59231 (PMC12015346; doi:10.2196/59231)
Supplement: Multimedia Appendix 5 [file jmir_v27i1e59231_app5.docx]

**Multimedia Appendix 5: News Story Media Frame Coding**

| **Article ID** | **Title** | **Frame Code Consensus** | **Frame Code Notes (Summary)** |
| --- | --- | --- | --- |
| #690 | AIIMS attack led to new SOP for breaches: Outgoing cyber chief | 5 | The article discusses the government's response and the development of a new framework to handle cybersecurity issues, highlighting a responsibility and a structural approach towards managing and possibly preventing such incidents in the future. |
| #57 | AIIMS Delhi server restored from 'cyberattack', all services continue manually | 5 | This article discusses the actions taken by various authorities, such as AIIMS, Delhi Police, and CERT-IN, in response to a cyberattack, emphasizing the efforts to restore, secure, and investigate the breach. They took responsibility for the attack by cleaning the servers and deploying more workers to the site. |
| #76 | AIIMS server outage being probed as 'cyber terrorism': Delhi Police | 2 | The article touches on how the ransomware attack impacts services for patients, which brings a human and emotional angle to the story. |
| #16 | All of records erased, doctor's office closes after ransomware attack | 3 | The excerpt highlights the financial impact of a ransomware attack, leading to a healthcare provider's shutdown, and reflects broader economic risks for similar businesses. |
| #108 | Alvarado hospital fighting cyber attack | 5 | This excerpt presents a case where hospitals, including Alvarado Hospital Medical Center, are addressing malware disruptions. The narrative highlights the proactive and reactive measures taken by healthcare facilities and their IT teams, emphasizing their responsibility in promptly responding to cyber threats, ensuring operational continuity, and safeguarding patient records. The mention of not yielding to ransom demands further reinforces the stance of responsibility and resilience against cyber extortion. |
| #358 | BRIEF: Grand jury indicts guard accused of hacking computer system at Dallas clinic | 5 | The responsibility is clearly attributed to the individual (the guard) who breached his duty by engaging in cybercrime and the legal system holding him accountable, underscoring societal norms about the protection of sensitive information and the consequences of violating them. The planned legal repercussions reinforce the notion of responsibility and accountability in cybersecurity breaches. |
| #384 | BRIEF: Heritage Valley Health System dealing with 'cybersecurity incident' | 5 | The event was posited as a cybersecurity incident with very few details shared; however, the organization immediately sought to offer a solution for downtime procedures and made operational adjustments. |
| #543 | BRIEF: Heritage Valley lab draw services available as of Saturday morning | 5 | The organization emphasized that it took responsibility for the solution to the restoration and resumption of services as well as taking initiative in the investigation of the cyberattack with a strong focus on operational continuity. |
| #544 | BRIEF: Lab, diagnostic services still unavailable at Heritage Valley satellite locations | 5 | The article presents an issue or problem in such a way as to attribute responsibility for its cause or solution to either the government or to an individual or group. Strong focus on operational disruptions and their impact on patient care, including the day-to-day consequences of the attack on patient care, such as losses in efficiency and customer trust and operational paralysis as an inability to communicate to stakeholders when service would be back up. |
| #21 | British Hospitals Among Targets Of Global Ransomware Attack. | 2,3 | Emphasized both financial consequences and Real portrayals of the impacts of returning to pen and paper when ransomware attack struck 30 facilities. Delved into the impact of ransomware attacks on various organizations, emphasizing the financial aspects of the ransom and the cost implications of the attacks. |
| #456 | Chemotherapy patients sent home | 2 | This incident underscores that while the immediate effects of such attacks are technological, the ripple effects can profoundly impact human lives, particularly when they disrupt healthcare services, emphasizing the vulnerability and potential risks to patient welfare in cybersecurity breaches. This frame encourages an empathetic understanding of the cyberattack, spotlighting the personal stories and struggles that result from such incidents. |
| #91 | Cheyenne Regional payroll impacted by ransomware attack | 3 | Economic Consequences Frame (3). The ransomware attack on the payroll software used by Cheyenne Regional Medical Center and its health system affects over 2,000 employees and their payments, thereby having significant economic implications. |
| #14 | Crozer Health's computer system were offline Thursday morning | 1 | It exposes the conflict between Crozer Health and unknown entities that may have compromised its computer systems, leaving them offline. It also reflects on a past instance of a malware attack, including an attack on the newspaper, giving an impression of an ongoing conflict. |
| #206 | Cyber attack hits Israeli hospitals | 5 | Emphasis on the actions taken by the Ministry of Health and the National Cyber Defense Authority in responding to the attack and ensuring that the hospitals could continue to function which highlights the responsibility and capability of these entities in managing and mitigating the cyberattack. The hospital managed the narrative that not 8 but only 2 hospitals were impacted. |
| #150 | Cyberattack brings down Humber River Hospital computers. | 2 | Strong focus on the staff unable to access electronic patient records, including diagnostic test results, causing a delay in patient care and added stress on the hospital staff. Functionality is shut down. |
| #215 | Cyberattack concerns Humber MDs. | 2,5 | The article highlights significant disruptions to the hospital's operational ability and patient care. Organizations took responsibility by redirecting resources to manage the crises and maintain operations. Discusses a heartfelt appeal letter made by physicians who are seeing 200 patients a day in the midst of an attack. The physicians are taking responsibility for ensuring patient care and safety but are using emotional appeals to ask that the hospital shut down the emergency department until IT systems are back up and running. |
| #664 | Cyberattack hits major hospital in Spanish city of Barcelona | 2,5 | This piece underscores the immediate human impact of a ransomware attack on Barcelona's main hospital. By detailing the cancellation of 150 non-urgent operations and up to 3,000 patient checkups, the narrative evokes concern and empathy for those directly affected. The report emphasizes the tangible disruptions in critical healthcare services and the ensuing uncertainty for both the patients awaiting medical care and the hospital staff scrambling to maintain operational continuity. The focus remains on the human element of the crisis rather than the technical details of the cyberattack.; They took responsibility by identifying the attackers and working to restore the computer system. |
| #661 | Cyberattack on top Indian hospital highlights security risk | 2 | This article provides narratives of people affected by a cyberattack on the All India Institute of Medical Sciences. |
| #207 | England, Spain, Russia, other countries reeling from ransomware attack | 5 | Discusses the WannaCry attack with the context that the NHS hospitals were not the only victims attributed it was a cross-sector impact of the attack. The article focused on how common the attack was, how it worked, and other victims. |
| #269 | Hackers disrupt email, scheduling systems at Temple University Health System | 1 | It describes a cybersecurity incident that affected Temple University Health System in late August, disrupting email, patient scheduling, and other functions. This conflict scenario involves the health system and unidentified hackers. |
| #37 | Hackers Strike Another Hospital System | 2 | There was significant emphasis on the disruption of surgeries and the impact on patients and hospital operations due to the ransomware attack. This approach places a human face on the issue, demonstrating the real-world implications of cyberattacks on healthcare services and individuals needing care. |
| #179 | Heritage Valley continues to recover from cyberattack |  | An announcement made declaring the hospital chain open. However, specific healthcare service providers were mentioned as being open including a pediatrician, OB/GYN, a medical group, two hospitals, and numerous unspecified walk-in clinics. This indicates a very massive, scaled attack occurred. The article mentions however that lab and diagnostic imaging were still not available at the time of the article at most locations. |
| #545 | Heritage Valley Health System's community offices closed after cyberattack. | 5 | The organization identified the attackers and focused on implementing more protective measures for their systems. |
| #493 | Heritage Valley still dealing with effects of Tuesday's cyber attack | 2 | This piece brings a personal perspective to a global cyber-attack, focusing on the human experience and emotional response. It describes Jamie Davis's return to a pre-digital era medical visit due to the hack, evoking nostalgia and concern, particularly her anxiety over a potential personal data breach. The narrative encapsulates the human side of cybersecurity issues, emphasizing individual stories and emotions amidst a larger crisis. |
| #649 | Hospital chain attack part of ongoing cybersecurity concerns | 2 | The consequences, as outlined by Brett Callow, involve direct impacts on patient care, including life-threatening delays in treatment. |
| #659 | Hospital chain says 'IT security issue' disrupts operations | 2 | Impacts on patient care were discussed, including the diversion of ambulances from the emergency department records system was taken down. Also, the article discussed the implications if health records were actually accessed, it would need to be reported; this would elicit an emotion in the reader. |
| #89 | Hospital computer hacks, like at ARH, becoming more common | 5 | The hospital’s response to the cyberattack appeared to be solutions oriented. It is noted that the hospital reported the attack and also provided details on safeguards, work around the restoration of services, and even when it would provide updates. |
| #4 | Hospital Sisters Health System restores health record access | 5 | The organization remained focused on the restoration of services. |
| #9 | Hospital Sisters Health System still battling 'cybersecurity incident' | 5 | The organization remained focused on restoring service, working diligently, working with law enforcement, and working with the FBI to resolve the situation. |
| #168 | In another case tonight, Comey`s FBI is leading the investigation of a hostage situation at a California hospital. | 2,3 | This frame is highlighted by the economic impact and consequences of the cyberattack on the hospital, especially given that the hospital paid an undisclosed amount as ransom to regain control of its systems. The situation emphasizes the financial implications and considerations due to the cyberattack. The title of the article humanizes the data and information systems held hostage in the ransom attack. |
| #534 | IT shutdown at MedStar bogs down operations | 2 | The excerpt discusses the challenges faced by nurses and potential risks to patients. |
| #691 | Jury Selection Begins in Controversial Hospital Hacking Case | 1 | The involvement of various parties, including the hacktivist group, the hospital, and the legal representatives, accentuates the conflict aspect. |
| #692 | Los Angeles hospital attack concerns cybersecurity experts | 3 | This passage highlights the financial implications of the ransomware attack, focusing on the $17,000 paid by the hospital to regain control of its systems. The excerpt also underscores the hospital's vulnerability to such attacks due to outdated cybersecurity measures, implying significant costs associated with potential future attacks and the need for investments in stronger cybersecurity defenses. |
| #15 | Los Angeles Hospital Pays Hackers To Regain Control Of Medical Records. | 3 | The article leads with and primarily focuses on the $17,000 the hospital has paid for the ransomware attack. |
| #334 | 'Major disruption' as UK hospitals hit by cyber attack | 2 | The significant impact on hospitals and, subsequently, on patients and medical services brings a strong human and emotional angle to the situation described in the article. |
| #652 | Major Florida hospital hit by possible ransomware attack | 2 | The news focuses on the direct human consequences of a potential ransomware attack on a major Florida hospital. By highlighting the disruptions in critical medical services, like the diversion of emergency room patients and the cancellation of surgeries, the piece draws attention to the immediate, tangible impacts on individuals' lives. It evokes concern and empathy by underscoring the uncertainty and potential risks patients face rather than delving into the technical aspects of the cyberattack. |
| #671 | New Ponemon Report Shows Ransomware Continues to Impact Patient Safety, According to Survey of Hospital IT/Security Leaders | 2,5 | "With patient safety in jeopardy and 'asymmetric warfare' no longer hyperbole to describe the situation, this report highlights the continued threats while introducing new approaches to creating rigorous, robust, and continuous cyber programs that protect patients." |
| #686 | North Korean hackers now targeting hospitals and healthcare providers, US agencies warn | 1,5 | Emphasizes the conflict between North Korean hackers and US healthcare organizations, with a clear disagreement and reproach between parties. The article states North Korean hackers are responsible for the cyberattacks. |
| #61 | Ransomware attack delays lab results. | 2 | This excerpt is best encapsulated by the "Human Interest Frame." It brings to light the real-world consequences of the cyberattack on patients and their families, emphasizing the emotional stress caused by delayed lab and imaging results and the potential for extended wait times, all within the context of a healthcare setting that caters to children's well-being. |
| #129 | Ransomware attack hits Yuba City clinic | 2 | By likening the ransomware to a "chain lock around your house," the article personalizes the attack, making it relatable to the everyday reader and accentuating the feeling of invasion and helplessness. Rather than delving into technical details, it emphasizes the operational chaos, the inconvenience to patients due to rescheduled appointments, and the clinic's efforts to navigate the situation. The human-interest angle is further amplified by not discussing whether the ransom was paid, instead highlighting the emotional and operational impact on the clinic and its patients. |
| #38 | Ransomware attack launched on Salina Family Healthcare Center | 2,3,5 | This article focuses on the actions taken by the healthcare center's staff in response to the ransomware attack, emphasizing their decision-making and responsibilities. The discussion about notifying patients, hiring a forensic investigator, and offering credit report protection highlights the center's accountability in managing the aftermath. Additionally, public and patient concerns highlighted depict a human element to the attack, and the financial implications of this attack are explicitly discussed as strongly as the responsibility and the human impact. |
| #346 | Ransomware attacks such as at South Bend's Allied Physicians are becoming common | 5 | The article emphasizes the responsibility of both individuals and organizations in preventing ransomware attacks, highlighting how negligence, such as clicking on unsolicited email links or having outdated software, can lead to breaches. It underscores the proactive measures that can be taken to safeguard systems, implying a shared responsibility between employees' cautious behavior and institutional security protocols to mitigate cyber threats. The piece also touches on the consequences of failing these responsibilities, including the potential loss of critical data even after a ransom is paid. |
| #263 | Some Cheyenne Regional employees upset by handling of Kronos hack | 2,3 | Employees contacted the media, indicating a human element; there are also direct financial implications from the attack as some employees were overpaid and expected to pay back the money or underpaid and had to wait to be made whole. |
| #688 | Surgeries delayed as hospital network hacked | 2 | Discussion of the impact on surgeries and hospital services, which directly affects patients and brings a human face to the issue of the cyberattack. |
| #203 | Today some patients in the Washington area were turned away after an attack on a hospital chain`s computer system. | 2 | The excerpt interviews actual patients and gives a face to the impact of the cyberattack. |
| #331 | Virus prompts MedStar computer shutdown | 3,5 | The excerpt emphasizes MedStar Health's immediate response actions and their duty to manage the crisis, highlights the broad impact on operations and patients, indicating the organization's obligation to maintain service continuity, and points to the necessity for preventive measures and preparedness strategies, suggesting a broader responsibility in safeguarding against cyber threats; The article also discusses the financial implications of the attack. |
| #447 | Virus shut down computer drive at Haley vets' hospital | 2,5 | The article focuses on how the Trojan virus impacted the operations of the James A. Haley Veterans' Hospital and potentially affected thousands of workers despite an assurance that patient care was not altered. The human element is underlined by discussing how staff interacts with the system and the implications of the shutdown. |
| #55 | We Hacked The Hackers | 1,5 | The report highlights the US government's retaliatory action against the notorious hacking group Hive, emphasizing the "fight-back" narrative. By infiltrating the group's operations and seizing their servers, federal agents showcase their proactive stance against cyber threats. The piece underscores the tug-of-war between hackers and federal agencies, framing it as a battle where the US authorities have won a significant round against cyber adversaries; this indicates a responsibility for the solution. |
