# Supplementary material for: Media Framing and Portrayals of Ransomware Impacts on Informatics, Employees, and Patients: Systematic Media Literature Review
Source: J Med Internet Res. 2025 Apr 8;27:e59231. doi: 10.2196/59231 (PMC12015346; doi:10.2196/59231)
Supplement: Multimedia Appendix 6 [file jmir_v27i1e59231_app6.pdf]

Record: 1

Ransomware attack hits Yuba City clinic By: Vaughan, Monica. Appeal-Democrat (Marysville, CA). 09/13/2016. (*AN: 2W61282767946*)

Database: Newspaper Source

Ransomware attack hits Yuba City clinic

~~~~~~~~

Monica Vaughan

Sept. 13--A Yuba City medical clinic's computer system was attacked by overseas hackers with what federal agencies are calling a fast-growing cybercrime: Ransomware.

Ransomware is a digital attack designed to block access to portions of a computer system until the user pays a ransom. Since the beginning of 2016, more than 4,000 daily attacks have targeted home computer users, businesses and government networks, according to a special U.S. government interagency report.

Yuba-Sutter Medical Clinic was hit with such an attack on Aug. 3, according to a press release issued Sunday by attorney Robert Rymek.

He said the malware did not compromise patient information, rather it temporarily limited staff access to data.

"Picture coming home and finding someone's put a chain lock around your house," Rymek said. "It prevents you from getting in there. Its not that (the hackers) are accessing what's inside."

Although no digital information was lost, the attack impacted operations. The clinic was temporarily limited from obtaining access to certain data, which resulted in delays and the need to reschedule appointments, Rymek said.

The clinic was able to quickly regain access within a day or two, he said. He did not comment on whether the ransom was paid.

There was some indication the attackers were based in Russia or China, and the incident was reported to federal law enforcement authorities for investigation, he said.

"Its the hot security topic of 2016," Rymek said.

Some attacks result in an intimidating message, such as: "Your computer was used to visit websites with illegal content. To unlock your computer, you must pay a $100 fine."

Attackers sometimes gain access to computer networks by tricking a user to share a password or open an email attachment with a virus, a government report says. Then, attackers often request payment of the ransom with bitcoin, a form of digital currency.

The best defense is proactive training, as well as the use of spam filters, firewalls that block known malicious IP addresses, and the use of anti-virus and anti-malware programs that conduct regular scans.

CONTACT reporter Monica Vaughan at 749-4783 and on Twitter @MonicaLVaughan.
~~~~~~~~

___ (c)2016 the Appeal-Democrat (Marysville, Calif.) Visit the Appeal-Democrat (Marysville, Calif.) at www.appeal-democrat.com Distributed by Tribune Content Agency, LLC.

Copyright of **Appeal-Democrat (Marysville, CA)** is the property of Appeal-Democrat (Marysville, CA). The copyright in an individual article may be maintained by the author in certain cases. Content may not be copied or emailed to multiple sites or posted to a listserv without the copyright holder's express written permission. However, users may print, download, or email articles for individual use.

Source: Appeal-Democrat (Marysville, CA), Sep 13, 2016
Item: 2W61282767946

Title: Virus prompts MedStar computer shutdown By: John Woodrow Cox, Karen Turner, Matt Zapotosky, Washington Post, The, 03/29/2016

Database: Newspaper Source

This content may contain URLs/links that would redirect you to a non-EBSCO site. EBSCO does not endorse the accuracy or accessibility of these sites, nor of the content therein.

Listen

'ransomware' may have been usedHospital giant unsure how long system will be offline

A virus infected the computer network of MedStar Health early Monday morning, forcing the Washington health-care behemoth to shut down its email and vast records database and raising additional concerns about the security of hospitals nationwide.

The FBI is investigating the breach, which comes just weeks after similar cyberattacks on at least three medical institutions, in California and Kentucky. Still, MedStar officials said they had found "no evidence that information has been stolen."

"MedStar acted quickly with a decision to take down all system interfaces to prevent the virus from spreading throughout the organization," spokeswoman Ann Nickels said in a statement. "We are working with our IT and cyber-security partners to fully assess and address the situation. Currently, all of our clinical facilities remain open and functioning."

But the infection could have a considerable impact on the $5 billion health-care provider, which operates 10 hospitals and more than 250 outpatient facilities in the Washington region. It serves hundreds of thousands of patients and employs more than 30,000 people.

Without access to sophisticated online systems, hospital staff have had to revert to seldom-used paper charts and records.

"Everything will be slowed down tremendously," said Stephen Frum, a labor representative for National Nurses United who has worked closely with MedStar for 15 years. "It's huge."

Appointments and surgeries will be delayed, he said, explaining that it will take longer for lab results to come back, for patients to receive tests and for medications to be ordered.

Neither MedStar nor the FBI has said how long it expects the systems to remain offline.

"Even the lowest-level staff can't communicate with anyone. You can't schedule patients. You can't access records. You can't do anything," said one employee who asked that her name not be used because she was not authorized to speak about the incident.

The woman said she spoke to two other employees who saw a pop-up message on their computer screens stating that they had been infected by a virus and asking for ransom in "some kind of Internet currency." She had not seen the pop-up message herself.

Though the nature of the MedStar infection remains unclear, Nickels said Monday that she had "not been told that it's a ransom situation."

"Ransomware" - a virus that holds systems hostage until victims pay for a key to regain access - has been deployed at least three times against hospitals this year.

In one case last month, a hospital in Los Angeles paid hackers $17,000 in bitcoins, an Internet currency, to free its system. Forbes identified that strain of ransomware as "Locky" - a reference to the virtual lock the virus places on data.

"The quickest and most efficient way to restore our systems and administrative functions was to pay the ransom and obtain the decryption key," the hospital's president, Allen Stefanek, said in a Feb. 17 statement. "In the best interest of restoring normal operations, we did this."

Two weeks ago, a Kentucky facility announced it was in an "internal state of emergency" after a similar hack, according to the site Krebs on Security, which reported that the hackers in that case asked for about $1,600 in bitcoins.

Medical facilities are vulnerable to these attacks in part because they don't properly train their employees on how to avoid being hacked, according to Sinan Eren, who has worked in cybersecurity for government and health-care organizations for two decades.

"It's not like the financial-services industry, where they train employees how to spot suspicious emails," said Eren, general manager at Avast Mobile Enterprise.

Also, many hospital computer systems are outdated, bulky and in dire need of upgrades or newer software, he said. But such institutions often don't have - or don't want to spend - the money to make sweeping changes.

"There's a lack of budget, a lack of talent to handle these issues," Eren said. "Sometimes the human capital might not be there. All these things are an incremental cost to their systems. Therefore, they kind of push the can down the road to deal with technical updates later."

Special Agent Chris Stangl, a section chief at the FBI's cyber division, said in a recent interview that ransomware attacks are becoming increasingly prevalent as more and more victims pay up. In a nine-month period in 2014, the FBI investigated 1,838 complaints of such attacks, which cost those targeted more than $23.7 million. In 2015, agents investigated 2,453 complaints, costing targets $24.1 million.

Stangl said the hackers, most of them from Eastern Europe, have increasingly targeted businesses, which are often able to pay more than individuals to unlock data. The hackers "scan the Internet for companies that post their contact information," then send them email phishing attacks. Unsuspecting employees, Stangl said, are asked to click on what seem to be innocuous links or attachments - perhaps something as simple as a .PDF purporting to be a customer complaint - and before they know it, their computers are infected.

"In the beginning days of ransomware, the target was primarily individuals, and it was unsophisticated, just very small amounts that people would pay," Stangl said. "It's kind of moved, as the actors have become more sophisticated, to small- to medium-sized businesses."

Stangl said the crime is financially motivated, and the hackers make demands that put their victims in a difficult spot. They target critical data - such as patient records - then ask for a ransom low enough that a business or individual will consider paying it.

john.cox@washpost.com

matt zapotosky@washpost.com

Source: Washington Post, The, 03/29/2016
Item: wapo.480f7d66-f515-11e5-a3ce-f06b5ba21f33

Record: 1

Virus shut down computer drive at Haley vets' hospital By: Altman, Howard. Tampa Tribune (FL). 09/16/2015. (*AN: 2W63052848007*)

Database: Points of View Reference Center

Virus shut down computer drive at Haley vets' hospital

~~~~~~~~

Howard Altman

Sept. 16--TAMPA -- A computer drive shared by thousands of workers at the James A. Haley Veterans' Hospital was shut down five days until this morning after the discovery of a computer virus known as a Trojan.

No information was extracted from the system and there was no change in patient care, hospital officials say.

"There was no breach of data and patient care operations have not been affected," said hospital spokeswoman Karen Collins. Internet security personnel "are completing the final checks on the remediation process before opening up the S drive for normal usage," Collins said Tuesday afternoon.

In an email at 10 a.m. today, Collins said the shared drive is back up.

The drive, where hospital personnel can store documents and other information to be shared, can be used by anyone with access to the hospital's system. Collins said that more than 4,000 people work at Haley and have access.

The the system's security programs detected what is known as a ransomware Trojan. This is a virus that enters a computer system when a user is tricked into clicking on a link in an email or on a website that gives the virus access. Users are often asked to pay money to restore the compromised documents.

"Some documents on the shared drive were infected and that set off an alarm," Collins said. "This affects all employees with computer access, more than 4,000 people."

Regional internet security staff "is still reviewing any cost associated with this event and there doesn't seem to be any major work disruptions at this time," Collins said.

Items that were on the drive were being backed up, Collins said, but a local cybercrime website said that may not recover all the lost data.

"Once these files are encrypted, the only way to get them back is to restore a recent backup or pay the ransom," according to the website of KnowBe4, a Clearwater-based cyber security firm.

The website sites other sources to say that about a third of companies fail to test their backups and and that three-fourths of backups failed to restore.

Collins said the hospital has not paid any ransom. Citing security concerns, she declined to talk about how long files are backed up in the system.
~~~~~~~~

Stu Sjouwerman, KnowBe4 chief executive officer, said ransomware Trojan viruses "are the next wave in cybercrime."

They are often the work of "Eastern European cyber mafia," Sjouwerman said. "This is a crime that brings in tens of millions of dollars."

Ransomware Trojan attacks often leave systems vulnerable even after the malware has been removed, he said, but Collins said whoever sent the virus to the Haley system has no further access.

haltman@tampatrib.com

(813) 259-7629

Twitter: @haltman

___ (c)2015 the Tampa Tribune (Tampa, Fla.) Visit the Tampa Tribune (Tampa, Fla.) at www.tampatrib.com Distributed by Tribune Content Agency, LLC.

Copyright of **Tampa Tribune (FL)** is the property of Tampa Tribune (FL). The copyright in an individual article may be maintained by the author in certain cases. Content may not be copied or emailed to multiple sites or posted to a listserv without the copyright holder's express written permission. However, users may print, download, or email articles for individual use.

Source: Tampa Tribune (FL), Sep 16, 2015
Item: 2W63052848007

Disclaimer: This is a machine generated PDF of selected content from our products. This functionality is provided solely for your convenience and is in no way intended to replace original scanned PDF. Neither Cengage Learning nor its licensors make any representations or warranties with respect to the machine generated PDF. The PDF is automatically generated "AS IS" and "AS AVAILABLE" and are not retained in our systems. CENGAGE LEARNING AND ITS LICENSORS SPECIFICALLY DISCLAIM ANY AND ALL EXPRESS OR IMPLIED WARRANTIES, INCLUDING WITHOUT LIMITATION, ANY WARRANTIES FOR AVAILABILITY, ACCURACY, TIMELINESS, COMPLETENESS, NON-INFRINGEMENT, MERCHANTABILITY OR FITNESS FOR A PARTICULAR PURPOSE. Your use of the machine generated PDF is subject to all use restrictions contained in The Cengage Learning Subscription and License Agreement and/or the Gale General OneFile Terms and Conditions and by using the machine generated PDF functionality you agree to forgo any and all claims against Cengage Learning or its licensors for your use of the machine generated PDF functionality and any output derived therefrom.

Today some patients in the Washington area were turned away after an attack on a hospital chain`s computer system

Date: 2016
From: CBS Evening News
Publisher: CQ-Roll Call, Inc.
Document Type: Broadcast transcript
Length: 439 words

Full Text:
SCOTT PELLEY: Today some patients in the Washington area were turned away after an attack on a hospital chain`s computer system. Kris Van Cleave is looking into this.

(Begin VT)

KRIS VAN CLEAVE: The crippling cyberattack hit ten MedStar facilities throughout the Washington region and prompted the health provider to shut down its computer systems to prevent further intrusions. Courtney Martin (ph) works at Georgetown Hospital.

COURTNEY MARTIN: Yeah, every thing`s shut down here. So there`s paper. Every thing`s by hand today.

KRIS VAN CLEAVE: While the hospitals have stayed open, it`s been slow, and some patients reported having their appointments canceled.

BARBARA GRAY: I`m like, okay, but you can still write things down.

KRIS VAN CLEAVE: When the doctor`s office tried to cancel on Barbara Gray`s husband, she said no way.

BARBARA GRAY: I just don`t think it`s to the point that you can`t see your doctor because before electronics we wrote things down, write it down.

KRIS VAN CLEAVE: MedStar says significant progress is being made on restoring its systems. So far the hospital is calling the virus malware but a spokeswoman would not deny the possibility it was so-called "ransomware," a malicious program allowing hackers to hold a computer system hostage for a price. Last month a California hospital paid seventeen thousand dollars in ransom to regain access to its medical records.

DR. JOHN HALAMKA (ph): That`s like negotiating with terrorists.

KRIS VAN CLEAVE: Doctor John Halamka is the chief technology officer at Beth Israel Deaconess health systems in Boston. Last year he took us inside one of the hospital groups secure data centers.

DR. JOHN HALAMKA: Many hospitals have not invested in security so they`re just more vulnerable. But now that we have an example of a hospital willing to pay, we are going to see more ransomware than ever.

(End VT)

KRIS VAN CLEAVE: Halamka says even if it isn`t ransomware, hackers see the value in medical records that are rich in personal information like Social Security numbers. Scott, so far MedStar says there`s no reason to believe patient or employee data have been compromised.

SCOTT PELLEY: Kris Van Cleave in the Washington newsroom. Kris, thank you.

In a moment, the miraculous performance that earned Patty Duke an Oscar.

(ANNOUNCEMENTS)

END

[Copy: Content and programming Copyright MMXVI CBS Broadcasting Inc. ALL RIGHTS RESERVED. Copyright 2016 CQ-Roll Call, Inc. All materials herein are protected by United States copyright law and may not be reproduced, distributed, transmitted, displayed, published or broadcast without the prior written permission of CQ-Roll Call. You may not alter or remove any trademark, copyright or other notice from copies of the content.]

Copyright: COPYRIGHT 2016 CQ-Roll Call, Inc.
http://www.thenewsroom.com/
Source Citation (MLA 9th Edition)
"Today some patients in the Washington area were turned away after an attack on a hospital chain`s computer system." *CBS Evening News*, 29 Mar. 2016. *Gale General OneFile*, link.gale.com/apps/doc/A447910001/ITOF?u=naal_aub&sid=ebsco&xid=3adf4e7f. Accessed 17 Sept. 2023.
Gale Document Number: GALE|A447910001

Title: Hackers disrupt email, scheduling systems at Temple University Health System By: Brubaker, Harold, Philadelphia Inquirer, The (PA), Sep 12, 2019

Database: Newspaper Source

Hackers disrupt email, scheduling systems at Temple University Health System

This content may contain URLs/links that would redirect you to a non-EBSCO site. EBSCO does not endorse the accuracy or accessibility of these sites, nor of the content therein.

Listen

~~~~~~~~

Harold Brubaker

Sept. 12-- Sep. 12--Hackers disrupted Temple University Health System's computer systems in late August, disabling the North Philadelphia health care provider's email, patient scheduling and other functions.

The problem started late on Aug. 28, health system spokesman Jeremy Walter said in a statement. "Beginning in the early hours of Friday morning, August 30, the impact was being reversed, and affected systems are back on-line," he said.

The Pennsylvania Department of Health declined to comment.

A spokesperson for the Pennsylvania Association of Staff Nurses & Allied Professions, which represents 2,300 Temple University Hospital employees, said they saw little impact from the attack on Temple's systems.

KYW radio reported the cyber attack on Sept. 2.
~~~~~~~~

"It just goes to show, even at places that have really superb cyber-security there's vulnerability. I think it really does point out how vigilant places need to be," Larry Kaiser, the health system's chief executive, said Tuesday in an interview about his departure from that position at the end of this month.

Asked if it was a ransomware attack, Kaiser said he couldn't comment.

Ransomware software locks up files on computers or servers with the goal of making them inaccessible. School districts and local state governments have become popular targets of such attacks. At least 70 local and state governments have been affected by ransomware this year, according to researchers at Barracuda, an IT security company. Municipalities in Florida and Texas, and Baltimore, where all affected.

___ (c)2019 The Philadelphia Inquirer Visit The Philadelphia Inquirer at www.inquirer.com Distributed by Tribune Content Agency, LLC.

Copyright of **Philadelphia Inquirer, The (PA)** is the property of Philadelphia Inquirer, The (PA). The copyright in an individual article may be maintained by the author in certain cases. Content may not be copied or emailed to multiple sites or posted to a listserv without the copyright holder's express written permission. However, users may print, download, or email articles for individual use. **Source:** Philadelphia Inquirer, The (PA), Sep 12, 2019
Item: 2W62225828010

Disclaimer: This is a machine generated PDF of selected content from our products. This functionality is provided solely for your convenience and is in no way intended to replace original scanned PDF. Neither Cengage Learning nor its licensors make any representations or warranties with respect to the machine generated PDF. The PDF is automatically generated "AS IS" and "AS AVAILABLE" and are not retained in our systems. CENGAGE LEARNING AND ITS LICENSORS SPECIFICALLY DISCLAIM ANY AND ALL EXPRESS OR IMPLIED WARRANTIES, INCLUDING WITHOUT LIMITATION, ANY WARRANTIES FOR AVAILABILITY, ACCURACY, TIMELINESS, COMPLETENESS, NON-INFRINGEMENT, MERCHANTABILITY OR FITNESS FOR A PARTICULAR PURPOSE. Your use of the machine generated PDF is subject to all use restrictions contained in The Cengage Learning Subscription and License Agreement and/or the Gale In Context: Opposing Viewpoints Terms and Conditions and by using the machine generated PDF functionality you agree to forgo any and all claims against Cengage Learning or its licensors for your use of the machine generated PDF functionality and any output derived therefrom.

Some Cheyenne Regional employees upset by handling of Kronos hack.

Date: Apr. 10, 2022
From: Wyoming Tribune-Eagle (Cheyenne, WY)
Publisher: Adams Publishing Group
Document Type: Article
Length: 1,754 words
Content Level: (Level 4)
Lexile Measure: 1250L

Full Text:
Byline: Hannah Black

Apr. 10CHEYENNE Some employees say Cheyenne Regional Medical Center and its overall health system did not adequately handle an outage that affected its timekeeping and payroll software.

The Wyoming Tribune Eagle reported March 26 that more than 2,000 CRMC employees were affected when its human resources management system, Kronos Private Cloud, went down Dec. 11 after a larger-scale ransomware attack.

The WTE spoke with three Cheyenne Regional employees who reached out after that story was published. They did not want to be identified because they said they fear retaliation from the hospital.

These employees were frustrated largely because of what they felt was management not being readily available while the system was down. In some cases, they questioned the accuracy of their pay, mainly because it was unclear to them how their paychecks were calculated during the outage.

They claimed that the payroll department was understaffed, and said they felt devalued by certain responses to their frustrations. These workers said they are paid by the hour.

While Kronos was down, the health system's payroll department manually processed paychecks over five pay cycles, Chief Human Resources Officer Joanna Vilos said previously. Vilos says about 55% of Cheyenne Regional employees were overpaid, while about 45% were underpaid.

"One of our top priorities was to ensure paychecks were issued timely on each and every payday, which we successfully accomplished," Vilos said in a Thursday statement to the WTE.

Kronos says the core functionality of its payroll software, including "time, scheduling and HR/payroll capabilities," was restored by Jan. 22. Cheyenne Regional says the software became fully functional in early March, and Vilos said that when the hospital could access the payroll system, it "immediately began reconciling all employees' paychecks."

Those who were underpaid received the money they were owed in their March 17 paycheck, an employee said.

Vilos says no personal information was compromised, thanks to the health system's "robust set of policies and practices against cyberattacks."

Payroll department

Employees said just two people staffed the health system's payroll department during the Kronos outage.

In her Thursday statement, Vilos confirmed the payroll team was staffed with two full-time employees, saying it was "supplemented with ongoing assistance from human resources, accounting and administrative staff."

"I feel like a lot of the over- (and) underpayment stuff could have been prevented if maybe they would have contracted in some outside help, instead of dumping this onto employees," said a staffer. "We were getting messages of 'Don't call payroll, they're very

busy. They'll set up a room in one of the conference rooms on such and such day from such and such hours. If you have questions, do it then.'"

Since Kronos came back online, the employee said, they've been asked to schedule meetings with the payroll department.

Staff said there were several email communications from hospital leadership, but that some aspects were difficult to understand. Employees perceived the messaging as not transparent.

"This is a widespread concern within the organization, and there are a lot of unhappy employees," one hospital worker said. "There's a lot of inconsistencies with what they're saying, and I think that's one of the primary reasons people are upset is that we're not getting straightforward answers."

When one employee scheduled an in-person meeting with a payroll department employee, the hospital worker said she wanted to find out more from payroll about how they'd determined paycheck amounts. According to this hospital worker, payroll did not have information about individual employees' insurance deductibles, or even pay scale, during at least part of the outage.

"I told (the payroll department employee) that I really wanted to double check and make sure that everything matches up, everything aligns, before I pay anything back, and she said, 'I will make a note to the VP that you are refusing to pay back the money you owe,'" the hospital worker recalled. "I told her that is not what I said, and later on, she retracted that statement, saying she had never said that. So I did, I got a little riled up with her because I felt that was inappropriate and she was setting me up."

The employee said she was told that if she did not pay back the money she was told she owed, the amount would be sent to collections.

Inconsistencies

In communications from the health system, an employee said, hospital workers were told to "double check" their paychecks to ensure they were paid correctly.

"But the way they do the pay system, it almost makes it impossible for a person to figure it out. And, for the most part, my paychecks looked accurate. So they just kept saying, you know, just double check, double check," she said.

But when she sent an email to an employee in the payroll department outlining concerns about discrepancies, the payroll employee "basically kind of wrote it off and said: 'Just be assured that your paychecks are correct,'" she said.

To this hospital worker, the payroll employee's comment contradicted what had been said earlier about double-checking pay amounts.

The worker said she was underpaid in her first two paychecks, then overpaid in subsequent February paychecks. This was confusing, because during the periods she was overpaid, she was told Kronos was back up and running.

According to Vilos: "As Kronos came back online, we then had to reconcile each pay period in chronological sequence during the timeframe that Kronos was not operational. The timeframe to complete the reconciliations crossed over an additional two pay periods. Upon completion of December's payroll reconciliation, we were then able to process and produce (IRS) W-2 tax forms for all employees.

"For many employees, as we estimated payroll based off a previous pay period, the pay, deductions and taxes were a direct reflection of the baseline pay period. In instances where we estimated an increase or bonus payment, the IRS standard tax rate of 22% was applied. In a week's time, after all reconciliations were completed, all underpayments were made."

'Mishandled'

Employees said they were given several options to pay back the money: pay it in a lump sum with check or cash; have the amount taken out of one paycheck; have it taken out over five paychecks; or cash out up to 160 hours of paid time off, and be unable to cash out any more this year.

Vilos said Thursday that "Employees who were overpaid had options to repay directly or through payroll deduction, either over a single pay period or over multiple pay periods, entirely their choice. For employees with hardship or unique challenges, we evaluated each one individually and provided additional options to better meet their unique circumstances."

One employee said she could tell she was being overpaid, so she set the money aside. She'd taken extra shifts in the weeks leading up to the Kronos outage during a spike in COVID-19 hospitalizations. This employee said it was her understanding that at least some of the paychecks during the Kronos outage were based a previous paycheck, meaning her pay during the outage was higher than normal.

She decided to pay the amount back all at once.

"When you have to write a check for (thousands of dollars), even though you know that it was overpayment money, it still kind of takes your breath away," the employee said.

Another said that, although she's been told she only owes a few hundred dollars, she's still trying to parse out the accuracy of her

pay.

Employees said they were instructed to keep manual timesheets to give to their supervisors during the outage. It was unclear whether these timesheets were used to calculate employee pay, as employees also said they were told their pay over the next five cycles was based on the amount in their Dec. 10 paycheck.

Vilos said CRMC "used a recent, previous pay period as the baseline for the five pay periods that Kronos was not fully operational, and estimates were made off of that baseline pay period."

Some of the employees said that the money they may owe isn't the main problem.

"The point is that they mishandled this situation," one employee said. "They didn't get the help that they desired or needed. (A payroll department employee) said that she made the choice, along with her supervisors, to not hire any extra employees or outside help to do payroll. So it was just two (payroll) employees for the 2,200 (total Cheyenne Regional) employees."

This employee said she's lost some of her confidence in management following this incident.

All the employees who spoke to the WTE said they were aware of situations where hourly hospital workers were drastically over- or underpaid.

One person said she and colleagues had been discussing the possibility of contacting a labor organization, though they weren't clear on exactly who to go to or where to start. She said they just wanted someone "impartial" to investigate how things were handled.

"This has been a difficult and challenging situation that we, and thousands of other Kronos clients, had to work through," Vilos wrote. "It has been difficult on our payroll, accounting and HR staff, as well as every single employee. We regret any inconvenience or frustration this has caused. Now we are moving forward as our system has been restored, historical accuracy adjudicated and normal payroll functionality resumed."

A different perspective

After the WTE contacted Cheyenne Regional with specific questions regarding these employee complaints, a hospital spokesperson said she had reached out to department managers. The spokesperson said she believed there were "many employees who may have a different point of view" than those who said they'd had negative experiences.

One additional employee then contacted the WTE. She also asked to remain anonymous.

This hospital worker, who is also hourly, said the payroll disruption didn't have much of an effect on her.

"I always know how many hours I work every day. So, for me, it wasn't a big deal," she said.

The employee said the collective feeling within her small department was one of empathy toward payroll employees, understanding that they had a daunting, complex task on their hands.

"So, I think for me personally and the people around me," she said, "it was just like, 'Oh, gosh, those poor people are going to be overwhelmed."

Hannah Black is the Wyoming Tribune Eagle's criminal justice reporter. She can be reached at hblack@wyomingnews.com or 307-633-3128. Follow her on Twitter at @hannahcblack.

___

(c)2022 Wyoming Tribune-Eagle (Cheyenne, Wyo.)

Visit Wyoming Tribune-Eagle (Cheyenne, Wyo.) at www.wyomingnews.com

Distributed by Tribune Content Agency, LLC.

Copyright: COPYRIGHT 2022 Adams Publishing Group
https://adamspg.com/
Source Citation (MLA 9th Edition)
"Some Cheyenne Regional employees upset by handling of Kronos hack." *Wyoming Tribune-Eagle* [Cheyenne, WY], 10 Apr. 2022, p. NA. *Gale In Context: Opposing Viewpoints*, link.gale.com/apps/doc/A700052976/OVIC?u=naal_aub&sid=ebsco&xid=3e363658. Accessed 16 Sept. 2023.
Gale Document Number: GALE|A700052976

Record: 1

Ransomware attack launched on Salina Family Healthcare Center By: Kozubowski, Jean. Salina Journal, The (KS). 08/26/2017. (*AN: 2W61702446569*)

Database: Points of View Reference Center

Ransomware attack launched on Salina Family Healthcare Center

~~~~~~~~

Jean Kozubowski

Aug. 26--Deborah Demel, chief financial officer of Salina Family Healthcare Center, went to work on June 18, a Sunday, turned on her computer and saw a ransomware message, indicating that someone had taken over the center's computer system and wanted money to return the data.

She turned off the computer, called police and called the center's information technology team.

According to the report filed that day with the Salina Police Department, 15 of the clinic's servers and 33 of 154 desk computers were affected. Because there seemed to be no monetary or information loss, the department did not follow up with an investigation, Capt. Paul Forrester said.

"We didn't find any evidence that anyone's personal information was accessed," said Dr. Rob Freelove, the health clinic's CEO.

However, officials also couldn't prove that information hadn't been accessed. "We thought we should be cautious," Freelove said.

The center's servers and computers were taken offline and scrubbed, backups were installed and the network was rebuilt.

"We were able to continue providing patient care almost immediately," Freelove said, in both the medical and dental clinics.

Freelove hired a forensic investigator to see if the source of the attack could be traced.

But according to center officials, it wasn't until about two months later that health center clients and the media were notified of the attack.

Letters were mailed to 70,000 medical and dental clinic patients and a press release was sent to media dated Aug. 16, informing of the attack and offering clients a free year of credit report protection.

Didn't do enough

Terry McLaughlin, of Salina, isn't sure the clinic is doing enough. And he's concerned about the length of time that's elapsed since the attack.

"A year's worth (of credit protection) is not adequate," he said. "Smart crooks would use the information immediately or hold on to it for a year or more."
~~~~~~~~

Potentially, he said, the information could have been used for two months or more without clients putting a fraud alert on their accounts.

And while he did get a letter, some people still haven't been notified, McLaughlin said. His wife, for example, had been told she would get a letter with a redemption code for identity protection, but it hasn't arrived.

Freelove said the center wasn't trying to hide anything. Officials were talking to their insurance company and to forensic investigators to see how serious the problem might be.

"We're looking out for the patients," he said.

Records not purged

McLaughlin is also unhappy that Salina Family Healthcare still has his information on file.

"My wife and I haven't been there for 13 years. My information should have been purged," McLaughlin said.

"The only thing they didn't get is my birth certificate."

And McLaughlin thinks it might be a breach of privacy to send a communication from a health center to an incorrect address.

That happened more than it should have, Freelove said.

He said some of the addresses on health center records are out-of-date. Freeelove said the clinic is required to keep files for 10 years and, in the case of children, 10 years past the age of majority, or age 18. If the clinic saw a 6-year-old, it would keep the child's chart until the child was 28, a total of 22 years. The address the clinic had for a 6-year-old is not likely to be the same as that person's address at the age of 28.

Letters went out to the best addresses that could be found for every patient who has visited since the clinic's opening.

But there's a further problem, Freelove said. The clinic hired a processing center to help with the letters, and it sent some to the wrong address. He did not specify where the incorrect addresses came from.

Freelove said anyone who received a letter from the health center that's addressed to someone else should mark it "Return to sender" and give it to a mail carrier or the post office.

He suggested that people who receive letters contact AllClearID for a year's worth of free identity protection services.

___ (c)2017 The Salina Journal (Salina, Kan.) Visit The Salina Journal (Salina, Kan.) at www.saljournal.com Distributed by Tribune Content Agency, LLC.

Copyright of **Salina Journal, The (KS)** is the property of Salina Journal, The (KS). The copyright in an individual article may be maintained by the author in certain cases. Content may not be copied or emailed to multiple sites or posted to a listserv without the copyright holder's express written permission. However, users may print, download, or email articles for individual use.

Source: Salina Journal, The (KS), Aug 26, 2017
Item: 2W61702446569

Title: Ransomware attack delays lab results. By: Jordan Omstead The Canadian Press, Toronto Star (Canada), 12/23/2022

Database: Newspaper Source

Ransomware attack delays lab results.

This content may contain URLs/links that would redirect you to a non-EBSCO site. EBSCO does not endorse the accuracy or accessibility of these sites, nor of the content therein.

Listen

A ransomware attack has delayed lab and imaging results at the Hospital for Sick Children and could lead to longer wait times, the hospital said Thursday, noting that some of its systems could be offline for weeks.

The hospital said it was unable to provide details about the nature of the attack, calling it an "active and ongoing incident."

There is no evidence to date that personal information had been affected, the hospital said.

"While we can confirm this is a ransomware attack, SickKids has been preparing for attacks of this nature, and mobilized quickly to mitigate potential impacts to the continuity of care," the hospital said in a written statement Thursday.

SickKids reported Monday night that a "cybersecurity incident" prompted the hospital to call a Code Grey - the hospital code for system failure - on Sunday at 9:30 p.m.

In its first update since then, the hospital said it would probably be a "matter of weeks" before all systems are working as normal.

"Clinical teams are currently experiencing delays with retrieving lab and imaging results, which may cause longer wait times for patients and families," the hospital said.

The ransomware attack comes as Canada's largest pediatric hospital has had to cancel some surgeries and redeploy staff in recent weeks to deal with a surge of sick kids with respiratory infections.

The Canadian Press

Copyright of **Toronto Star (Canada)** is the property of Toronto Star. The copyright in an individual article may be maintained by the author in certain cases. Content may not be copied or emailed to multiple sites or posted to a listserv without the copyright holder's express written permission. However, users may print, download, or email articles for individual use. **Source:** Toronto Star (Canada), 12/23/2022
Item: 6FPTS2022122366573092

Disclaimer: This is a machine generated PDF of selected content from our products. This functionality is provided solely for your convenience and is in no way intended to replace original scanned PDF. Neither Cengage Learning nor its licensors make any representations or warranties with respect to the machine generated PDF. The PDF is automatically generated "AS IS" and "AS AVAILABLE" and are not retained in our systems. CENGAGE LEARNING AND ITS LICENSORS SPECIFICALLY DISCLAIM ANY AND ALL EXPRESS OR IMPLIED WARRANTIES, INCLUDING WITHOUT LIMITATION, ANY WARRANTIES FOR AVAILABILITY, ACCURACY, TIMELINESS, COMPLETENESS, NON-INFRINGEMENT, MERCHANTABILITY OR FITNESS FOR A PARTICULAR PURPOSE. Your use of the machine generated PDF is subject to all use restrictions contained in The Cengage Learning Subscription and License Agreement and/or the Gale General OneFile Terms and Conditions and by using the machine generated PDF functionality you agree to forgo any and all claims against Cengage Learning or its licensors for your use of the machine generated PDF functionality and any output derived therefrom.

New Ponemon Report Shows Ransomware Continues to Impact Patient Safety, According to Survey of Hospital IT/Security Leaders.

Date: Jan. 18, 2023
From: Business Wire
Publisher: Business Wire, Inc.
Document Type: Article
Length: 853 words

Full Text:
Concurring with 2021 Landmark Study, Updated Report Shows Ransomware Continues to Have Adverse Impact on Patient Care, According to 579 Survey Respondents

BOSTON -- Ponemon Institute, the preeminent research center dedicated to privacy, data protection, and information security policy, surveyed 579 IT and IT security professionals at healthcare delivery organizations (HDOs) to understand how ransomware continues to impact patient care, and to determine the value of cybersecurity benchmarking to reduce cyber threats such as ransomware. The independent research report, titled *The Impact of Ransomware on Patient Safety and the Value of Cybersecurity Benchmarking,* published in January 2023 from a survey conducted in Q4 2022, was commissioned by Censinet, the leading provider of healthcare risk management solutions.

This 2023 report provides an update to the industry's first study on the impact of ransomware on patient safety, titled *The Impact of Ransomware on Healthcare During COVID-19 and Beyond*, published in September 2021 and also commissioned by Censinet. That seminal 2021 study was the first to demonstrate a qualitative correlation between ransomware and adverse impacts to patient care, including increased mortality rates. In this updated report, over half of respondents indicated that one or more ransomware attacks experienced by their organization resulted in a disruption to patient care. While the most prevalent impact identified was an increase in patients transferred or diverted to other facilities, over one-in-five respondents indicated that ransomware attacks had an adverse impact on patient mortality rates - nearly the same response rate as in the 2021 study. However, significantly more respondents this year indicated that ransomware attacks increased complications from medical procedures - up to 45 percent of respondents compared to 36 percent in 2021.

"Our findings indicate that Hospital IT/Security personnel continue to believe ransomware has a broad and adverse impact on patient care," said Dr. Larry Ponemon, Chairman and Founder of the Ponemon Institute. "With ransomware growing exponentially and most organizations under constant threat, this report also explores how peer benchmarking improves an HDO's cybersecurity program effectiveness, including its decision-making, hiring, and resource allocation."

The study also explored the importance of cyber programs and initiatives such as peer benchmarking and third-party vendor risk management for determining optimal investment levels and resource allocation required to reduce the risk of a ransomware attack and other cyber threats. The report found that:

* Benchmarking is very valuable in demonstrating cybersecurity program effectiveness, including cybersecurity framework coverage and compliance.

* Benchmarking is important to making the business case for hiring cyber staff and helps guide tool and technology purchasing for the cybersecurity program.

* Benchmarking is important when establishing cybersecurity program goals and enables better, more data-driven decision-making

* Benchmarking is helpful in responding to, and recovering from, ransomware attacks according to a majority of respondents.

"The findings in this year's Ponemon report are, unfortunately, not surprising as ransomware continues to shut down hospital operations and disrupt care at an alarming rate," said Ed Gaudet, CEO and Founder of Censinet. "With patient safety in jeopardy and 'asymmetric warfare' no longer hyperbole to describe the situation, this report highlights the continued threats while introducing new

approaches to creating rigorous, robust, and continuous cyber programs that protect patients."

Ponemon Institute and Censinet will present the details of the independent research report in an upcoming webinar, "The Impact of Ransomware on Patient Safety and the Value of Cybersecurity Benchmarking." It will be presented live on January 24 at 12:00 PM ET and features Dr. Larry Ponemon and Ed Gaudet, both leading advocates and experts in the healthcare information security industry. **Register here for the webinar any time before Jan 24 at 12pm ET.**

To receive a copy of the research report, *The Impact of Ransomware on Patient Safety and the Value of Cybersecurity Benchmarking*, or to learn more about the impact of ransomware on patient care and the value of cybersecurity peer benchmarking, please visit https://www.censinet.com/impact-of-ransomware-on-patient-safety-and-value-of-cybersecurity-benchmarking

About Ponemon Institute

Ponemon Institute is dedicated to independent research and education that advances responsible information and privacy management practices within business and government. Our mission is to conduct high-quality, empirical studies on critical issues affecting the management and security of sensitive information about people and organizations. We uphold strict data confidentiality, privacy, and ethical research standards. We do not collect any personally identifiable information from individuals (or company identifiable information in our business research). Furthermore, we have strict quality standards to ensure that subjects are not asked extraneous, irrelevant, or improper questions. Learn more at ponemon.org

About Censinet

Censinet[R], based in Boston, MA, takes the risk out of healthcare with Censinet RiskOps, the industry's first and only cloud-based risk exchange of healthcare organizations working together to manage and mitigate cyber risk. Purpose-built for healthcare, Censinet RiskOps(TM) delivers total automation across all third party and enterprise risk management workflows and best practices. Censinet transforms cyber risk management by leveraging network scale and efficiencies, providing actionable insight, and improving overall operational effectiveness while eliminating risks to patient safety, data, and care delivery. Censinet is an American Hospital Association (AHA) Preferred Cybersecurity Provider. Find out more about Censinet and its RiskOps platform at censinet.com.

View source version on businesswire.com: https://www.businesswire.com/news/home/20230118005592/en/

Copyright: COPYRIGHT 2023 Business Wire, Inc.
http://www.businesswire.com
Source Citation (MLA 9th Edition)
"New Ponemon Report Shows Ransomware Continues to Impact Patient Safety, According to Survey of Hospital IT/Security Leaders." *Business Wire*, 18 Jan. 2023, p. NA. *Gale General OneFile*, link.gale.com/apps/doc/A733614215/ITOF?u=naal_aub&sid=ebsco&xid=d63feb90. Accessed 16 Sept. 2023.
Gale Document Number: GALE|A733614215

Record: 1

IT shutdown at MedStar bogs down operations By: John Woodrow Cox. Washington Post, The. 03/30/2016. Abstract: Patients, workers report care gaps after possible 'ransomware' attack MedStar Health patients were being turned away or treated without important computer records Tuesday as the health-care giant worked to restore online systems crippled by a virus. [ABSTRACT FROM PUBLISHER] (*AN: wapo.252626ae-f5bc-11e5-a3ce-f06b5ba21f33*)

Database: Newspaper Source

Patients, workers report care gaps after possible 'ransomware' attack

MedStar Health patients were being turned away or treated without important computer records Tuesday as the health-care giant worked to restore online systems crippled by a virus.

By Tuesday evening, MedStar staff could read - but not update - thousands of patient records in its central database, though other systems remained dark, a spokeswoman said.

MedStar officials have refused to characterize the attack as "ransomware," a virus used to hold systems hostage until victims pay for a key to regain access. But a number of employees reported seeing a pop-up message on their computer screens seeking payment in bitcoins, an Internet currency. One woman who works at MedStar Southern Maryland Hospital Center sent The Washington Post an image of the ransom note, which demanded that the $5 billion health-care provider pay 45 bitcoins - equivalent to about $19,000 - in exchange for the digital key that would release the data.

"You just have 10 days to send us the Bitcoin," the note read, "after 10 days we will remove your private key and it's impossible to recover your files."

The cyberattack, which is being investigated by the FBI, forced MedStar's 10 hospitals and more than 250 outpatient centers to shut down their computers and email on Monday. The health-care system employs more than 30,000 people and treats hundreds of thousands of patients in the Washington region.

Spokeswoman Ann Nickels said that its facilities, which stretch from Arlington to Baltimore, have operated safely throughout the crisis.

But two nurses said the cyberattack created a chaotic environment in at least one MedStar location, and a doctor at another facility said it had created a "patient safety issue."

At MedStar Washington Hospital Center, one nurse who worked overnight described the situation as difficult. Without access to email and computer systems, the medical staff fell back on seldom-used paper records that had to be faxed or hand-delivered. But this nurse and another told The Post that the paper charts are far less comprehensive than those kept in digital form. They can be missing vital pieces of patient information: complete medical histories, every drug prescribed, allergies to medicine and treatment plans.

Without the computer systems, they explained, the health-care facilities were operating without a number of essential safeguards meant to prevent mistakes.

"Those are all in place to prevent human error," the doctor said, "and you lose all that when you lose the computer system."

Nickels denied that the cyberattack's impact was that dramatic.

"There's absolutely no indication of that from clinical leaders who are reporting in several times a day," she said. "It's stressful, but I think we all know what we need to do."

MedStar's chief medical officer, Stephen R.T. Evans, said in a statement Tuesday afternoon that "the quality and safety of our patients remains our highest priority, which has not waned throughout this experience."

The nurse, however, said the challenges were serious.

For example, because lab results were taking so much longer to process, the nurse continued to give one patient a powerful antibiotic - with a number of potentially serious side effects - that should have been discontinued.

"The medication," the nurse said, "should have been stopped eight hours earlier."

The doctor echoed that concern. Speedy lab results, he said, are crucial in determining the best way to treat infections and other ailments. He also criticized MedStar's preparation for the attack and how it communicated with employees afterward.

An emergency room nurse at Washington Hospital Center said ambulances continued to arrive Monday afternoon despite the staff's struggles to remain organized without their computers.

Eventually, she said, ambulances carrying patients with conditions not deemed life-threatening were diverted elsewhere.

Still, problems persisted throughout the evening. The nurse said she noticed a number of paper charts with pages that lacked labels containing each patient's identifying information. Without those labels, she said, documents could be placed on the wrong chart.

"There are a lot of people who have never done paper charting before, so it was a little chaotic for them," she said. "I think the biggest fear that I had when I was working yesterday was the big opportunity for error."

Derek Farwagi, 73, learned of the crisis Tuesday when he arrived at MedStar Georgetown University Hospital for his quarterly appointment with a kidney specialist. A doctor, he said, told him that they couldn't access his health records, so his appointment had been canceled.

"It's nuts that they don't have a crisis-management system," Farwagi said. "It's absolutely irresponsible."

The doctor also told him, he said, that "the hackers were demanding a ransom."

A spokesman for the FBI declined to comment on the investigation, which comes just weeks after cyberattacks on at least three medical institutions in California and Kentucky. In one case last month, a hospital in Los Angeles paid hackers $17,000 in bitcoins to free its system.

In the Washington area, patients with significant medical conditions encountered treatment delays.

Cynthia Decker, who underwent a kidney transplant in December, had an appointment scheduled at MedStar Georgetown on Monday, but she received a call from a nurse practitioner just before she arrived at the facility.

"All the computers are down," she recalled the nurse practitioner saying. "Don't come in."

Her appointment was moved to Tuesday, but MedStar canceled Tuesday morning.

"They're down again," a staffer told her at 8 a.m.

The spouse of a man receiving cancer treatment at one MedStar facility told The Post that he has been unable to receive radiation treatment for two days because of the shutdown. The spouse - concerned by MedStar's assertion on Monday that "facilities remain open and functioning" - said that "the individuals most dependent on reliable, safe and uninterrupted treatment (i.e. cancer patients) are in fact currently not receiving at least some of those treatments."

MedStar officials acknowledged treatment delays.

On Monday, they stressed that they had "acted quickly" to contain the virus by shutting down their computer systems and had found "no evidence that information has been stolen."

john.cox@washpost.com

Source: Washington Post, The, 03/30/2016
Item: wapo.252626ae-f5bc-11e5-a3ce-f06b5ba21f33

Major Florida hospital hit by possible ransomware attack - A major hospital system in northern Florida says it is diverting some emergency room patients and canceling surgeries after an security problem with information technology

February 3, 2023 | Independent, The/The Independent on Sunday: Web Edition Articles (London, England)
Author/Byline: Via AP news wire
191 Words

A major hospital system in northern Florida said Friday it is diverting some emergency room patients and canceling surgeries after a security problem with information technology.

Tallahassee Memorial HealthCare said the issue began effecting its systems late Thursday night and has forced the hospital to shut down its IT network.

It had the hallmarks of a ransomware attack, but the hospital has not yet characterized it as such, instead calling it an “IT security issue.” Victims often at least initially decline to confirm ransomware attacks.

The hospital said in a statement that it was diverting some emergency room patients, and rescheduling non-emergency patient appointments through Monday. It said it is not moving patients currently in the hospital to other facilities.

Patients will be contacted directly if their appointments are affected, spokeswoman Tori Lynn Schneider said in a statement.

It was unclear when systems would fully return online. The hospital said it has been working with law enforcement.

The hospital, headquartered in Tallahassee, provides health care across 21 counties in northern Florida and southern Georgia, according to its website.

Cybersecurity firm Emsisoft said there were 25 ransomware attacks involving hospitals or hospital systems last year.

© Copyright 2023 Independent News & Media Ltd., All Rights Reserved.

Record: 1

'Major disruption' as UK hospitals hit by cyber attack Al Jazeera (Qatar). 05/13/2017. (*AN: 2W6163823976*)

Database: Points of View Reference Center

'Major disruption' as UK hospitals hit by cyber attack

May 13--A ransomware virus has reportedly targeted organisations in more than 70 countries, including the Russian Interior Ministry and the NHS in the UK.

Friday's attacks came via a technique used by hackers that locks a user's files unless they pay the attackers in bitcoin. Computers affected by the virus went into lockdown by a programme that demanded $300 for the files to be unlocked.

Security software company Avast reported "more than 57,000 detections".

"We have observed a massive peak in WanaCrypt0r 2.0 (aka WCry) ransomware attacks today, with more than 57,000 detections so far," Avast said on its blog.

"According to our data, the ransomware is mainly being targeted to Russia, Ukraine and Taiwan, but the ransomware has successfully infected major institutions, like hospitals across England and Spanish telecommunications company, Telefonica."

Hospitals and doctors' surgeries across Britain were forced to turn away patients and cancel appointments after the cyberattack crippled some computer systems in the country's health service.

The National Health Service (NHS) said 16 organisations had been affected by the attack.

It said in a statement that the NHS had not been specifically targeted, adding that the attack was affecting organisations from across a range of sectors.

"The investigation is at an early stage but we believe the malware variant is Wanna Decryptor," the NHS said.

"At this stage we do not have any evidence that patient data has been accessed."

Britain's National Cyber Security Centre, part of the GCHQ spy agency, said it was aware of a cyber incident and was working with the NHS and the police to investigate.

"It certainly caused a meltdown in their [hospitals'] email servers, crashed computers, and then a message came up with a threat of a ransom, that all the files will be deleted permanently if the payment was not made," Al Jazeera's Sonia Gallego, reporting from the UK capital, London, said.

FedEx said it was experiencing issues with some of its Windows systems in relation to the attack.

"Like many other companies, FedEx is experiencing interference with some of our Windows-based systems caused by malware," a spokeswoman said in a statement.

Also on Friday, Spain's government said a large number of companies, including telecommunications giant Telefonica, had been attacked by cyber criminals who infected computers with ransomware.

Hospitals across England reported the cyber attack was causing huge problems to their services and the public in areas affected were being advised to seek medical care only for emergencies.

A reporter from the Health Service Journal said the attack had affected X-ray imaging systems, pathology test results, phone systems and patient administration systems.

The Barts Health group, which manages major central London hospitals including the Royal London and St Bartholomew's, said it had activated a major incident plan and had cancelled routine appointments.

"We are experiencing a major IT disruption and there are delays at all of our hospitals," it said.

"Ambulances are being diverted to neighbouring hospitals."

Derbyshire Community Health Services said in a Twitter post: "We are aware of a major IT secure system attack. All IT systems have been temporarily shut down."

Blackpool Hospitals NHS Trust, which includes six hospitals, said: "Please don't attend A&E unless it's an emergency," and, "Please avoid contacting your GP practice unless absolutely necessary."

There was no immediate comment from the health ministry or from Prime Minister Theresa May who was out campaigning in northeast England ahead of the general election on June 8.

Britain's opposition Labour Party said the attack on English hospitals showed the need to place cyber security at the heart of government policy.

"This incident highlights the risk to data security within the modern health service and reinforces the need for cyber security to be at the heart of government planning," Labour's health spokesman Jonathan Ashworth said.

Source: Al Jazeera and news agencies

___ (c)2017 Al Jazeera (Doha, Qatar) Visit Al Jazeera (Doha, Qatar) at www.aljazeera.com Distributed by Tribune Content Agency, LLC.

Copyright of **Al Jazeera (Qatar)** is the property of Al Jazeera (Qatar). The copyright in an individual article may be maintained by the author in certain cases. Content may not be copied or emailed to multiple sites or posted to a listserv without the copyright holder's express written permission. However, users may print, download, or email articles for individual use.

Source: Al Jazeera (Qatar), May 13, 2017
Item: 2W6163823976

Los Angeles Hospital Pays Hackers To Regain Control Of Medical Records

February 18, 2016 | All Things Considered [NPR] (USA)
All Things Considered | Section: All Things Considered
386 Words

ARI SHAPIRO: A Los Angeles hospital says it paid a ransom of 40 bitcoins or about $17,000 to hackers who shut down its computer system for days. Hollywood Presbyterian Medical Center says its servers are back online now, and patients' safety was never compromised. NPR's Kirk Siegler reports cyber security experts aren't so sure.

KIRK SIEGLER: Hollywood Presbyterian and law enforcement still haven't said how the malware infected the hospital's computers, but cyber security experts say it's likely that someone unknowingly clicked on a link or opened an email they shouldn't have. And just like that, the hospital servers were locked by thieves demanding ransom to turn them over.

CLIFFORD NEUMAN: Because our systems are so vulnerable, this is sort of a high payoff way for criminals to monetize their hack.

KIRK SIEGLER: Clifford Neuman heads the Center for Computer System Security at USC. He says the hospital did the right thing by shutting everything down and reverting to writing medical records by hand straight away. But the fact is the hackers only needed a few seconds to access all that data in sensitive files.

CLIFFORD NEUMAN: They don't necessarily know that it didn't send copies out of their system to somewhere else. So there's always a potential that the privacy of medical records were compromised as well.

KIRK SIEGLER: Our medical records contain our Social Security numbers, our medical history that advertisers and marketers are hungry for among other sensitive things. And Neuman says this case shows how hospitals, companies, firms big and small need tougher antivirus software and they need to back up their systems. But they should also rethink whether so many employees need access to huge servers at all times. Elizabeth Lucas, CEO of a hacker education company called Decoded, says there's one thing that can never be fully controlled, us.

ELIZABETH LUCAS: There's nothing that your, you know, IT department can do in terms of preventing, you know, the natural curiosity that we have as human beings to click on something when you get an email

KIRK SIEGLER: Lucas says we're surprised and alarmed by the Hollywood Presbyterian case, but this sort of ransomeware attack is actually becoming more and more common. She says most firms pay the ransom quickly and quietly because they don't want their reputations tarnished. Kirk Siegler, NPR News, Culver City, Calif.

© Copyright © 2016 NPR. All rights reserved. Visit our website terms of use and permissions page at www.npr.org for further information.

Record: 1

BRIEF: Lab, diagnostic services still unavailable at Heritage Valley satellite locations By: Stonesifer, Jared. Beaver County Times, The (PA). 06/29/2017. (*AN: 2W63088157246*)

Database: Points of View Reference Center

BRIEF: Lab, diagnostic services still unavailable at Heritage Valley satellite locations

~~~~~~~~

Jared Stonesifer

June 29--Heritage Valley Health System's satellite locations continued to be hampered by the effects of Tuesday's cyber attack.

The company announced Thursday that all lab and diagnostic services at neighborhood and community locations would remain closed for the second consecutive day as Heritage Valley officials worked to fully rectify the issue.

The company's entire computer system went down Tuesday morning during a widespread ransomware attack that affected businesses and governments in Europe and the United States.

Heritage Valley officials continued to investigate the cyber attack and whether any patient information was compromised.

While lab and diagnostic services are unavailable at community locations, those services were still being offered at the Heritage Valley hospitals in Brighton Township and Sewickley.

No timeline was given on when the community locations might be fully restored to service, or when Heritage Valley will release the finds of its investigation.

___ (c)2017 the Beaver County Times (Beaver, Pa.) Visit the Beaver County Times (Beaver, Pa.) at www.timesonline.com Distributed by Tribune Content Agency, LLC.

Copyright of **Beaver County Times, The (PA)** is the property of Beaver County Times, The (PA). The copyright in an individual article may be maintained by the author in certain cases. Content may not be copied or emailed to multiple sites or posted to a listserv without the copyright holder's express written permission. However, users may print, download, or email articles for individual use.

Source: Beaver County Times, The (PA), Jun 29, 2017

Item: 2W63088157246
~~~~~~~~

Record: 1

Title: Cyber attack hits Israeli hospitals

Source: Globes (Israel). 06/29/2017.

Document Type: Article

Accession Number: 2W6429000122

Database: Newspaper Source

Cyber attack hits Israeli hospitals

June 29--Computers in Israel's health system were attacked yesterday. Computers of several hospitals, including Hadassah Ein Kerem, Hadassah Mount Scopus, and Poriya Medical Center in Tiberias, were breached, Walla! reports. Shortly after the attack began, the Ministry of Heath computer department began to repel the attack in cooperation with the National Cyber Defense Authority.

The National Cyber Defense Authority said, "During the night, there were attempts to attack a number of hospitals. Several computers at several hospitals were infected; they were immediately handled, and no damage was caused. The event was stopped by information security personnel at the organizations involved, together with the National Cyber Defense Authority, and is being carefully monitored and handled. Work at the hospitals is continuing without interruption, no damage was caused, and the functioning of the hospitals was not affected."

Two days ago, a series of companies in Europe and the US were the targets of a major cyber attack, including demand for ransoms by the hackers, as happened last month, when use was made of the WannaCry ransomware. The National Cyber Defense Authority said that it was "studying and analyzing the details of the large-scale ransomware attack in Europe."

Experts at many companies identified the ransomeware used today as Petya, which prevents computers from operating by encrypting their disc drives and demanding money for the password to open them. The experts believe that the attack once again utilized the break-in tool of the US National Security Agency, which was leaked to Wikileaks.

Last month, the UK National Health Service (NHS) was damaged in a large-scale cyber attack. UK newspaper "The Guardian" reported that malware had penetrated systems of patients' data and appointments registration, and had blocked them, as well as telephone lines and email accounts relating to the NHS.

Additional organizations were paralyzed in the UK, Italy, Spain, Portugal, Russia, Ukraine, the US, Taiwan, and other countries. The attack, which initially focused on 11 European countries, later spread to 74 countries all over the world.

___ (c)2017 the Globes (Tel Aviv, Israel) Visit the Globes (Tel Aviv, Israel) at www.globes.co.il/serveen/globes/nodeview.asp?fid=942 Distributed by Tribune Content Agency, LLC.

Copyright of **Globes (Israel)** is the property of Globes (Israel). The copyright in an individual article may be maintained by the author in certain cases. Content may not be copied or emailed to multiple sites or posted to a listserv without the copyright holder's express written permission. However, users may print, download, or

email articles for individual use.

Source: Globes (Israel), Jun 29, 2017
Item: 2W6429000122

Disclaimer: This is a machine generated PDF of selected content from our products. This functionality is provided solely for your convenience and is in no way intended to replace original scanned PDF. Neither Cengage Learning nor its licensors make any representations or warranties with respect to the machine generated PDF. The PDF is automatically generated "AS IS" and "AS AVAILABLE" and are not retained in our systems. CENGAGE LEARNING AND ITS LICENSORS SPECIFICALLY DISCLAIM ANY AND ALL EXPRESS OR IMPLIED WARRANTIES, INCLUDING WITHOUT LIMITATION, ANY WARRANTIES FOR AVAILABILITY, ACCURACY, TIMELINESS, COMPLETENESS, NON-INFRINGEMENT, MERCHANTABILITY OR FITNESS FOR A PARTICULAR PURPOSE. Your use of the machine generated PDF is subject to all use restrictions contained in The Cengage Learning Subscription and License Agreement and/or the Gale General OneFile Terms and Conditions and by using the machine generated PDF functionality you agree to forgo any and all claims against Cengage Learning or its licensors for your use of the machine generated PDF functionality and any output derived therefrom.

In another case tonight, Comey`s FBI is leading the investigation of a hostage situation at a California hospital

Date: 2016
From: CBS Evening News
Publisher: CQ-Roll Call, Inc.
Document Type: Brief article; Broadcast transcript
Length: 275 words

Full Text:
SCOTT PELLEY: In another case tonight, Comey`s FBI is leading the investigation of a hostage situation at a California hospital. But it`s not people being held. It`s the computer system. Carter Evans is in Los Angeles.

(Begin VT)

CARTER EVANS: Inside Hollywood Presbyterian Medical Center, computer screens have been dark since hackers took over the data network almost two weeks ago. Calls to the hospital`s media line are met with this voicemail recording.

WOMAN (voicemail recording): We want to assure you that patient care at CHA Hollywood Presbyterian has not been compromised, as we continue to address this incident.

CARTER EVANS: The attack used what`s known as "ransomware," malicious software that encrypts files which can only be unlocked with a software key after a ransom is paid. In this case, according to a source familiar with the investigation hackers demanded and the hospital paid an undisclosed amount in the computer currency bitcoin, which is nearly impossible to trace. Since the attack, the medical center staff has resorted to pen and paper and even fax machines for communications.

(End VT)

CARTER EVANS: The FBI confirmed the attack but declined to comment on its investigation. And, Scott, Hollywood Presbyterian has not responded to CBS News` requests.

SCOTT PELLEY: Carter Evans, thanks very much.

END

[Copy: Content and programming Copyright MMXVI CBS Broadcasting Inc. ALL RIGHTS RESERVED. Copyright 2016 CQ-Roll Call, Inc. All materials herein are protected by United States copyright law and may not be reproduced, distributed, transmitted, displayed, published or broadcast without the prior written permission of CQ-Roll Call. You may not alter or remove any trademark, copyright or other notice from copies of the content.]

Copyright: COPYRIGHT 2016 CQ-Roll Call, Inc.
http://www.thenewsroom.com/
Source Citation (MLA 9th Edition)
"In another case tonight, Comey`s FBI is leading the investigation of a hostage situation at a California hospital." *CBS Evening News*, 17 Feb. 2016. *Gale General OneFile*, link.gale.com/apps/doc/A443640030/ITOF?u=naal_aub&sid=ebsco&xid=f051eaaa. Accessed 16 Sept. 2023.
Gale Document Number: GALE|A443640030

Record: 1

Title: BRIEF: Heritage Valley lab draw services available as of Saturday morning

Authors: Sheleheda, Christina

Source: Beaver County Times, The (PA). 07/01/2017.

Document Type: Article

Accession Number: 2W61350647081

Database: Points of View Reference Center

BRIEF: Heritage Valley lab draw services available as of Saturday morning

~~~~~~~~

Christina Sheleheda

July 01--Heritage Valley Health System's community lab draw services are up and running after being down since Tuesday.

According to the organization's website, as of Saturday morning, services at Heritage Valley Chippewa, Heritage Valley Edgeworth and Heritage Valley Moon Township Medical Neighborhoods are open for regularly scheduled hours.

The company's computer system went down Tuesday morning following a ransomware attack that affected most of their community locations.

Heritage Valley officials are still investigating the attack. Information will be added as it becomes available.

___ (c)2017 the Beaver County Times (Beaver, Pa.) Visit the Beaver County Times (Beaver, Pa.) at www.timesonline.com Distributed by Tribune Content Agency, LLC.

Copyright of **Beaver County Times, The (PA)** is the property of Beaver County Times, The (PA). The copyright in an individual article may be maintained by the author in certain cases. Content may not be copied or emailed to multiple sites or posted to a listserv without the copyright holder's express written permission. However, users may print, download, or email articles for individual use.
Source: Beaver County Times, The (PA), Jul 01, 2017
Item: 2W61350647081
~~~~~~~~

Title: Heritage Valley still dealing with effects of Tuesday's cyber attack By: Stonesifer, Jared, Beaver County Times, The (PA), Jun 28, 2017

Database: Points of View Reference Center

Heritage Valley still dealing with effects of Tuesday's cyber attack

This content may contain URLs/links that would redirect you to a non-EBSCO site. EBSCO does not endorse the accuracy or accessibility of these sites, nor of the content therein.

Listen

~~~~~~~~

Jared Stonesifer

June 28--SEWICKLEY -- When Jamie Davis went to her doctor's office early Tuesday morning, she didn't think twice about the office's computer system being down.

But it didn't take long for the Shippingport resident to learn the true nature of the incident -- that Heritage Valley and all of its satellite locations had been hacked as part of a global cybersecurity attack that affected business and governments across Europe and the United States.

Davis' experience at the doctor was something akin to a medical visit in the time before computers. She said the doctor dictated instructions to an assistant, who jotted down notes on a blank sheet of paper.

Despite that, her doctor couldn't access any of her medical records, and both patient and caregiver had to go off memory in regards to past medical history and medications.

"They couldn't get to my (electronic) file and had to do everything with a blank sheet of paper," Davis said. "The entire experience was very old-school. We had to go by memory, which wasn't bad except I
~~~~~~~~

couldn't remember what medication they put me on."

The appointment ended without any mishaps, but Davis said her mind started to wander throughout the day Tuesday. Her thoughts turned to the personal information contained in her medical files, and whether that information had fallen into the wrong hands.

"It did cross my mind with my medical information, my Social Security number, my birthday, my mom's maiden name," Davis said. "All of that information is in there."

For its part, Heritage Valley spokeswoman Suzanne Sakson said the company is working "diligently" to determine if the hackers gained access to any confidential information or records.

"At this time we have found no evidence that information has been accessed, but our investigation continues," she said.

She also said that protecting patient information is a "priority" for the company.

Regardless, Heritage Valley announced early Wednesday that lab and diagnostic services at its neighborhood and community locations were unavailable as the company continued to deal with the effects of the cyber attack.

Heritage Valley officials didn't say whether medical appointments or procedures were rescheduled because of the hack, but Davis said she saw patients being rescheduled at the doctor's office Tuesday morning.

"Some people were being cancelled by the doctor's office, but they couldn't reschedule them because they didn't have access to a computer," Davis said.

As of Wednesday afternoon, Heritage Valley was still working to rectify issues caused by the cyber attack, and Sakson said the company is "confident that it has identified the cause and is systematically restoring registration, clinical patient and ancillary care systems.

"Information technology teams in collaboration with cyber security experts continue to mitigate the situation in order to ensure ongoing safe patient care," she said.

Tuesday's cyber attack was the second large-scale ransomware outbreak in the last three months, according to the Washington, D.C.-based nonprofit National Cyber Security Alliance.

Ransomware is a type of malicious software that blocks user access to computers until money is paid as ransom.

The cyber security alliance, which strives to enhance cybersecurity education and awareness, said ransomware attacks are evolving as hackers "become more sophisticated" and intricate in their attacks.

In addition, these attacks prove to be much more than just temporary headaches for companies, governments and people affected.

"It is much more than an inconvenience," Michael Kaiser, executive director of the National Cyber Security Alliance, said. "We are more dependent than ever on the data stored across our computer systems. It is our shared responsibility to do our part in keeping devices secure. It all starts with basic cyber hygiene around software updates and locking down logins."

While attacks continue to become more sophisticated, Kaiser said regular citizens can do their part to ensure they don't become victims of cyber attacks.

He said people should continually update their devices with software patches or fixes, and use strong password or other authentication measures.

In addition, people should regularly conduct backups of their systems, meaning their systems can be restored if ransomware or other attacks block them from accessing their computers.

"Prevention is clearly the goal," Kaiser said. "However, organizations and individuals should also be prepared to respond to and recover from an attack to minimize downtime and disruptions."

On Wednesday afternoon, Davis suggested that hospitals and doctor's offices employ backup measures that will ensure records can be accessed if computers aren't available.

"Maybe they should keep paper forms as well as digital copies," she said. "Because when things like this happen again -- and they will happen again -- they have something to go by. They will need more storage space but at least they'll have something as backup."

___ (c)2017 the Beaver County Times (Beaver, Pa.) Visit the Beaver County Times (Beaver, Pa.) at www.timesonline.com Distributed by Tribune Content Agency, LLC.

Copyright of **Beaver County Times, The (PA)** is the property of Beaver County Times, The (PA). The copyright in an individual article may be maintained by the author in certain cases. Content may not be copied or emailed to multiple sites or posted to a listserv without the copyright holder's express written

permission. However, users may print, download, or email articles for individual use. **Source:** Beaver County Times, The (PA), Jun 28, 2017

Item: 2W61848661592

Title: Hospital Sisters Health System restores health record access By: Wells, Valerie, Herald & Review (Decatur, IL), Sep 12, 2023

Database: Points of View Reference Center

Hospital Sisters Health System restores health record access

This content may contain URLs/links that would redirect you to a non-EBSCO site. EBSCO does not endorse the accuracy or accessibility of these sites, nor of the content therein. ✕

Listen

~~~~~~~~

Valerie Wells

Sep. 12—DECATUR — Hospital Sisters Health Systems has successfully restored its electronic health records system, which includes MyChart applications, officials said Tuesday.

A "cybersecurity incident" on Aug. 27 caused a massive operating system outage for HSHS hospitals and facilities. Those included St. Mary's Hospital in Decatur, St. Anthony's Memorial Hospital in Effingham, Good Shepherd Hospital in Shelbyville and St. John's Hospital in Springfield, as well as HSHS Medical Group, Prairie Cardiovascular entities, HSHS St. John's College of Nursing and community clinic partners.

The cybersecurity incident affected most communications systems.

A website devoted to updates on the situation states: "We will respond to patient messages as quickly as possible, and we encourage patients to reach out to their health care provider's office to speak with a member of their health care team, should they require urgent assistance.
~~~~~~~~

"We remain focused on restoring the rest of our systems in a methodical manner, which will take time to complete. We appreciate your continued patience and look forward to continuing to care for our valued patients."

Patients who have sent messages to the health system will receive answers as quickly as possible, and patients can again use MyChart to schedule appointments. For urgent matters, the site says, patients should contact their provider's office by telephone.

It still remains unclear whether confidential patient information has been compromised. HSHS President and CEO Damond Boatwright said an investigation into the scope and impact of the breach is ongoing.

Contact Valerie Wells at (217) 421-7982. Follow her on Twitter: @modgirlreporter

___ (c)2023 the Herald & Review (Decatur, Ill.) Visit the Herald & Review (Decatur, Ill.) at www.herald-review.com Distributed by Tribune Content Agency, LLC.

Copyright of **Herald & Review (Decatur, IL)** is the property of Herald & Review (Decatur, IL). The copyright in an individual article may be maintained by the author in certain cases. Content may not be copied or emailed to multiple sites or posted to a listserv without the copyright holder's express written permission. However, users may print, download, or email articles for individual use. **Source:** Herald & Review (Decatur, IL), Sep 12, 2023
Item: 2W63100275001

Hospital Sisters Health System still battling 'cybersecurity incident'

September 1, 2023 | Pantagraph, The (Bloomington, IL)
Author/Byline: ALLISON PETTY allison.petty@lee.net | Section: State And Regional
385 Words

DECATUR — Hospital Sisters Health System continues to recover after a massive operating system outage caused by what its CEO confirmed Friday was a "cybersecurity incident."

HSHS, which runs St. Mary's Hospital in Decatur, St. Anthony's Memorial Hospital in Effingham, Good Shepherd Hospital in Shelbyville and HSHS St. John's Hospital in Springfield, began to experience the outage early Sunday. Most communications systems and hospital and clinic operations were affected, although the health system said its staff efficiently executed the plan for such situations.

It is not clear whether confidential patient information has been compromised. HSHS President and CEO Damond Boatwright said an investigation into the scope and impact of the breach was ongoing.

"I am relieved to report we are making progress," he said, in a video the health system posted online Friday night. "We are bringing back critical systems and have restored many of our internal communications applications, like email and messaging."

The hospital system's information technology staff and "outside experts" were laboring nonstop to restore the remaining services, he said.

Friday's message also marked the health system's first indication as to the cause of the outage: "Based on our findings, we can confirm this was the result of a cybersecurity incident," Boatwright said, adding that more information would be released as it becomes available.

He said the health system was working with law enforcement. An FBI spokeswoman previously said the agency would not confirm or deny whether it was involved in an investigation.

"Your patience and understanding mean the world to us," Boatwright said. "Please know our top priority is protecting patient safety and supporting our colleagues so they can take care of the patients we serve."

The health system said further updates would be provided at hshsupdates.org.

The attack comes weeks after one that took hospitals and clinics in several states offline for weeks. The attack on Prospect Medical Holdings, which runs facilities in California, Connecticut, Pennsylvania, Rhode Island and Texas, forced postponement of some elective surgeries, outpatient appointments, blood drives and other services.

Globally, the healthcare industry was the hardest-hit by cyberattacks in the year ending in March, according to IBM's annual report on data breaches. For the 13th straight year it reported the most expensive breaches, averaging $11 million each. Next was the financial sector at $5.9 million.

The Associated Press contributed.

© Copyright (c) 2023, Pantagraph Publishing Co.

Record: 1

Hospital chain attack part of ongoing cybersecurity concerns By: By KATHLEEN FOODY and KIMBERLEE KRUESI - Associated Press. AP Regional State Report - Illinois, 10/06/2022; Abstract: CHICAGO (AP) ” Diverted ambulances. Cancer treatment delayed. Electronic health records offline. These are just some of ripple effects of an apparent cyberattack on a major nonprofit health system that disrupted operations throughout the U.S. While CommonSpirit Health confirmed it experienced an œIT security issue earlier this week, the company has remained mum when pressed for more details about the scope of the attack. The health system giant has 140 hospitals in 21 states. As of Thursday, it's still unknown how many of its 1,000 care sites that serve 20 million Americans were affected. [ABSTRACT FROM PUBLISHER] (*AN AP0066b8082e1882e00b630ac5041007a8*)

Database: Newswires

Hospital chain attack part of ongoing cybersecurity concerns

Hospital chain attack part of ongoing cybersecurity concerns

~~~~~~~~

By KATHLEEN FOODY and KIMBERLEE KRUESI

CHICAGO (AP) ” Diverted ambulances. Cancer treatment delayed. Electronic health records offline. These are just some of ripple effects of an apparent cyberattack on a major nonprofit health system that disrupted operations throughout the U.S.

While CommonSpirit Health confirmed it experienced an œIT security issue earlier this week, the company has remained mum when pressed for more details about the scope of the attack. The health system giant has 140 hospitals in 21 states. As of Thursday, it's still unknown how many of its 1,000 care sites that serve 20 million Americans were affected.

Despite the lingering questions, the incident underscores the growing concerns surrounding ransomware attacks on health care systems with patient care at stake.

In Tacoma, Washington, Mark Kellogg told KING-TV that his wife, Kathy, had been scheduled to get a cancerous tumor on her tongue removed on Monday, but the procedure was put off several days because of the cyberattack. Virginia Mason Franciscan Health's parent company is CommonSpirit Health.

œEverything we do today is all on a computer, and without it you™re back to the stone age writing on a tablet, Kellogg said.

In Iowa, the Des Moines Register reported that the incident forced the diversion of five ambulances from the emergency department of the city™s MercyOne Medical Center to other medical facilities.

The incident forced both MercyOne and VMFH to take certain IT systems offline ” including patients' electronic health records ” as a precaution.
~~~~~~~~

Brett Callow, a threat analyst with cybersecurity provider Emsisoft, said the incident could be œthe most significant attack on the health care sector to date if all CommonSpirit hospitals and other facilities were affected.

Emsisoft has tracked at least 15 health care systems in the U.S. affected by ransomware this year, which manage more than 60 hospitals. Callow said data was stolen in 12 of the 15 instances, adding that those are almost surely undercounts as some ransomware attacks aren™t widely reported.

Callow said one of the largest known attacks within health care came in September 2020 when a ransomware attack struck all 250 health care facilities owned by Universal Health Services.

CommonSpirit™s incident could exceed that, depending on how many of its facilities were hit. That could mean the company faces large financial costs to get through the incident and recover.

Callow cited the loss of more than $100 million reported by Scripps Health tied to a 2021 ransomware attack that affected its five hospitals in California as an example.

Asked for more information on the incident and its effects on Thursday, a spokesperson for CommonSpirit said the health system could not provide more details.

The most worrying effect of any substantial attack on healthcare is on patients, Callow said.

œI™ve seen reports that at least one of the impacted hospitals had to divert ambulances to other facilities and that delay in getting people the care they need could obviously represent a risk to the lives of patients, he said. œBeyond that, these incidents can have a long-term impact on patient outcomes ” delaying treatments, for example.

In 2020, the FBI and other federal agencies warned that they had credible information that cybercriminals could unleash a wave of data-scrambling extortion attempts against U.S. hospitals and health care providers.

That's because ransomware criminals are increasingly stealing data from their targets before encrypting networks, using it for extortion. They often sow the malware weeks before activating it, waiting for moments when they believe they can extract the highest payments.

Health care is classified by the U.S. government as one of 16 critical infrastructure sectors Health care providers are seen as ripe targets for hackers.

If patient data is accessed, health care providers are required by law to notify the Department of Health and Human Services.

Kruesi reported from Nashville, Tennessee.

Copyright of **AP Regional State Report - Illinois** is the property of Associated Press DBA Press Association. The copyright in an individual article may be maintained by the author in certain cases. Content may not be copied or emailed to multiple sites or posted to a listserv without the copyright holder's express written permission. However, users may print, download, or email articles for individual use.

Source: AP Regional State Report - Illinois,

Item:

Disclaimer: This is a machine generated PDF of selected content from our products. This functionality is provided solely for your convenience and is in no way intended to replace original scanned PDF. Neither Cengage Learning nor its licensors make any representations or warranties with respect to the machine generated PDF. The PDF is automatically generated "AS IS" and "AS AVAILABLE" and are not retained in our systems. CENGAGE LEARNING AND ITS LICENSORS SPECIFICALLY DISCLAIM ANY AND ALL EXPRESS OR IMPLIED WARRANTIES, INCLUDING WITHOUT LIMITATION, ANY WARRANTIES FOR AVAILABILITY, ACCURACY, TIMELINESS, COMPLETENESS, NON-INFRINGEMENT, MERCHANTABILITY OR FITNESS FOR A PARTICULAR PURPOSE. Your use of the machine generated PDF is subject to all use restrictions contained in The Cengage Learning Subscription and License Agreement and/or the Gale General OneFile Terms and Conditions and by using the machine generated PDF functionality you agree to forgo any and all claims against Cengage Learning or its licensors for your use of the machine generated PDF functionality and any output derived therefrom.

HOSPITAL CHAIN SAYS ‘IT SECURITY ISSUE’ DISRUPTS OPERATIONS.

Date: Oct. 8, 2022
From: Techlife News
Publisher: Ivan Castilho de Almeida
Document Type: Brief article
Length: 209 words

Full Text:
A major nonprofit health system with 140 hospitals in 21 states, CommonSpirit Health, is reporting an “IT security issue” that has disrupted operations in multiple states.

A company spokesperson would not explain the nature of the apparent cyberattack, such as whether the organization’s IT network was hit by ransomware.

The Des Moines Register said the incident occurred this week and forced the diversion of ambulances from the emergency department of the city’s Mercy One Medical Center to other medical facilities. The Chattanoogan reported that CHI Memorial Hospital was among facilities impacted.

In a statement, CommonSpirit said it had taken “certain IT systems offline” including electronic health records as a precaution and rescheduled some patient appointments. It would not say whether patient records were accessed. Nor did it say when the apparent breach was detected.

The Chicago company, formed in 2019 from the alignment of Catholic Health Initiatives and Dignity Health, serves 20 million Americans with more than 1,000 care sites located coast-to-coast.

Health care is classified by the U.S. government as one of 16 critical infrastructure sectors, and health care providers are seen as ripe targets for hackers.

If patient data is accessed, health care providers are required by law to notify the Department of Health and Human Services.

Copyright: COPYRIGHT 2022 Techlife News
Source Citation (MLA 9th Edition)
"HOSPITAL CHAIN SAYS ‘IT SECURITY ISSUE’ DISRUPTS OPERATIONS." *Techlife News*, 8 Oct. 2022, p. 138. *Gale General OneFile*, link.gale.com/apps/doc/A736636992/ITOF?u=naal_aub&sid=ebsco&xid=b96a646c. Accessed 16 Sept. 2023.
Gale Document Number: GALE|A736636992

Title: Heritage Valley continues to recover from cyberattack By: Schmitt, Ben, Pittsburgh Tribune Review (PA), Jun 30, 2017

Database: Points of View Reference Center

Heritage Valley continues to recover from cyberattack

This content may contain URLs/links that would redirect you to a non-EBSCO site. EBSCO does not endorse the accuracy or accessibility of these sites, nor of the content therein. ✕

Listen

~~~~~~~~

Ben Schmitt

June 30--Heritage Valley Health System said Friday that almost all its facilities are up and running after a cyberattack that disrupted its computer systems.

Heritage Valley Sewickley and Heritage Valley Beaver Hospitals, Heritage Valley Medical Group, Heritage Valley Pediatrics and Tri-State Obstetrics & Gynecology physician practices, ConvenientCare walk-in clinics and all other community locations are open and operational, its spokeswoman said in a press release.

Lab and diagnostic imaging services are fully functional and available at Heritage Valley Beaver and Heritage Valley Sewickley hospitals, but remain unavailable at the following locations:

--Heritage Valley Medical Neighborhoods in Chippewa, Edgeworth, West Allegheny, Moon Township, Calcutta, Ellwood City and Robinson Township.

--Ambridge Laboratory Draw -- 1155 Merchant Street, Ambridge.
~~~~~~~~

--Baden Laboratory Draw -- 220 Ohio River Boulevard, Baden.

--Beaver Falls Laboratory Draw -- 1125 Seventh Avenue, Beaver Falls.

--Heart & Vascular Laboratory Draw -- 605 Sharon Road, Beaver.

--Heritage Valley Women's Health Center -- 200 Pleasant Drive, Center Township.

--Hopewell Laboratory Draw and Diagnostics -- 5000 Industrial Blvd, Aliquippa and 2032 Brodhead Road, Hopewell.

Heritage Valley Health System first announced the attack Tuesday , and said the incident "has been identified as the same ransomware attack that affected a number of organizations globally.

Heritage Valley is a $480 million integrated delivery network providing comprehensive health care for residents of Allegheny, Beaver, Butler and Lawrence counties, in Pennsylvania; eastern Ohio; and the panhandle of West Virginia.

Ben Schmitt is a Tribune-Review staff writer. Reach him at 412-320-7991, bschmitt@tribweb.com or via Twitter at @Bencschmitt.

___ (c)2017 The Pittsburgh Tribune-Review (Greensburg, Pa.) Visit The Pittsburgh Tribune-Review (Greensburg, Pa.) at www.triblive.com Distributed by Tribune Content Agency, LLC.

Copyright of **Pittsburgh Tribune Review (PA)** is the property of Pittsburgh Tribune Review (PA). The copyright in an individual article may be maintained by the author in certain cases. Content may not be copied or emailed to multiple sites or posted to a listserv without the copyright holder's express written permission. However, users may print, download, or email articles for individual use. **Source:** Pittsburgh Tribune Review (PA), Jun 30, 2017
Item: 2W62251790279

Skip to Content

Library Menu: Auburn University (AVL)

English Select Language English

- Afrikaans
- العربية
- Bahasa Indonesia
- Bahasa Malaysia
- česky
- Cymraeg
- Dansk
- Deutsch
- English
- Español
- Français
- Gaeilge
- Hrvatski
- Italiano
- magyar
- ខ្មែរ
- Nederlands
- Polski
- Português
- Română
- Slovenščina
- slovenský
- suomi
- svenska
- Tagalog
- Tiếng Việt
- Türkçe
- Русский
- Ελληνικά
- বাংলা
- हिंदी
- தமிழ்
- ไทย
- 中文（简体）
- 中文（繁體）
- 日本語
- 한국어

Sign in with Google

Save documents, citations, and highlights to Google Drive™

Sign in with Microsoft

Save documents, citations, and highlights to Microsoft OneDrive™

[Gale In ContextOpposing Viewpoints]

Basic Search

Search...

Submit

[Advanced Search]

Cite

Send to...

Download

Print

Get Link

Highlights and Notes (0)

Highlights and Notes View All Highlights and Notes

Your session has timed out after 20 minutes of inactivity. If you do not click continue session, you will be logged out in 60 seconds

Heritage Valley Health System's community offices closed after cyberattack

Date: June 28, 2017
From: The Pittsburgh Tribune-Review (Pittsburgh, PA)
Publisher: Tribune-Review Publishing Company
Document Type: Brief article
Length: 252 words
Content Level: (Level 4)
Lexile Measure: 1210L

Full Text:

Byline: Ben Schmitt

June 28--Laboratory and diagnostic services at Heritage Valley's neighborhoods and community locations will be closed Wednesday as the health system continues to deal with a cyberattack that disrupted its computer systems. Heritage Valley Health System, which announced the attack Tuesday, said the incident "has been identified as the same ransomware attack that affected a number of organizations globally." Spokeswoman Suzanne Sakson said in an email that "corrective measures supplied by our antivirus software vendor have been developed and are being implemented and tested within the health system." Later Wednesday morning, she issued a second statement, which read: "At this time we have found no evidence that information has been accessed, but our investigation continues." She would not say whether patient care has been affected within the system, which operates Heritage Valley Beaver and Heritage Valley Sewickley. An update said lab and diagnostic services are available at those hospitals. "Protecting the confidentiality of patient information is our priority. Our investigation is ongoing and we are diligently working to determine whether the incident involved any access to confidential information," Sakson said. The system also operates surgery centers in Moon and Edgeworth. Sakson said other "restorative measures" are being implemented to address the problem. The Associated Press reported that the massive outbreak of malicious data-scrambling software hit companies and governments in Europe especially hard. Ben Schmitt is a Tribune-Review staff writer.

(c)2017 The Pittsburgh Tribune-Review (Greensburg, Pa.)

Visit The Pittsburgh Tribune-Review (Greensburg, Pa.) at www.triblive.com

Distributed by Tribune Content Agency, LLC.
Copyright: COPYRIGHT 2017 Tribune-Review Publishing Company
http://www.pittsburghlive.com/x/pittsburghtrib/

Source Citation

MLA 9th Edition APA 7th Edition Chicago 17th Edition Harvard

"Heritage Valley Health System's community offices closed after cyberattack." *Pittsburgh Tribune-Review* [Pittsburgh, PA], 28 June 2017. *Gale In Context: Opposing Viewpoints*, link.gale.com/apps/doc/A497199100/OVIC?u=naal_aub&sid=ebsco&xid=a5200409. Accessed 16 Sept. 2023.

Disclaimer

Select

Export To:

*The RIS file format can be used with EndNote, ProCite, Reference Manager, and Zotero.

NoodleTools EasyBib RefWorks Google Drive™ OneDrive™

Download RIS*

Gale Document Number: GALE|A497199100

Cancel Done

Record: 1

BRIEF: Heritage Valley Health System dealing with 'cybersecurity incident' By: Schmitt, Ben. Pittsburgh Tribune Review (PA). 06/27/2017. (*AN: 2W6799077389*)

Database: Points of View Reference Center

BRIEF: Heritage Valley Health System dealing with 'cybersecurity incident'

~~~~~~~~
Ben Schmitt

June 27--Heritage Valley Health System officials said Tuesday they are dealing with a "cybersecurity incident," although details are unclear as to its level of seriousness.

"Heritage Valley Health System has been affected by a cyber security incident" Spokeswoman Suzanne Sakson said in an email to the Trib. "The incident is widespread and is affecting the entire health system including satellite and community locations. We have implemented downtime procedures and made operational adjustments to ensure safe patient care continues un-impeded."

Heritage Valley Health System owns hospitals in Beaver and Sewickley and Ohio.

___ (c)2017 The Pittsburgh Tribune-Review (Greensburg, Pa.) Visit The Pittsburgh Tribune-Review (Greensburg, Pa.) at www.triblive.com Distributed by Tribune Content Agency, LLC.

Copyright of **Pittsburgh Tribune Review (PA)** is the property of Pittsburgh Tribune Review (PA). The copyright in an individual article may be maintained by the author in certain cases. Content may not be copied or emailed to multiple sites or posted to a listserv without the copyright holder's express written permission. However, users may print, download, or email articles for individual use.
Source: Pittsburgh Tribune Review (PA), Jun 27, 2017
Item: 2W6799077389
~~~~~~~~

"WE HACKED THE HACKERS" - FED SEIZE NOTORIOUS RANSOMWARE GROUP'S SITE, DERAILS $100 MILLION OPERATION

January 27, 2023 | Good Morning America (USA)
Author/Byline: MICHAEL STRAHAN/AARON KATERSKY NEW YORK, NY USA | GOOD MORNING AMERICA | Section: GOOD MORNING AMERICA
456 Words

(Off-camera) We're gonna turn now to a major cybercrime bust. Federal agents say they hacked the hackers, infiltrating the Hive ransomware group, who broke into schools and even hospitals. Aaron Katersky is here with the story. Good morning, Aaron.

MICHAEL STRAHAN (ABC NEWS)

GRAPHICS: "WE'VE HACKED THE FEDS SEIZE NOTORIOUS RANSOMWARE GROUP'S SITE, DERAILS $100 MILLION (Off-camera) Good morning to you, Michael. Those hackers, known as Hive, did sting where it hurts deploying malicious software at hospitals and schools, critical infrastructure, locking up computers and demanding a ransom. Well, now the US has hacked back.

AARON KATERSKY (ABC NEWS)

(Voiceover) This morning, the Russia linked hacking group known as Hive, considered one of the most prolific and dangerous, has been shut down.

AARON KATERSKY (ABC NEWS)

Simply put, using lawful means, we hacked the hackers.

LISA MONACO (DEPUTY US ATTORNEY GENERAL)

(Voiceover) The FBI and international police gained access to Hive, seizing its servers and the keys to decrypt its software.

AARON KATERSKY (ABC NEWS)

Preventing at least $130 million in ransom payments and cutting offer the gas that's fueling Hive's fire.

CHRISTOPHER WRAY (DIRECTOR FEDERAL BUREAU OF INVESTIGATION)

(Voiceover) Since Hive was detected in 2021, it has targeted thousands of victims taking in more than $100 million in extortion payments. Its hackers have been blamed for stealing the data of 270,000 patients at a medical complex in Louisiana. And as COVID cases were surging, it rendered a hospital in Ohio unable to accept new patients.

AARON KATERSKY (ABC NEWS)

The Hive has been targeting the health care system, universities and everyday Americans for some time now. So they've stolen well over $150 million. It's no joke.

SCOTT SPIRO (CYBER SECURITY EXPERT SUGARSHOT)

(Voiceover) For the last six months the FBI has been helping victims recover their files without paying a ransom, but here have been no arrests.

AARON KATERSKY (ABC NEWS)

Cybercriminals will up their game. Cybercrime is a constantly evolving threat, and I think this sends the right message, but I don't think this is the, you know, the end game by any stretch.

SCOTT SPIRO (CYBER SECURITY EXPERT SUGARSHOT)

(Off-camera) The authorities have long accused Russia of harboring ransomware groups like Hive, so they're not going away, but the FBI is hoping this case convinces companies to come forward if they've been targeted. That way investigators can try to trace the hack, maybe recover some of the money or, guys, make those companies not have to pay to get their stuff back.

AARON KATERSKY (ABC NEWS)

GRAPHICS: INSIDE THE CRYPTO (Off-camera) All right, Aaron, thank you for that. And coming up next, we're going to hear from the victims of the crypto crash. The average Americans who lost their savings when it collapsed.

MICHAEL STRAHAN (ABC NEWS)

COMMERCIAL BREAK

© Copyright © 2023 ABC News.

Title: Hackers Strike Another Hospital System By: Bergal, Jenni, Stateline.org (Washington, DC), Aug 19, 2021

Database: Points of View Reference Center

Hackers Strike Another Hospital System

This content may contain URLs/links that would redirect you to a non-EBSCO site. EBSCO does not endorse the accuracy or accessibility of these sites, nor of the content therein.

×

Listen

~~~~~~~~

Jenni Bergal

Aug. 19—A network of hospitals and clinics in Ohio and West Virginia was forced to cancel surgeries and divert patients with emergencies to other facilities after it was hit in a ransomware attack this week.

Cybercriminals struck Memorial Health System, a nonprofit that runs three hospitals, outpatient service sites and clinics spread across southeastern Ohio and northwestern West Virginia, early Sunday morning.

Officials said Memorial had to shut down its information technology systems and cancel urgent surgical cases scheduled for Monday. It also had to divert ambulances to other hospitals.

"Maintaining the safety and security of our patients and their care is our top priority and we are doing everything possible to minimize disruption," president and CEO Scott Cantley said in a written statement posted on the system's website. "Staff at our hospitals ... are working with paper charts while systems are restored, and data recovered."

Cantley said at a news conference that the health system was working with security partners, including the FBI and the Department of Homeland Security, to restore operations as soon as possible. He said
~~~~~~~~

there hadn't been any indication that patients' medical records were accessed.

On Wednesday, Memorial posted an update saying its systems could be restored as early as Sunday.

"We have reached a negotiated solution and are beginning the process that will restore operations as quickly and as safely as possible," Cantley wrote.

"It is unfortunate that many health care organizations are confronting the impacts of an evolving cyber threat landscape," he added, noting that Memorial will focus on beefing up its security.

Ransomware typically spreads through phishing, in which hackers email malicious links or attachments and people unwittingly click on them. Malware then hijacks the victim's computer system and holds it hostage until the victim either pays a ransom, usually with the cryptocurrency bitcoin, or restores the system on their own.

Since the pandemic began, cybersecurity experts say they have seen an uptick in attempted ransomware and other hacking attempts on hospitals, health care systems, clinical labs and research centers.

Hospitals often lag behind other industries such as financial services when it comes to cybersecurity, experts say. That makes them an ideal target for hackers, especially during the pandemic.

The hospitals' biggest fear is that if computer networks get locked up or knocked offline, health care workers won't be able to access important information such as patient medical records and test results.

___ (c)2021 Stateline.org Visit Stateline.org at www.stateline.org Distributed by Tribune Content Agency, LLC.

Copyright of **Stateline.org (Washington, DC)** is the property of Stateline.org (Washington, DC). The copyright in an individual article may be maintained by the author in certain cases. Content may not be copied or emailed to multiple sites or posted to a listserv without the copyright holder's express written permission. However, users may print, download, or email articles for individual use.
Source: Stateline.org (Washington, DC), Aug 19, 2021
Item: 2W61643549207

Record: 1

BRIEF: Grand jury indicts guard accused of hacking computer system at Dallas clinic By: Selk, Avi. Dallas Morning News, The (TX). 07/23/2009. (*AN: 2W63491596427*)

Database: Points of View Reference Center

BRIEF: Grand jury indicts guard accused of hacking computer system at Dallas clinic

~~~~~~~~
Avi Selk

Jul. 23--A contract security guard accused of hacking into a computer system at a Dallas medical clinic was indicted on two counts of transmitting malicious code, the U.S. Attorney's office said today.

If convicted, Jesse William McGraw, 25 -- known online as "GhostExodus" and the leader of a hacker group called Electronik Tribulation Army -- could face up to 10 years in prison and a $250,000 fine, according to the U.S. Attorney's office for the Northern District of Texas.

The Arlington man was arrested in June and accused of using his security access at the Carrell Clinic on North Central Expressway to upload viruses to its patient-record and air conditioning-control systems. The FBI also claims his online group planned a massive denial-of-service attack on July 4 and hacked into a Dallas Police Department computers, among others.

At a hearing earlier this month, McGraw's lawyer did not dispute he hacked the clinic's computers -- the defendant had recorded some of his exploits and uploaded them to YouTube -- but contended he was more computer nerd and braggart than a cyber saboteur.

McGraw has been in custody since June.

To see more of The Dallas Morning News, or to subscribe to the newspaper, go to http://www.dallasnews.com. Copyright (c) 2009, The Dallas Morning News Distributed by McClatchy-Tribune Information Services. For reprints, email tmsreprints@permissionsgroup.com, call 800-374-7985 or 847-635-6550, send a fax to 847-635-6968, or write to The Permissions Group Inc., 1247 Milwaukee Ave., Suite 303, Glenview, IL 60025, USA.

Copyright of **Dallas Morning News, The (TX)** is the property of Dallas Morning News, The (TX). The copyright in an individual article may be maintained by the author in certain cases. Content may not be copied or emailed to multiple sites or posted to a listserv without the copyright holder's express written permission. However, users may print, download, or email articles for individual use.
Source: Dallas Morning News, The (TX), Jul 23, 2009
Item: 2W63491596427
~~~~~~~~

Record: 1

England, Spain, Russia, other countries reeling from ransomware attack
By: Robbins, Gary. San Diego Union-Tribune, The (CA). 05/12/2017.
(*AN: 2W61781937072*)

Database: Points of View Reference Center

England, Spain, Russia, other countries reeling from ransomware attack

~~~~~~~~
Gary Robbins

May 12--The scourge of ransomware spread deeper across the globe Friday as at least 12 nations reported that hackers had commandeered computers everywhere from hospitals to telecommunications systems.

The malicious software, known as "WannaCry", demanded that victims pay a small fine to get their computers unlocked.

The attack by unnamed hackers paralyzed hospitals across England, forcing doctors to delay treatment. The malware also disrupted service by Spain's Telefonica, and Russia's MegaFon, a mobile phone operator.

The malware, which was spread by email, also reportedly showed up on computers in the U.S.

"This is the fast-growing ransomware attack that we have ever seen," said Stephen Cobb, a senior researcher in the San Diego office of ESET, a security firm.

The most high-profile attack surfaced early Friday when Britain's National Health Service (NHS) reported that at least 16 of its hospitals had received ransomware, which takes control of a person's computer.

The user then receives a message saying that they must pay a ransom for control to be restored. In most cases, the hacker asks for about $300, usually payable in bitcoin, an international digital currency.

Some NHS patients had to be moved, and others were told that they'd have to wait for health care.

The attack highlighted a growing worldwide problem.

The FBI estimates that ransomware victims paid about $1 billion in ransom worldwide in 2016, and that the figure could double this year. The problem is growing because hackers can buy the software needed for the attack, and victims usually pay.

Friday's attack on England's NHS represents a much larger version of the ransomware assaults that have been occurring at hospitals and health-care systems across the U.S.

"So far, the incidents in the U.S. have mostly happened at smaller hospitals who pay the ransomware, and it doesn't make it into the mainstream media" in terms of coverage and thereby public awareness, said Paul Calatayud, chief technology officer at Overland Park, Kansas-based FireMon.

The victims include California's Hollywood Presbyterian Medical Center, which paid $17,000 in ransom last year to regain control of its computer systems.
~~~~~~~~

"It's plausible that it could happen at larger, corporate health system. The health-care industry is being forced to adopt digital records to reduce costs, and they haven't been moving at the same pace as some other industries," he added.

Britain's NHS confirmed that hospitals were hit by an apparent "ransomware" attack, but there was no immediate evidence that patient data had been accessed.

NHS Digital, which oversees hospital cybersecurity, says the attack used the Wanna Decrypto variant of malware, which infects and locks computers while the attackers demand a ransom.

Pictures posted on social media showed screens of NHS computers with images demanding payment of $300 worth of the online currency Bitcoin, saying: "Ooops, your files have been encrypted!"

(This story is developing. Please check back as we add more details.)

The Associated Press contributed to this story.

Twitter: @grobbins

gary.robbins@sduniontribune.com

___ (c)2017 The San Diego Union-Tribune Visit The San Diego Union-Tribune at www.sandiegouniontribune.com Distributed by Tribune Content Agency, LLC.

Copyright of **San Diego Union-Tribune, The (CA)** is the property of San Diego Union-Tribune, The (CA). The copyright in an individual article may be maintained by the author in certain cases. Content may not be copied or emailed to multiple sites or posted to a listserv without the copyright holder's express written permission. However, users may print, download, or email articles for individual use.

Source: San Diego Union-Tribune, The (CA), May 12, 2017
Item: 2W61781937072

Record: 1

Title: All of records erased, doctor's office closes after ransomware attack

Authors: Carlson, Joe

Source: Star Tribune (Minneapolis, MN). 04/07/2019.

Document Type: Article

Accession Number: 2W63238645138

Database: Points of View Reference Center

All of records erased, doctor's office closes after ransomware attack

~~~~~~~~

Joe Carlson

April 06--A computer virus recently injected itself into the electronic medical record system of Brookside ENT & Hearing Services and ruined the business.

The two-doctor medical practice in Michigan has apparently become the first health care provider in the nation to shut its doors for good because of a ransomware attack, according to half a dozen cybersecurity experts contacted in the past week. Hackers are targeting Minnesota hospitals and clinics at an escalating pace, including four breaches involving patient files already reported in 2019, though any interruptions of work have been temporary.

Ransomware, which encrypts sensitive information and then demands a small financial payment to unlock the files, has become the most common form of malicious software affecting businesses, typically arriving via e-mail, Verizon's 2018 data-breach report says.

Brian Stevenson, president of Roseville cyber security firm FocusPoint Technologies, said about one-third of ransomware victims who pay the ransoms end up getting their data back. Yet, "people are paying the ransoms behind closed doors quite often, because the cost of not being operational for days is worse than the cost of paying," he said.

At Brookside ENT in Battle Creek, Mich., the ransomware virus started by deleting and overwriting every medical record, bill and appointment, including the backups. The virus left behind a duplicate of the deleted files, which could be unlocked with a password that the attacker promised to provide for $6,500 in U.S. currency wired to an account, doctors at the clinic said.

The practice's two ENT surgeons -- Dr. William Scalf, 64, and Michigan state senator Dr. John Bizon, 66 -- refused to pay the attacker's ransom. Scalf said in an interview that there was no guarantee the password would work, or that the malware wouldn't crop up again.

Scalf said an "IT guy" advising them on the attack determined that the attacker did not view the medical records, so the infection wasn't formally reported as a breach under the federal HIPAA law. But lacking any medical and billing records, the doctors closed the business on April 1 and retired about a year before they planned to.
~~~~~~~~

But there was no way to communicate that to patients. "We didn't even know who had an appointment in order to cancel them," Scalf said. "So what I did was just sort of sat in the office and saw whoever showed up. For the next couple of weeks."

Local resident Ann Ouellette, whose teen daughter's records were lost in the attack, told west Michigan CBS affiliate WWMT that her daughter came down with a sinus infection a month after getting surgery and now needs to find a new provider for her follow-up care. Past hearing-test results were lost in the attack, too.

Six cybersecurity researchers and consultants contacted by the Star Tribune said this appears to be the first time this type of scenario has played out in the U.S.

"This is the first time I've heard of a practice shutting down because of ransomware," cybersecurity researcher Billy Rios said via e-mail. Rios, founder of security firm WhiteScope and a well-known critic of lax security in health care products, said some of the medical data might still be recoverable, but it's impossible to tell without access to the infected system.

Beau Woods, a leader with the I Am the Cavalry cybersecurity initiative, said in an e-mail that the majority of small businesses are underprepared for ransomware threats, including many health care delivery organizations. Unlike larger organizations, many smaller providers have no full-time IT employee, let alone a cybersecurity specialist.

"Without better security capabilities and awareness, we can expect to see more frequent, more impactful ransomware incidents impacting health care," Woods wrote.

The digital barrage of attacks against small business IT systems is not limited to health care. But doctors and hospitals hold information of unique value -- your personal medical records.

Already in 2019, four health care providers in Minnesota have reported breaches of patients' personal health information to the U.S. Health and Human Services Department, including a malware attack at a Woodbury reproductive medicine clinic affecting 40,000 patients -- the second-largest health records exposure in Minnesota since reporting began in 2010, federal records show.

Other patient-data breaches reported in the state in the first quarter of 2019 included hacking and e-mail phishing at a behavioral health clinic in the Duluth area (1,200 records), a Catholic-run hospital in Baudette (885 records), and a community hospital district in Blue Earth (2,143 records), federal records show.

Those totals put Minnesota on track to exceed the 10 health data breaches recorded in 2018. The largest breach last year affected 20,800 records following an e-mail hacking incident at the Minnesota Department of Human Services.

The largest reported health care data breach in Minnesota ever was the theft of a laptop owned by medical suppliers Empi and DJO LLC, containing 160,000 medical records, reported in August 2015. Minnesota's most infamous health care data breach -- the 2011 theft a laptop from billing consultant Accretive -- involved the unencrypted medical records of 14,623 Fairview Health Services patients.

For all of the reported incidents, security researchers say there are many other cases in which providers are quietly paying the ransoms to unlock their files without any public notification.

"The reality is that many victims are paying ransom and successfully recovering as a result. Ransomware is a proven successful business model for attackers, complete with customer service to facilitate payments," said Justine Bone, CEO of the med-tech cybersecurity research firm MedSec, via e-mail.

Todd Carpenter, chief engineer at Minneapolis cyber security firm Adventium Labs, said he applauded the owners of Brookside ENT for refusing to pay the $6,500 ransom.

"Much better than paying the ransom, pretending it didn't happen, muddling through -- which some hospitals and clinics have done," Carpenter said.

The attack on Brookside ENT was reported to the FBI, Scalf said. Scalf wasn't optimistic that the investigation would result in charges, but Carpenter said ransomware attacks should be reported to the FBI immediately. The next step would be to get a reputable "data forensics" specialist to review the files, Carpenter said.

The fact that Brookside ENT's backup files were corrupted underscores the importance of keeping additional data backups that are kept offline, away from attacks that can spread over a network.

The Healthcare Sector Coordinating Council published a detailed guidebook last year listing ways to improve what it called "cyber hygiene" in health care settings, including detailed guidance for making e-mail more secure, protecting networks with antivirus protections, limiting network access and actively looking for vulnerabilities that can be addressed.

Or as Bone put it: "Consumers and businesses both large and small need to either make a security investment up front, or manage risk by stashing that ransom for a rainy day. What will get even more interesting is when cyber risk insurers respond to these situations by recommending or making payments on behalf of their clients. It would not surprise me if this were already happening, unfortunately."

Joe Carlson � 612-673-4779

___ (c)2019 the Star Tribune (Minneapolis) Visit the Star Tribune (Minneapolis) at www.startribune.com Distributed by Tribune Content Agency, LLC.

Copyright of **Star Tribune (Minneapolis, MN)** is the property of Star Tribune (Minneapolis, MN). The copyright in an individual article may be maintained by the author in certain cases. Content may not be copied or emailed to multiple sites or posted to a listserv without the copyright holder's express written permission. However, users may print, download, or email articles for individual use.
Source: Star Tribune (Minneapolis, MN), Apr 07, 2019
Item: 2W63238645138

Cyberattack on top Indian hospital highlights security risk - The leading hospital in India's capital is limping back to normalcy after a cyberattack crippled its operations for nearly two weeks

December 7, 2022 | Independent, The/The Independent on Sunday: Web Edition Articles (London, England)
Author/Byline: Aniruddha Ghosal
490 Words

The leading hospital in India's capital limped back to normalcy on Wednesday after a cyberattack crippled its operations for nearly two weeks.

Online registration of patients resumed Tuesday after the hospital was able to access its server and recover lost data. The hospital worked with federal authorities to restore the system and strengthen its defenses.

It's unclear who conducted the Nov. 23 attack on the All India Institute of Medical Sciences or where it originated. Hospital authorities didn't respond to requests for comment.

The attack was followed by a series of failed attempts to hack India's top medical research organization, the Indian Council of Medical Research. This raised further concerns about the vulnerability of India's health system to attacks at a time when the government is pushing hospitals to digitize their records.

More than 173,000 hospitals have registered with a federal program to digitize health records since its launch in September 2021. The program assigns patients numbers that are linked to medical information stored by hospitals on their own servers or in cloud-based storage. Experts fear that hospitals may not have the expertise to ensure digital security.

“Digitizing an entire health care system without really safeguarding it can pretty much kill an entire hospital. It suddenly stops functioning,” said Srinivas Kodali, a researcher with the Free Software Movement of India.

That is what happened to the hospital in New Delhi. Healthcare workers couldn't access patient reports because the servers that store laboratory data and patient records had been hacked and corrupted.

The hospital normally treats thousands of people a day, many of whom travel from distant places to access affordable care. Always crowded, queues at the hospital grew even longer and more chaotic.

“The entire system isn't working because of the hack,” said Deep Ranjan, who came to New Delhi from northeastern Assam state. He said he had spent five days waiting in line and still has not seen a doctor.

Sandeep Kumar, who accompanied his ill father, said the digital attack meant that appointments couldn't be

booked online, and that doctors could do little when they saw patients because they couldn't access their medical history.

“We are digitizing (everything), but then there is an attack on the country's most important medical institute,” he said.

On Nov. 30, there were repeated but ultimately unsuccessful attempts to breach the website of the Indian Council of Medical Research, the Press Trust of India news agency reported.

The attack on the hospital raised “serious questions about the cybersecurity of the country,” said K.C. Venugopal, a member of Parliament from the main opposition Congress party.

India drafted a proposed law governing data privacy last month, but critics say it offers few safeguards to people. It has not yet been passed by Parliament.

___

Associated Press journalist Piyush Nagpal contributed to this report.

___

The Associated Press Health and Science Department receives support from the Howard Hughes Medical Institute’s Science and Educational Media Group. The AP is solely responsible for all content.

© Copyright 2022 Independent News & Media Ltd., All Rights Reserved.

Record: 1

Cyberattack concerns Humber MDs. By: Megan Ogilvie Toronto Star. Toronto Star (Canada). 06/19/2021. Abstract: A group of emergency physicians at Humber River Hospital has written a letter to the hospital's administrators calling for the emergency department to be temporarily closed until IT systems are fully restored, citing concerns over patient safety. The hospital's information technology system has been shut down since Monday following an early-morning ransomware attack that triggered a Code Grey, or loss of essential services. [ABSTRACT FROM PUBLISHER] (*AN: 6FPTS2021061961297286*)

Database: Points of View Reference Center

Cyberattack concerns Humber MDs.

Hospital says it's seeing 200 patients a day with only one complaint

A group of emergency physicians at Humber River Hospital has written a letter to the hospital's administrators calling for the emergency department to be temporarily closed until IT systems are fully restored, citing concerns over patient safety.

The hospital's information technology system has been shut down since Monday following an early-morning ransomware attack that triggered a Code Grey, or loss of essential services.

The IT shutdown has led to delays in medical and diagnostic test results needed to assess patients, according to the physicians who wrote the letter obtained by the Star. "This is leading to compromised and dangerous conditions for our patients," the letter reads.

The authors want "normal activities in the emergency department to cease until our IT systems are restored to normal function," noting patients could travel to nearby GTA hospitals that "can operate at the proper level of care."

The letter, sent late Thursday to administrators at Humber River, was co-authored by a group of emergency physicians, none of whom signed their name to the letter due to concerns about speaking against their employer. Staff who spoke with the Star say the letter was approved by a group of 30 physicians who work in the hospital's emergency department. That's about half the staff of 65 physicians in the department.

In an interview with the Star, Dr. Leon Rivlin, chief and medical director of the emergency department, said the hospital has "continued to deliver care to all of our patients in a very safe way" during the ongoing Code Grey.

The emergency department is now safely relying on paper records while assessing patients, he said. It's also working closely with the diagnostic and laboratory departments "to ensure that we are maintaining all of the efficiencies and the safety mechanisms that have been developed in order to make sure patients get the care that they need," Rivlin said.

Since Monday, Rivlin said he has been made aware of only one patient complaint regarding care received in the emergency department.

"And we're seeing between 200 and 300 patients a day," he said.

In their letter, the group of physicians provide examples of how they believe patient care has been compromised during the IT shutdown, including: delays in laboratory tests, including those for suspected heart attacks; the inability to view and share diagnostic test results, including those needed to assess a trauma patient; and lengthy waits for blood test results for dialysis patients.

The letter goes on to say: "The situation is currently such that we would never consider sending our own loved ones here."

Copyright of **Toronto Star (Canada)** is the property of Toronto Star. The copyright in an individual article may be maintained by the author in certain cases. Content may not be copied or emailed to multiple sites or posted to a listserv without the copyright holder's express written permission. However, users may print, download, or email articles for individual use.
Source: Toronto Star (Canada), 06/19/2021
Item: 6FPTS2021061961297286

Record: 1

Cyberattack brings down Humber River Hospital computers. By: Maria Sarrouh Toronto Star Megan Ogilvie Toronto Star. Toronto Star (Canada). 06/16/2021. Abstract: A cyberattack at Humber River Hospital has triggered a Code Grey - or loss of essential services - leaving staff unable to access electronic patient records and diagnostic test results, and leading to long waits in the busy emergency department. The hospital's information technology system was affected around 2 a.m. Monday after it experienced a ransomware attack. In a statement posted online Tuesday afternoon, the hospital said "no confidential information was released" and the attack was "discovered almost immediately." All of its IT systems were shut down, including the one for patient health records. [ABSTRACT FROM PUBLISHER] (*AN: 6FPTS2021061661262310*)

Database: Points of View Reference Center

Cyberattack brings down Humber River Hospital computers.

Network shutdown has led to long waits in ER, diagnostic testing

A cyberattack at Humber River Hospital has triggered a Code Grey - or loss of essential services - leaving staff unable to access electronic patient records and diagnostic test results, and leading to long waits in the busy emergency department.

The hospital's information technology system was affected around 2 a.m. Monday after it experienced a ransomware attack. In a statement posted online Tuesday afternoon, the hospital said "no confidential information was released" and the attack was "discovered almost immediately." All of its IT systems were shut down, including the one for patient health records.

"Ransomware usually encrypts files and then, once most are encrypted, asks for ransom. Since we shut down quickly, encryption is not an issue, although we are dealing with some corrupt files," the statement reads.

Hospital staff told the Star the network shutdown affected patient care by leading to long waits in the emergency department.

It has also created long wait times for diagnostic tests, including those for suspected heart attacks, sources said.

The hospital has cancelled a variety of clinics and has staff at its main doors to redirect patients. So far, surgeries are not affected and the emergency department remains open, though some ambulances will be redirected to other hospitals, the statement said.

Melissa Granados, a patient diagnosed with a uterine fibroid (tumour), said she arrived at the hospital's main emergency room at 3:15 p.m. Monday, with heavy bleeding.

After two-and-a-half hours of waiting, Granados said a hospital manager informed patients of the system failure. She wasn't able to see a doctor until six to seven hours after she initially sought help, and didn't leave the hospital until 1 a.m. Tuesday. Granados said she felt frustrated when a nurse told her the failure had started in the morning, but patients weren't informed earlier.

"If they cared enough about their patients, they would have told us when we walked in, 'Our systems are down, we suggest you go into another emergency department because our waits are very long,'" Granados said.

Although she's dealt with heavy periods and spotting for the past month-and-a-half, the bleeding she experienced Monday was extreme. Her primary care doctor works at St. Michael's Hospital, but she opted to seek help at Humber River Hospital's emergency department instead, because it's closer to her home around Jane Street and Wilson Avenue.

"I was seeing black and I was going to pass out," Granados said. "I told them, I'm bleeding out."

Granados said she asked hospital staff to transfer her to another hospital, but was told to wait until a doctor could see her.

Humber River Hospital said its IT department is working with an external recovery firm to get its systems back up and running. More than 5,000 computers (800 of which are servers) will be restarted manually. The hospital said in its Tuesday afternoon statement that systems will be brought back online in a staggered approach over the next 48 hours.

Ontario Health said they were informed on Monday morning that Humber River Hospital's systems were down. They said the situation at the hospital is being monitored closely and Ontario Health's security team has been supporting the hospital as needed.

Christopher Parsons, a senior research associate at the University of Toronto's Citizen Lab at the Munk School of Global Affairs and Public Policy, said Humber River Hospital appears to have had appropriate safeguards in place and enacted a quick and accurate response to the ransomware attack.

"If you take their statement at face value, what they did is impressive," he said, noting it's generally uncommon for large and mid-size organizations to regularly update their systems to quickly discover attacks. In its statement, the hospital said its "most recent patching" took place on Sunday.

Rapidly shutting down and disconnecting from the existing system - what the hospital indicated it did in its statement - is also key to prevent the attack from spreading, Parsons said.

In the case of a health-care facility, shutting down the entire system to halt the attack means critical network services, such as electronic patient records and diagnostic imaging, are also paralyzed.

Parsons said hospitals and health-care systems are being targeted around the world, and pointed to a recent string of such ransomware attacks, including one in May that crippled Ireland's public health system. In many cases, targeted hospitals were forced to cancel non-urgent procedures and staff had to use pen and paper to track patient records.

In 2019, several Ontario hospitals were affected by ransomware viruses, including Michael Garron Hospital in Toronto. That same year, LifeLabs, the country's largest medical diagnostic testing company, revealed it had paid a ransom to secure data - including the personal information of millions of customers - after a cyberattack.

In addition to having strong network security precautions, Parsons suggests hospitals and health-care facilities have rigorous backup plans so they can provide safe and adequate patient care after a cyberattack. He said he hopes Humber River Hospital will provide more details so other hospitals and organizations can learn from its experience.

"From where I sit, ransomware is the equivalent of a new and virulent disease," he said. "It's only going to get worse."

Humber River Hospital said confidential patient information wasn't leaked during a cyberattack on Monday.Justin Greaves Metroland File Photo

---

Copyright of **Toronto Star (Canada)** is the property of Toronto Star. The copyright in an individual article may be maintained by the author in certain cases. Content may not be copied or emailed to multiple sites or posted to a listserv without the copyright holder's express written permission. However, users may print, download, or email articles for individual use.

Source: Toronto Star (Canada), 06/16/2021
Item: 6FPTS2021061661262310

Record: 1

Crozer Health's computer system were offline Thursday morning By: Brubaker, Harold. Philadelphia Inquirer, The (PA). 08/03/2023. (*AN: 2W62184530620*)

Database: Points of View Reference Center

Crozer Health's computer system were offline Thursday morning

~~~~~~~~

Harold Brubaker

Aug. 3—Computer systems at Delaware County's Crozer Health were down Thursday morning, according to five employees and others familiar with the system's operations.

Details on what caused the outage were not available. Crozer spokesperson Lori Bookbinder did not immediately respond to messages asking for more information.

Crozer, which is owned by Los Angeles-based Prospect Medical Holdings Inc., includes Crozer-Chester Medical Center in Upland and Taylor Hospital in Ridley Park. Prospect ended inpatient services at Delaware County Memorial Hospital in Drexel Hill and Springfield Hospital in Springfield.

In June 2020, Crozer was the victim of a malware attack. The organization said at the time that it had quickly isolated the problem, but trade publications that cover cybersecurity said some Crozer data was put up for auction after Crozer declined to pay ransom.

The health care sector is the most popular target for cyberattacks and the sector lags other is cybersecurity measures, Moody's Investor Service said in an April report. The industry is considered a rich target because its data systems contain personal information like names and addresses, ages, and social security numbers.

The Philadelphia Inquirer suffered a cyberattack in May that prevented the publication of the outlet's Sunday, May 14, print edition.

This story will be updated as more information becomes available.

___ (c)2023 The Philadelphia Inquirer Visit The Philadelphia Inquirer at www.inquirer.com Distributed by Tribune Content Agency, LLC.

Copyright of **Philadelphia Inquirer, The (PA)** is the property of Philadelphia Inquirer, The (PA). The copyright in an individual article may be maintained by the author in certain cases. Content may not be copied or emailed to multiple sites or posted to a listserv without the copyright holder's express written permission. However, users may print, download, or email articles for individual use.

Source: Philadelphia Inquirer, The (PA), Aug 03, 2023

Item: 2W62184530620
~~~~~~~~

Record: 1

Cheyenne Regional payroll impacted by ransomware attack By: Black, Hannah. Wyoming Tribune-Eagle (Cheyenne, WY). 03/27/2022. (*AN: 2W62527990225*)

Database: Points of View Reference Center

Cheyenne Regional payroll impacted by ransomware attack

~~~~~~~~
Hannah Black

Mar. 27—CHEYENNE — More than 2,000 employees were affected when the software that Cheyenne Regional Medical Center and its overall health system uses for timekeeping and processing payroll was targeted by a larger scale phishing ransomware attack, the Wyoming Tribune Eagle has learned.

This incident apparently upset some CRMC employees, and it caused some to be overpaid and to have to later reimburse their employer for money they were incorrectly paid that was not really owed to the staffers. Others meanwhile were underpaid, and the hospital was making good on their full paychecks. Some of the systems that CRMC uses for human resources and related issues were down for several months, as the software vendor worked to fully fix all of its systems.

Software company Kronos' workforce management system, Kronos Private Cloud, went down on Dec. 11. This KPC outage affected 15,000 employers in the U.S. and worldwide, according to a written statement from Cheyenne Regional's Joanna Vilos, its chief human resources officer.

After the payroll software the health system relies on went dark, multiple departments "worked tirelessly to manually input data and ensure that our employees would continue to receive a paycheck," Cheyenne Regional said in a previous statement.

"Cheyenne Regional wants to thank everyone in these departments for all they've done to work through this difficult situation. We also want to thank our employees for their patience and understanding during this time," the statement continued.

While Kronos was down, Vilos said, the health system's payroll department manually processed paychecks for its employees over five pay cycles.

Kronos again became fully functional in early March, the statement said. When Cheyenne Regional could access the payroll system, it "immediately began reconciling all employees' paychecks," Vilos said.

Vilos said about 55% of employees were overpaid, while about 45% were underpaid.

"Cheyenne Regional has corrected all the underpayments, and employees have been given several payback options to correct the overpayments, including repaying Cheyenne Regional over an extended period of time," she continued. "We believe our system has been restored to accuracy for purposes of benefits, taxes and overall compensation, but we encourage employees to schedule an appointment with our payroll team if they have any questions or concerns about their payroll information."
~~~~~~~~

No personal employee information was compromised in the attack, Vilos said, thanks to CRMC's "robust set of policies and practices against cyberattacks."

"We are committed to doing all we can to prevent this from happening again," the health system's statement said.

Ransomware

Vilos wrote that it was Cheyenne Regional's understanding that Kronos has "worked diligently to further augment their security."

In an update earlier this month to a website about the ransomware incident, Kronos said that "the first phase of our restoration process was completed on January 22." This restored to customers (such as the local hospital system) the "core functionality — namely, time, scheduling, and HR/payroll capabilities," according to the company. "Since that time, our team has been diligently focused on restoring the additional applications that some of our KPC customers use."

In an email to the WTE Saturday, a spokesperson for UKG, which appears to be the owner of Kronos, noted that the core functionality had indeed been restored by Jan. 22. "In light of the global pandemic, we had specialist teams dedicated to healthcare, first responders, and similar customers," per the representative. "Since the incident occurred, we have focused on communicating with all of our customers in a transparent, timely manner."

Cheyenne Regional did not respond to a question about whether any employees had threatened legal action because of over- or underpayments. UKG/Kronos did not address specific questions about CRMC that were sent to Kronos.

At least two health systems, Scripps Health in San Diego and UMass Memorial in Massachusetts, are facing lawsuits related to the Kronos attack.

Phishing is when a perpetrator uses an email or text message to trick someone into revealing sensitive information, or to click on a link or open an attachment that can deploy malicious software, such as ransomware.

Ransomware attacks are "pretty common," said Mike Borowczak, director of the University of Wyoming's Cybersecurity Education and Research Center.

Borowczak said the goal of ransomware attacks are usually to collect a ransom by taking down a system.

"The idea is, if I'm an attacker, I'm going to get into your system somehow, I'm going to do something malicious that is reversible, but makes it impossible for you to do your job or to provide the service you normally provide," he said.

If the victim of the attack pays the requested money, the perpetrator may give that person or organization the tools to reverse the damage or unlock affected systems.

"They're holding your information, your data, your systems hostage for capital gain," Borowczak said.

Although ransomware attacks can be perpetrated by anyone, attacks on large operations are typically conducted by organized crime groups or, in some cases, heavily sanctioned nation states that need a way to make money, the cybersecurity expert said.

Kronos said in early March that its investigation had been completed, but the source of the attack was unclear.

Risk

Third-party payroll systems are convenient for many companies. Paying for these services, which operate through the internet and on the payroll management company's servers, means businesses don't have to have special equipment within their own facilities to take care of payroll and timekeeping, Borowczak said.

But online systems pose an inherent risk — one demonstrated by the recent attack on Kronos.

Kronos handles "a massive percentage of corporate payroll management systems," Borowczak said. According to NPR, about 8 million total employees were affected, including big companies like FedEx, PepsiCo and Amazon's Whole Foods, as well as some public employers.

Although a ransomware attack was the cause of the recent Kronos outage, the cybersecurity expert said it's just one of many things that could cause such a system to go down for an extended period.

Many organizations can't incur the cost of having duplicate systems for things like payroll, Borowczak said. When such a vital service is taken offline, most companies have to revert to manually managing timekeeping and employee paychecks.

"The ultimate concern here is that the hospital and many others relied on a cloud service that became unavailable," he said. "There's a lot of different reasons why service can be disrupted. (What matters is how) you respond to that disruption as the end company that is utilizing a cloud service, or any service that's remote."

Chief Human Resources Officer Vilos said Kronos notified Cheyenne Regional "promptly" of the ransomware attack and the resulting outage of its payroll and timekeeping services. She said employees were then notified that it could remain inaccessible for "several weeks," and that "we would be initiating our contingency plan to ensure employees would continue to be paid."

Cheyenne Regional does have cyber insurance, Vilos said. However, this insurance could only be used if the hospital was the direct target of an attack, rather than a secondary victim because an external service — in this case, Kronos — was targeted.

Eric Boley, president of the Wyoming Hospital Association, said he was not aware of any other medical facilities in the state having been affected by the Kronos hack. Other hospitals have fallen victim to ransomware and phishing attacks in the past few years, he said, but to his knowledge, "this is the first type of attack on this particular type of software."

While Kronos holds some responsibility for not being able to offer their promised services, the ultimate responsibility of continuing payroll functions in these situations falls to the employer, Borowczak said.

According to Boley, medical facilities around the state use "all types of cybersecurity safeguards." But "attacks continue to come daily," he said.

"We hear from the feds that it is not an issue of if a facility will be attacked," Boley said, "but when."

Hannah Black is the Wyoming Tribune Eagle's criminal justice reporter. She can be reached at hblack@wyomingnews.com or 307-633-3128. Follow her on Twitter at @hannahcblack.

___ (c)2022 Wyoming Tribune-Eagle (Cheyenne, Wyo.) Visit Wyoming Tribune-Eagle (Cheyenne, Wyo.) at www.wyomingnews.com Distributed by Tribune Content Agency, LLC.

Copyright of **Wyoming Tribune-Eagle (Cheyenne, WY)** is the property of Wyoming Tribune-Eagle (Cheyenne, WY). The copyright in an individual article may be maintained by the author in certain cases. Content may not be copied or emailed to multiple sites or posted to a listserv without the copyright holder's express written permission. However, users may print, download, or email articles for individual use.
Source: Wyoming Tribune-Eagle (Cheyenne, WY), Mar 27, 2022
Item: 2W62527990225

Record: 1

Chemotherapy patients sent home By: Rebecca Myers. Sunday Times, The. 05/14/2017, p4-4. 1. Abstract: Tom Griffiths, a 39-year-old father of two, was receiving chemotherapy for advanced bowel cancer at St Bartholomew's Hospital in London when the terminal next to him displaying his blood records was suddenly overrun by the ransomware. [ABSTRACT FROM PUBLISHER] (*AN: 7EH125468621*)

Database: Newspaper Source

Chemotherapy patients sent home

Section: News Edition: 01

Tom Griffiths, a 39-year-old father of two, was receiving chemotherapy for advanced bowel cancer at St Bartholomew's Hospital in London when the terminal next to him displaying his blood records was suddenly overrun by the ransomware.

"A screen popped up on the computer with a padlock," he said.

"Because I'd already got my blood results, it was safe for me to have my chemotherapy — but the patients who had arrived after me were sent home.

"Patients had made the effort to come into hospital, stressful anyway if you're ill. Chemotherapy is quite an ordeal, not just physically but psychologically. To then have it postponed and to go through that all over again can be quite traumatic."

Photo Captions
Griffiths: cancer patient

Source: Sunday Times, The, May 14, 2017, p4, 1p
Item: 7EH125468621

Record: 1

British Hospitals Among Targets Of Global Ransomware Attack. By: Frank Langfitt. All Things Considered (NPR). 05/12/2017. (*AN: 6XN201705122121*)

Database: Newspaper Source

British Hospitals Among Targets Of Global Ransomware Attack.

AUDIE CORNISH: Cyber extortion attacks spread across the world today. They hit organizations ranging from a telecom giant in Spain to the National Health Service in England. The attacks used ransomware, which demands payment before allowing users to access their own data again. NPR's Frank Langfitt begins our coverage from London.

FRANK LANGFITT: The ransomware attack struck more than 30 facilities in England's vaunted National Health Service, or NHS, forcing some hospitals and clinics to shut down their computer systems to prevent the malware from spreading. Physicians had to return to working with pen and paper. Hospitals told patients not to come to emergency centers unless their condition was urgent. Malware typically gets into a computer when someone clicks on an infected attachment. Craig Williams, a cybersecurity specialist with Cisco Talos, the firm's threat intelligence unit, explains how ransomware generally operates from there.

CRAIG WILLIAMS: It searches your hard drive for valuable file types - so things like word docs, spreadsheets, pictures, things like that. And then it takes those files and encrypts them so that it can hold them for ransom.

LANGFITT: Williams says the ransomware that infected the NHS, which the Government Health Service said was called Wanna Decrypter, doesn't require a user to click an attachment. The malicious code can just worm its way into a computer system without human prompting.

WILLIAMS: This one's significantly worse. You could just walk up to your computer, and it's infected even if you didn't even touch it.

LAWRENCE JONES: They're happening every minute of every day, and that's globally.

LANGFITT: Lawrence Jones says ransomware attacks have become increasingly common. Jones is CEO of UKFast, which provides web hosting and cybersecurity for thousands of clients, including parts of the NHS. He says ransomware is a business that operates on volume. For instance, the attacks on the NHS asked users to pay the equivalent of about $300 to get access to their data.

JONES: We see people asking for quite small amounts of money in relation to the damage that they're threatening. So it's easier in a situation like this just to pay the ransom. There's nothing really you can ever do. You've given permission to people to lock down your systems, and you're never going to get that data back. So you have to pay that money or retrieve from previous backups you may have.

LANGFITT: Jones says the people behind ransomware attacks can range from sophisticated criminal gangs to teenagers, and they're very hard to catch. Frank Langfitt, NPR News, London.

Copyright of **All Things Considered (NPR)** is the property of National Public Radio, Inc. The copyright in an individual article may be maintained by the author in certain cases. Content may not be copied or emailed to multiple sites or posted to a listserv without the copyright holder's express written permission. However, users may print, download, or email articles for individual use.

Source: All Things Considered (NPR), 05/12/2017
Item: 6XN201705122121

Record: 1

Cyberattack hits major hospital in Spanish city of Barcelona By: The Associated Press. AP Financial News, 03/06/2023; Abstract: MADRID (AP) — A ransomware cyberattack on one of Barcelona' s main hospitals has crippled the center's computer system and forced the cancellation of 150 nonurgent operations and up to 3,000 patient checkups, officials said Monday.The attack Sunday on the Hospital Clinic de Barcelona shut down computers at the facility's laboratories, emergency room and pharmacy at three main centers and several external clinics. [ABSTRACT FROM PUBLISHER] (*AN AP37e0fee33798c56459e63866ca8b449f*)

Database: Newswires

Cyberattack hits major hospital in Spanish city of Barcelona

~~~~~~~~

By The Associated Press

MADRID (AP) — A ransomware cyberattack on one of Barcelona' s main hospitals has crippled the center's computer system and forced the cancellation of 150 nonurgent operations and up to 3,000 patient checkups, officials said Monday.

The attack Sunday on the Hospital Clinic de Barcelona shut down computers at the facility's laboratories, emergency room and pharmacy at three main centers and several external clinics.

"We can't make any prediction as to when the system will be back up to normal," hospital director Antoni Castells told a news conference on Monday. He said the hospital's contingency plan would allow them to function for several days, but he hoped the system would be fixed sooner.

A Catalonia regional government statement said the region's Cybersecurity Agency was working to restore the system. The agency said Monday the attack was orchestrated from outside of Spain by a group called "Ransom House."

Regional government telecommunications secretary Segi Marcén said that hackers hadn't made any ransom demand so far but that no money would be paid.

The hospital's press department said that all written work was being done on paper and that the hospital was diverting new urgent cases to other hospitals in the city. Spanish state news agency EFE said the attack cut off access to patients records and communication between units.

Copyright of **Copyright 2023 The Associated Press. All rights reserved. This material may not be published, broadcast, rewritten or redistributed without permission.** is the property of Associated Press DBA Press Association. The copyright in an individual article may be maintained by the author in certain cases. Content may not be copied or emailed to multiple sites or posted to a listserv without the copyright holder's express written permission. However, users may print, download, or email articles for individual use.
Source: Copyright 2023 The Associated Press. All rights reserved. This material may not be published,
~~~~~~~~

broadcast, rewritten or redistributed without permission.,
Item:

NewsBank inc.

Jury Selection Begins in Controversial Hospital Hacking Case

July 18, 2018 | Newsmax.com
Author: Michael Dorstewitz | Section: America | 380 Words
OpenURL Link

Jury selection begins Thursday in the high-profile hacking case of Martin Gottesfeld, a computer expert and human rights activist accused of involvement in a denial of service attack launched against a Boston hospital.

The case, to be tried in federal District Court in the Massachusetts district, stems from a 2014 Internet attack that disrupted the website of Boston Children's Hospital. The attack was apparently launched to protest the treatment of a Connecticut teenager, Justina Pelletier, who received conflicting medical diagnoses, had been hospitalized, and was in the custody of the state for well over a year.

Pelletier's case drew national attention. An in-depth The Boston Globe investigation of her case in December 2013 found, over the course of 18 months, there were "at least five cases where a disputed medical diagnosis led to parents either losing custody or being threatened with that extreme measure."

The actual trial is scheduled to get underway Monday. Gottesfeld could face up to 15 years in prison if convicted.

Earlier this week, a hearing was held on a motion by Gottesfeld's court-appointed public defender, attorney David Grimaldi, to be dismissed from the case. That motion was denied, clearing the way for the trial to proceed.

Gottesfeld allegedly worked with the "hacktivist" group Anonymous to launch the Internet attack on the hospital's website, which occurred during a fundraising drive, disrupting access by other users. Gottesfeld's website, FreeMartyG.com, refers to the disruption as "an online sit-in." But prosecutors say the attack interfered with "online service portals for patients, providers, and physicians."

Martin Gottesfeld's wife, Dana Gottesfeld, has been crusading to draw attention to his case. She states her husband is up against powerful interests in the Boston area, and says the court has blocked her husband's defense team from using Pelletier as a defense.

"This is going to be a show trial," she told Newsmax. "There isn't a real chance for justice here."

A judge has ordered Martin Gottesfeld be held without bond until the case is resolved.

Michael Dorstewitz is a retired lawyer and has been a frequent contributor to BizPac Review and Liberty Unyielding. He is also a former U.S. Merchant Marine officer and an enthusiastic Second Amendment supporter, who can often be found honing his skills at the range. To read more of his reports — Click Here Now.

Copyright (c) 2018 Newsmax.com, All rights reserved.

- **Citation (apa Style)**
  Dorstewitz, M. (2018, July 18). Jury Selection Begins in Controversial Hospital Hacking Case*Newsmax.com*. Available from NewsBank: Access World News: https://infoweb.newsbank.com/apps/news/document-view?p=AWNB&docref=news/16D4110FC215BCB8.

NewsBank inc.

Surgeries delayed as hospital network hacked

October 2, 2019 | Australian, The/Weekend Australian, The/Australian Magazine, The (Australia)
Author: RACHEL BAXENDALE, VICTORIAN POLITICAL REPORTER | Section: TheNation | 372 Words
Page: 5
OpenURL Link

A regional Victorian hospital network has been hacked in a ransomware attack, just months after an Auditor-General's report warned of serious cybersecurity weaknesses in the state's health system.

The Andrews government says patient information is safe, and emergency care will not be affected. However, elective surgery and some outpatient services may be delayed at hospitals in Gippsland and southwest Victoria, which have been forced to revert to manual contact and booking systems.

The Victorian Department of Premier and Cabinet on Tuesday said hospitals that are part of the Gippsland Health Alliance and the South West Alliance of Rural Health had been attacked.

"The Victorian Cyber Incident Response Service has been deployed and worked with impacted health services overnight to respond to the attack," the department said.

"The cyber incident, which was uncovered on Monday, has blocked access to several systems by the infiltration of ransomware, including financial management." The department confirmed a number of servers across the state had been affected, and said investigations were under way to determine the extent of the problem. "At this time, there is no suggestion that personal patient information has been accessed," it said.

Victorian Auditor-General Andrew Greaves revealed in May that he had successfully hacked into the IT systems of some of the state's biggest hospitals and accessed sensitive patient data, highlighting serious cybersecurity weaknesses.

Asked whether more could have been done following the warning to strengthen hospital systems, Premier Daniel Andrews said the system was "much stronger than it's ever been".

"As we see, though, whether it be in the private sector in the public sector, in our jurisdiction, indeed right around the world, systems can be built and can be built very, very strong, but this sort of criminal activity ... Much like a house, you can build a fortress but if people are prepared to put the planning into it, there are still ways to get in, and that's what's occurred overnight," he said.

Hospitals have disconnected many of their systems from the internet to quarantine the hack.

"This isolation has led to the shutdown of some patient record, booking and management systems, which may impact on patient contact and scheduling," the department said."Where practical, hospitals are reverting to manual systems to maintain their services."

Copyright, 2019, Nationwide News Pty Limited

- **Citation (apa Style)**
  BAXENDALE, R. (2019, October 2). Surgeries delayed as hospital network hacked*Australian, The (Australia)*, p. 5. Available from NewsBank: Access World News: https://infoweb.newsbank.com/apps/news/document-view?p=AWNB&docref=news/1764E62040B5CB10.

NewsBank inc.

Los Angeles hospital attack concerns cybersecurity experts

February 19, 2016 | St. Louis Post-Dispatch (MO)
Author: JUSTIN PRITCHARD Associated Press | Section: News | 677 Words
Page: A17
OpenURL Link

LOS ANGELES • Cybersecurity experts worry that the $17,000 a Los Angeles hospital paid hackers to regain control of its computers could signal a troubling escalation of the growing “ransomware” threat.

Though patient care was not “compromised in any way,” Hollywood Presbyterian Medical Center paid the bounty “in the best interest of restoring normal operations,” President Allen Stefanek said in a written statement.

Many ransomware victims pay quietly, or abandon infected machines. It was unusual that Hollywood Presbyterian, which has more than 400 beds and is owned by CHA Medical Center of South Korea, both revealed the attack publicly and disclosed its cost.

Computer security experts said hospitals are particularly vulnerable because some medical equipment runs on old operating systems that cannot easily be safeguarded. If an employee opens an infected file from a computer that also connects with a patient monitoring station or insulin pump, those devices also could be locked.

Hospitals have not been as diligent in combating cyber threats such as ransomware as other sectors, according to several experts, despite the life-and-death nature of their operations, their tight control over patient information and mandates that they move toward electronic record keeping.

Hospitals are “about 10 to 15 years behind the banking industry” in combatting cyber threats, said Lysa Myers, a researcher with the computer security firm ESET.

The math behind whether to pay a ransom demand can be simple.

Paying thousands of dollars to resolve a serious attack that has penetrated a multimillion dollar business such as a large hospital would be “a no brainer,” said James Carder, chief information security officer of LogRhythm, a security intelligence and analytics firm.

Several companies have told Carder that the FBI suggested they pay ransom, he said. Jason Haddix, the director of technical operations at the information security firm Bugcrowd, said companies also had told him the same.

“If you’re at a point where you can’t do anything,” said Haddix, “sometimes the only option is to pay.”

An FBI spokeswoman did not immediately respond when asked whether the FBI has in some cases suggested that a company pay. The agency said it is investigating the Hollywood Presbyterian case.

“Ransomware has been around for several years, but there’s been a definite uptick lately in its use by cyber criminals,” the FBI wrote in a 2015 post on its website. The agency said that it is “targeting these offenders and their scams.”

Hollywood Presbyterian paid 40 bitcoins, a digital currency of floating value that on Thursday was worth about $420 each. The problem was first noticed Feb. 5, hospital president Stefanek said, and its system was fully functioning 10 days later.

One reason hackers are attracted to ransomware is that it can be created with relative ease — do-it-yourself ransomware kits are available — and the return on investment can be strong.

To launch a ransomware campaign that lasts one month might cost $5,900, and generate about $90,000 in revenue, according to projections by the cyber security firm Trustwave.

A report from Intel Corp.'s McAfee Labs released in November said the number of ransomware attacks is expected to grow in 2016 because of increased sophistication in the software used to do it. The company estimates that on average, 3 percent of users with infected machines pay a ransom.

Though a hacker may get several hundred dollars to unlock many individual computers, getting $17,000 is a decent payday. Based on the public confirmation of that figure, hackers are "going to begin to test the price," said Jack Danahy, chief technology officer at cyber security firm Barkly.

Copyright (c) 2016 St. Louis Post-Dispatch

- **Citation (apa Style)**
  PRITCHARD, J. (2016, February 19). Los Angeles hospital attack concerns cybersecurity experts.*St. Louis Post-Dispatch (MO)*, p. A17. Available from NewsBank: Access World News: https://infoweb.newsbank.com/apps/news/document-view?p=AWNB&docref=news/15B27A438DBCA5B8.

NewsBank inc.

North Korean hackers targeting hospitals, healthcare providers, U.S. agencies warn

July 8, 2022 | WorldNetDaily (USA)
Author: WND News Services | Section: Health | 119 Words
OpenURL Link

(UPI) – North Korean-backed hackers are targeting hospitals and other healthcare organizations in the United States with ransomware, a trio of government agencies that includes the Federal Bureau of Investigation warned in a new cybersecurity alert.

The advisory, issued Wednesday, said that North Korean cyber actors have been using "Maui" ransomware in attacks against the healthcare and public health sector since at least May 2021.

The ransomware was reportedly used to encrypt servers responsible for health records, diagnostics and imaging services. Under such attacks, the hackers can demand that victims pay a fee to restore access to the servers.

Read the full story ›

The post North Korean hackers targeting hospitals, healthcare providers, U.S. agencies warn appeared first on WND.

Copyright © 2022 WorldNetDaily, Inc., All rights reserved.

- **Citation (apa Style)**
  News Services, W. (2022, July 8). North Korean hackers targeting hospitals, healthcare providers, U.S. agencies warn. *WorldNetDaily (USA)*. Available from NewsBank: Access World News: https://infoweb.newsbank.com/apps/news/document-view?p=AWNB&docref=news/18B24AB781C8AE28.

NewsBank inc.

AIIMS attack led to new SOP for breaches: Outgoing cyber chief

July 3, 2023 | Hindustan Times (New Delhi, India)
206 Words
OpenURL Link

New Delhi, July 3 -- The ransomware attack on the All-India Institute of Medical Sciences prompted the government to formulate a national cybersecurity response framework (NCRF), India's former national cybersecurity coordinator has said.

Speaking to HT, Lt Gen Rajesh Pant, who took over in 2019 and held the top cybersecurity job till June 30, said the attack shone a spotlight on the need to protect critical infrastructure.

"It was realised that critical sectors need to have a uniform framework to respond to cybersecurity," said Pant. "So, the NCRF was conceptualised. It will be put in the public domain for critical infrastructure, such as those in the power and health sectors to implement."

The framework outlines an architecture of a cyber defence system, he said.

On November 23, the systems at AIIMS and its centres were corrupted by the cyberattack, which wiped outpatient and research data from its primary and backup servers.

Pant said the attack exposed loopholes in the cyber defence systems and several lessons have been drawn from it to better prepare the critical information infrastructure and address vulnerabilities.P7

Published by HT Digital Content Services with permission from Hindustan Times. For any query with respect to this article or any other content requirement, please contact Editor at contentservices@htlive.com

Copyright (c) 2023 Hindustan Times, via HT Digital Streams Ltd. All rights reserved.

- **Citation (apa Style)**
  AIIMS attack led to new SOP for breaches: Outgoing cyber chief. (2023, July 3).*Hindustan Times (New Delhi, India)*. Available from NewsBank: Access World News: https://infoweb.newsbank.com/apps/news/document-view?p=AWNB&docref=news/19289D51C3B48450.

Title: Hospital computer hacks, like at ARH, becoming more common By: Nuzum, Lydia, Charleston Gazette, The (WV), Aug 31, 2016

Database: Points of View Reference Center

Hospital computer hacks, like at ARH, becoming more common

This content may contain URLs/links that would redirect you to a non-EBSCO site. EBSCO does not endorse the accuracy or accessibility of these sites, nor of the content therein. ✕

Listen

~~~~~~~~

Lydia Nuzum

Aug. 31--A regional health care system that operates hospitals in West Virginia and Kentucky is in the midst of a cyberattack that has crippled its electronic systems, but officials aren't yet saying how or why the system was targeted.

Appalachian Regional Healthcare, which operates two hospitals in West Virginia and nine in Kentucky, reported over the weekend that its hospital system had been the target of a cyberattack that has left employees unable to access electronic patient records, email and other automated systems.

"ARH continues to work with authorities and computer experts to address the problems and restore our systems to operational capacity as quickly as possible," ARH spokeswoman Melissa Cornett said.

She emphasized that ARH doesn't have any reason to believe that patient information, medical or financial, has been stolen, and said ARH would "take prompt action" to notify patients and employees if that did happen.
~~~~~~~~

Since Saturday, ARH employees had been tracking patients and performing their jobs without access to any of the hospitals' computerized systems, Cornett said Tuesday afternoon.

The ARH attack bears similarities to other cyberattacks perpetrated against hospitals in recent months -- at Hollywood Presbyterian Medical Center in California in February, Kansas Heart Hospital in May, and more than a dozen others, as hospitals become increasingly targeted in a hack that is growing in popularity -- "ransomware."

Ransomware is a type of software installed covertly on a computer that spreads through internet and intranet systems, encrypting information so that it no longer is accessible to users. The people behind the ransomware then demand to be paid before they restore access.

According to James Foley, manager of training and curriculum development at the National White Collar Crime Center, ransomware attacks have become increasingly sophisticated, and many hospitals, unable to break the malware's encryption, have been forced to pay up.

Hollywood Presbyterian paid $17,000 in the digital currency bitcoin to have its system unlocked, and Kansas Heart paid an initial ransom, only to have hackers demand a second ransom to break the encryption.

"The way most ransomware works is that it encrypts all of the files with an extremely difficult encryption to crack, and what you're really paying for is the key to that encryption, and if you don't pay, you don't get your files back," Foley said. "With the degree of encryptions some ransomware is able to do, there's really not a lot of chance of getting them back. There are a few versions that people have figured out how to overcome, but those are not spreading as fast as new versions that are coming out."

Asked if the hack at ARH was a ransomware attack, Cornett said the hospital system is "not at liberty to provide further information," citing the investigation with federal authorities.

Ransomware attacks have quadrupled in the past year, averaging nearly 4,000 attacks per day, according to the U.S. Justice Department. Many hacks target individuals, but businesses, universities and hospitals have increasingly become targets.

Jeremy Taylor, director of information technology at Saint Francis Hospital, in Charleston, said one common method hackers use to infect computers with ransomware is to infect the "payload" of a website and providing a link to the website in an email. When the link is opened, the malware infects the computer.

"I don't believe anyone is immune to the cyberattacks, just as I don't believe anyone's home is immune to a break-in," Taylor said. "What you have to do is your due diligence, as far as security goes, and,

hopefully, they'll move on to an easier target."

Foley said one of the best ways to safeguard against a ransomware attack is to backup entire information systems on a server that is unconnected to the entity's main system. For Taylor, education is key to ensuring that doctors, nurses and support staff within the hospital know how to recognize and avoid malware in emails and online -- a task that grows more difficult as cyberattacks become more sophisticated.

"Our weakest link is the employee that opens an email or an attachment they shouldn't have, or goes to a website and clicks on a link they shouldn't," he said. "The No. 1 thing we have to educate our users on is email use, strong passwords, and cybersecurity in general."

Reach Lydia Nuzum at lydia.nuzum@wvgazettemail.com, 304-348-5189 or follow @lydianuzum on Twitter.

___ (c)2016 The Charleston Gazette (Charleston, W.Va.) Visit The Charleston Gazette (Charleston, W.Va.) at www.wvgazette.com Distributed by Tribune Content Agency, LLC.

Copyright of **Charleston Gazette, The (WV)** is the property of Charleston Gazette, The (WV). The copyright in an individual article may be maintained by the author in certain cases. Content may not be copied or emailed to multiple sites or posted to a listserv without the copyright holder's express written permission. However, users may print, download, or email articles for individual use. **Source:** Charleston Gazette, The (WV), Aug 31, 2016
Item: 2W61136211157

Record: 1

Title: Alvarado hospital fighting cyber attack

Authors: Sisson, Paul

Source: San Diego Union-Tribune, The (CA). 04/01/2016.

Document Type: Article

Accession Number: 2W62702341238

Database: Points of View Reference Center

Alvarado hospital fighting cyber attack

~~~~~~~~
Paul Sisson

April 01--Alvarado Hospital Medical Center in San Diego is among the growing number of health care facilities nationwide coping with the effects of a malicious software attack.

Hospital spokeswoman Laura Gilbert confirmed this week that Alvarado is "resolving a malware disruption" but declined to specify which systems are affected at the 306-bed College-area facility just south of Interstate 8.

Hospitals are increasingly finding themselves under threat of "ransomware" attacks which hold critical records hostage until a ransom amount is paid. Word leaked out last week that Prime Healthcare Services, which owns Alvarado and 41 other hospitals across 14 states, detected malicious software infections at two of its California facilities -- Chino Valley Medical Center and Desert Valley Hospital in Victorville -- on Mar. 18. In statements to the Ars Technica technology website the company said that it was able to recover its computer systems without paying a ransom and that efforts to deal with the attacks caused disruptions at some of its other hospitals. Prime declined to say Thursday whether the current situation at Alvarado is a separate infection or a consequence of its malware-fighting efforts at the two northern facilities.

While Prime says it has paid no ransoms in any of the incidents, that has not been the case for other health providers.

Hollywood Presbyterian Medical Center in Los Angeles made headlines in mid-February when it reportedly paid a $17,000 ransom after malware infiltrated its systems. On Monday, MedStar Health confirmed that malicious software took down the email and patient records databases which support several Baltimore hospitals. According to the Washington Post, the coders responsible for the attack demanded 45 Bitcoins, the equivalent of $19,000, to for a digital decryption key.

In a statement to The San Diego Union-Tribune, Alvarado said the hospital and its parent company, Prime Healthcare Services, have "taken extraordinary steps to protect and expeditiously find a resolution to this disruption" but does not say what kind of trouble the infection has caused. Alvarado and Prime insist that neither patient nor employee records have been compromised.

"The hospital remains fully operational, and no patients have been turned away. All significant clinical systems needed for operations are fully functional. Our IT team took great efforts to protect and restore our systems and a ransom was never paid," Gilbert said
~~~~~~~~

The Federal Bureau of Investigation confirmed last week that it is investigating the Chino and Victorville attacks. On Thursday FBI Special Agent Darrell Foxworth confirmed that the federal government is also involved in the Alvarado incident.

"The FBI is investigating a compromising of their network to determine the groups responsible," Foxworth said, adding that a link to the Prime cases up north is being investigated but has not been formally confirmed.

Though hospitals are far from the only institutions hit recently by cyber threats -- the massive Target Co. hack of 2015 surely sticks out in many minds -- houses of healing do seem to be the juicy target of the moment.

Murray Jennex, a professor of management information systems at San Diego State University and a certified information systems security professional, said he believes hospitals are in the cross hairs of the ransomware threat because they have everything a digital extortionist is looking for.

"These are 24/7 operations, and it's difficult to shut them down, which makes them a useful target to go after. That makes it more likely they are going to just pay the ransom, because it is so difficult and painful and potentially dangerous for patients to do what's necessary to fully get rid of the malware once it has infiltrated a network," Jennex said.

The latest crop of attacks uses long-standing "phishing" techniques to infiltrate hospitals' networks. When an employee opens an infected email attachment, or visits an infected website, the malicious software quickly copies itself to other computers connected to the same network, encrypting whatever data it finds and delivering a warning to owners that their information will be deleted if they do not pay up for a digital decryption key.

It is important, Jennex said, to be ready for this kind of attack. That means making regular backups of all systems so that an administrator can simply wipe out the infected data, and replace it with clean information from a system that is not connected to the same network.

It's also important, he added, for institutions to empower their information technology staffers to take the full set of actions necessary in order to fully uproot the bug once it is detected. There have been too many instance, he said, where institutions have ignored their own network security warnings, leading to longer-lasting damage than would otherwise have occurred.

"This is what the attackers are counting on, these problems that stop you from doing the technical solution right away because they know you have to consider the business disruption as well," Jennex said.

And don't think this is just about hospitals. Jennex said many other industries are also juicy targets

"I could see them attacking utilities and financial institutions next," he said.

Just like is the case for hospitals, he said, other critical institutions have much to lose when an outside threat invades the systems now used to run every aspect of modern business.

"A big question is, if you can't trust your bank records, what will the bank do? This is just the beginning of a wave of attacks targeted at critical data," he said.

___ (c)2016 The San Diego Union-Tribune Visit The San Diego Union-Tribune at www.sandiegouniontribune.com Distributed by Tribune Content Agency, LLC.

Copyright of **San Diego Union-Tribune, The (CA)** is the property of San Diego Union-Tribune, The (CA). The copyright in an individual article may be maintained by the author in certain cases. Content may not be copied or emailed to multiple sites or posted to a listserv without the copyright holder's express written permission. However, users may print, download, or email articles for individual use.
Source: San Diego Union-Tribune, The (CA), Apr 01, 2016
Item: 2W62702341238

Print

Include when printing:

HTML Full Text (when available)

Standard Field Format Detailed Citation and Abstract

Citation Format ABNT (Brazilian National Standards)

Customized Field Format

For information on printing full text, see online help.

For information on using Citation Formats, see online citation help

Print Cancel

Title: Ransomware attacks such as at South Bend's Allied Physicians are becoming common By: Semmler, Ed, South Bend Tribune (IN), May 25, 2018

Database: Points of View Reference Center

Ransomware attacks such as at South Bend's Allied Physicians are becoming common

This content may contain URLs/links that would redirect you to a non-EBSCO site. EBSCO does not endorse the accuracy or accessibility of these sites, nor of the content therein.

Listen

~~~~~~~~
~~~~~~~~

Ed Semmler

May 25--The recent **ransomware attack** at **Allied Physicians** is **becoming** increasingly **common** across the country as thieves and even nations are looking for ways to make easy money off businesses and individuals.

In fact, it's **becoming** so **common** that all of us should assume our personal information is available to unscrupulous people and take precautions to protect ourselves, according to security experts.

"We've seen a steady increase in data breaches and data security incidents," said Doug Swetnam, section chief of the Indiana attorney general's data privacy and identity theft unit. "The number of cases has been going up about 30 percent each of the past four years."

The thieves who attacked **Allied Physicians** in **South Bend** used a variety of **ransomware** known as SamSam. The 40-**physician** practice, which operates out of University **Commons** Medical Plaza across from University Park Mall in Mishawaka, became aware of the **attack** on May 17 and immediately shut down its network.

In a release issued on Monday, the company said it was able "to restore its data in a secure format without any significant disruption of services to its patients." It also wouldn't say "whether a ransom was paid or, if so, the amount," and it indicated it is working to ensure its personal and protected information was not released.

"The security of our patients' personal and protected health information is foremost in our mind," Shery Roussarie, CEO of **Allied Physicians** said in the release. "While we make every effort to keep ahead of these types of cyberattacks, we have nevertheless taken additional steps to minimize any **such** future **attack** of the type experienced last week."

Allied Physicians said it would provide additional information as necessary in the future, but couldn't say more at this point in time since the investigation is ongoing.

Whether or not **Allied Physicians** and investigators determine personal information was not gained by the cyber criminals, it would be safest for people to assume that their information was compromised, according to security experts.

"It would be hard to prove that records haven't been made," said Scott Shackelford, a cybersecurity expert and associate professor of business law and ethics at Indiana University's Kelley School of Business.

SamSam works by gaining access to a computer system and encrypting all of the data so that it is useless unless a ransom is paid. Once that happens -- generally with Bitcoin or some other type of cryptocurrency -- thieves provide a key to unlock the data.

"This is only getting worse," said Shackelford, adding that cyberattack tools are available for rent on the dark web -- that portion of the web that generally cannot be accessed by traditional search engines.

Earlier this year, Hancock Regional Hospital in Greenfield, Ind., paid about $50,000 in Bitcoin to get its records back. According to reports, the criminals gained access to the hospital system by using a remote-access portal. Adams Memorial Hospital, southeast of Fort Wayne in Decatur, Ind., also was hit earlier this year along with other healthcare agencies and governments.

Eugene Spafford, a computer science professor at Purdue University and a leading security expert, said **such** viruses can get into a network when an employee clicks on a link in an unsolicited email. Besides when an employee accidentally opens a door, thieves also get in when the locks are bad or even non-existent -- meaning there are holes in the network software.

Businesses and individuals can protect themselves, Spafford said, by making sure they have good backups of data and that email attachments aren't opened, especially if they're unsolicited. In addition, software should be up to date to ensure windows and doors are properly bolted, and networks should be segregated so that hackers can't just go through your entire house once they break in -- in other words employ locks at each room.

"No matter how they get in, it generally involves negligent behavior," said Spafford, adding that payment of the ransom is no guarantee that you're going to get your files without paying even more ransom or that they won't be destroyed.

The threat of hackers stealing information or encrypting data with **ransomware** is among the many threats faced by businesses and individuals these days and results in higher costs for those who are vigilant.

Because of the ever-growing threat from hackers, Crowe Horwath and others provide a wide variety of help for businesses, said Jared Hamilton, leader of healthcare cybersecurity for the firm.

"**Ransomware** is **becoming** pretty prevalent," said Hamilton. "Payments are usually in the thousands of dollars, not millions. They don't want it so high that it can't be paid."

Among the many services it provides, Crowe actually will test a company's security to see if there are any vulnerabilities, make recommendations on the needed fixes and develop a response plan in the

event of an **attack**, among other things.

The attorney general's office says it is best to assume that we all could come under **attack** at some point.

"The internet is great, but it's made us accessible to criminals from all over the world," said Swetnam. "And the United States is the main target."

It's recommended that companies spend 10 percent of their IT budget on security, and consider purchasing cyber insurance in the event of a costly incident.

"If you do business in a tough neighborhood, you invest in lights, cameras and other security devices," said Swetnam. "Added security will result in higher costs of doing business and that will ultimately affect all of us."

But to ignore the problem could lead to bigger problems.

"The costs can be significant," he said. "But not to take every precaution could put you out of business."

___ (c)2018 the **South Bend** Tribune (**South Bend**, Ind.) Visit the **South Bend** Tribune (**South Bend**, Ind.) at www.southbendtribune.com Distributed by Tribune Content Agency, LLC.

Copyright of **South Bend Tribune (IN)** is the property of South Bend Tribune (IN). The copyright in an individual article may be maintained by the author in certain cases. Content may not be copied or emailed to multiple sites or posted to a listserv without the copyright holder's express written permission. However, users may print, download, or email articles for individual use. **Source:** South Bend Tribune (IN), May 25, 2018
Item: 2W63928470486

EBSCO Connect | Privacy Policy | A/B Testing | Terms of Use | Copyright | Cookie Policy | Contact Us | Manage my Cookies

powered by EBSCO*host*

© 2023 EBSCO Industries, Inc. All rights reserved.

Record: 1

AIIMS Delhi server restored from 'cyberattack', all services continue manually By: Dhar, Aniruddha. Hindustan Times. 11/29/2022. (*AN: 2W62868813170*)

Database: Points of View Reference Center

AIIMS Delhi server restored from 'cyberattack', all services continue manually

~~~~~~~~

Aniruddha Dhar

Nov. 29—All India Institute of Medical Sciences (AIIMS), Delhi, on Tuesday said its e- hospital data has been restored on servers and the network is being sanitised before services can be restored. Services at the AIIMS remained affected on the seventh consecutive day.

"The eHospital data has been restored on servers. Network being sanitized before services can be restored. The process is taking some time due to the volume of data and large number of servers/computers for the hospital services. Measures are being taken for cyber security," the AIIMS said in a statement.

"All hospital services, including outpatient, in-patient, laboratories, etc continue to run on manual mode," it added.

Earlier on Saturday, AIIMS authorities said they have deployed additional staff to run diagnostics, labs and OPD services at the national medical institute as its servers remained suspended due to a suspected ransomware attack.

It is feared that the data of around three-four crore patients could have been compromised due to the breach detected on November 23.

The Delhi Police, however, issued a statement, saying "no ransom demand as being quoted by certain sections of the media has been brought to notice by AIIMS authorities".

The India Computer Emergency Response Team (CERT-IN), Delhi Police and representatives of the ministry of home affairs are investigating the ransomware attack.

A case of extortion and cyber terrorism was registered by the Intelligence Fusion and Strategic Operations (IFSO) unit of the Delhi Police on November 25.

The AIIMS server has stored data of several VIPs, including former prime ministers, ministers, bureaucrats and judges, news agency PTI reported.

___ (c)2022 the Hindustan Times (New Delhi) Visit the Hindustan Times (New Delhi) at www.hindustantimes.com Distributed by Tribune Content Agency, LLC.

Copyright of **Hindustan Times** is the property of Hindustan Times. The copyright in an individual article may be maintained by the author in certain cases. Content may not be copied or emailed to multiple sites or posted to a listserv without the copyright holder's express written permission. However, users may print, download, or
~~~~~~~~

email articles for individual use.

Source: Hindustan Times, Nov 29, 2022

Item: 2W62868813170

Record: 1

AIIMS server outage being probed as 'cyber terrorism': Delhi Police
Hindustan Times. 11/24/2022. (*AN: 2W61305797201*)

Database: Points of View Reference Center

AIIMS server outage being probed as 'cyber terrorism': Delhi Police

~~~~~~~~

Nov. 24—Delhi Police on Thursday said they were probing the ransomware attack on the All India Institute of Medical Sciences (AIIMS) as an act of "cyber terrorism". A First Information Report (FIR) has also been lodged under various Indian Penal Code sections including that of extortion and computer-related offences.

Various government agencies are probing the incident as the hospital's server remained out of service for the second consecutive day, reported news agency PTI.

ht newsDoctors said services for out-patient (OPD) and in-patient (IPD) departments were affected and all registrations and appointment management were halted.

All emergency, routine patient care and laboratory services are being managed manually. "The registration process is being handled manually, which is causing a delay and resulting in long queues. Patients who do not have a unique health ID are facing problems," a doctor at the hospital had said.

If confirmed, this could be one of the first instances of a major Indian hospital being affected by ransomware.

These sort of attacks involve a malware that locks access to files, crippling regular functioning. Ransomware operators typically demand a payment — hence, ransom — to provide the key to decrypt the files.

(With PTI, bureau inputs)

___ (c)2022 the Hindustan Times (New Delhi) Visit the Hindustan Times (New Delhi) at www.hindustantimes.com Distributed by Tribune Content Agency, LLC.

Copyright of **Hindustan Times** is the property of Hindustan Times. The copyright in an individual article may be maintained by the author in certain cases. Content may not be copied or emailed to multiple sites or posted to a listserv without the copyright holder's express written permission. However, users may print, download, or email articles for individual use.
Source: Hindustan Times, Nov 24, 2022
Item: 2W61305797201
~~~~~~~~
