# Supplementary material for: Media Framing and Portrayals of Ransomware Impacts on Informatics, Employees, and Patients: Systematic Media Literature Review
Source: J Med Internet Res. 2025 Apr 8;27:e59231. doi: 10.2196/59231 (PMC12015346; doi:10.2196/59231)
Supplement: Multimedia Appendix 7 [file jmir_v27i1e59231_app7.docx]

**Multimedia Appendix 6: Media Portrayals of Healthcare Informatics Systems based on NIST CSF 2.0 Core Functions**

| **Article ID Example** | **NIST Cybersecurity Framework Function** | **Relationship Description** |
| --- | --- | --- |
| 688,671,334.206,76,661,57,38,150,384,664 | Governance | The article discusses an existing cybersecurity management program, enterprise risk management program, cybersecurity strategy, roles, responsibilities, authorities, policies or oversight |
| 37,447,690,57,331,38 | Identify | The article discusses or alludes to an understanding of organizations assets, and improvement opportunities for policies, plans, processes, and procedures |
| 206,671,659,661,57,61,692,15,150,545, 346,493,55,652 | Protection | The article discusses the technical and non-technical protections for the health informatic systems before, during, and after the ransomware attack, including any potential failures of protections already in place. |
| 206,447,686,688,690,14,269,16,331,150,215,691, 207,179,545,21 | Detecting | The article discusses how ransomware was detected in the health informatics systems |
| 76,206,334,659,688,37,91,14,661,16,534,331,61,692,15,38,150,215,89,9, 456,207,179,384,545,168,203,358,346,108,544,4,21,263,649,652,664 | Response | The article discusses how the organization responded or did not respond to the failure of the informatics |
| 76,334,688,37,263,649,16,57,21,534,  215,168,203,108,652,664,129,4, 543,664 | Recovery | The article discusses how the organization maintained continuity when the health informatics failed |
